# Deconvoluting interrelationships between concentrations and chemical shifts in urine provides a powerful analysis tool

**DOI:** 10.1038/s41467-017-01587-0

**Published:** 2017-11-21

**Authors:** Panteleimon G. Takis, Hartmut Schäfer, Manfred Spraul, Claudio Luchinat

**Affiliations:** 1 Giotto Biotech S.R.L., Via Madonna del Piano 6, 50019 Sesto Fiorentino (FI), Italy; 2 Bruker BioSpin, Silberstreifen, D-76287 Rheinstetten, Germany; 3 Magnetic Resonance Center (CERM), University of Florence, Via L. Sacconi 6, 50019 Sesto Fiorentino (FI), Italy; 4 Department of Chemistry Ugo Schiff, University of Florence, Via della Lastruccia 3, 50019 Sesto Fiorentino (FI), Italy

## Abstract

The NMR chemical shifts of a substance in a complex mixture strongly depend on the composition of the mixture itself, as many weak interactions occur that are hardly predictable. Chemical shift variability is the major obstacle to automatically assigning, and subsequently quantitating, metabolite signals in body fluids, particularly urine. Here we demonstrate that the chemical shifts of signals in urine are actually predictable. This is achieved by constructing ca. 4000 artificial mixtures where the concentrations of 52 most abundant urine metabolites—including 11 inorganic ions—are varied, to sparsely but efficiently populate an N-dimensional concentration matrix. A strong relationship is established between the concentration matrix and the chemical shift matrix, so that chemical shifts of > 90 metabolite signals can be accurately predicted in real urine samples. The concentrations of the invisible inorganic ions are also accurately predicted, along with those of albumin and of several other abundant urine components.

## Abstract (web summary)

The NMR chemical shifts of a substance in urine strongly depend on the composition of the mixture itself, and this makes automatic assignment for quantification very difficult. Here the authors show the chemical shifts of signals and the concentration of NMR-invisible inorganic ions in urine, are predictable.

## Introduction

Urine is extremely rich in metabolic information, containing a total of more than 2000 endogenous metabolites^1^, ~250 of which (> 400 if exogenous molecules are included) potentially detectable by NMR in minutes^2,3^. Progress toward fully automated and reliable metabolite signal identification—and subsequent quantitation—for a relevant number of metabolites would make NMR an efficient high-throughput tool for urine analysis^4^. In fact, while quantitation of metabolites by lineshape analysis of their NMR signals, once identified, is conceptually straightforward^5–9^, a major obstacle to the development of this approach is the fact that the chemical shifts of most urine metabolite signals are extremely variable, making automated identification very difficult and risky^10^. pH and some ion concentrations were already shown to contribute strongly to chemical shift variability in urine^11–13^.

Rather than accepting this variability as a fact of life, we took the view that the variability of chemical shifts of the signals of a given metabolite must be a function, no matter how complicated, of the chemical composition of the mixture as a whole, i.e., of the variable concentrations of all the metabolites in the mixture. If this relationship was unraveled, chemical shifts (*δ* hereafter) would be predictable. To test this hypothesis, we designed a strategy, which consisted in first building an extensive set of artificial urine samples with variable metabolite concentrations (concentration matrix), then constructing a corresponding matrix of chemical shifts (*δ* matrix), and finally analyzing the complex relationship between the two matrices. From this analysis, a shift predictor is developed, and then successfully tested on real urine samples.

## Results

### Construction and testing of the predictor using artificial urine samples

**Fig. 1:**
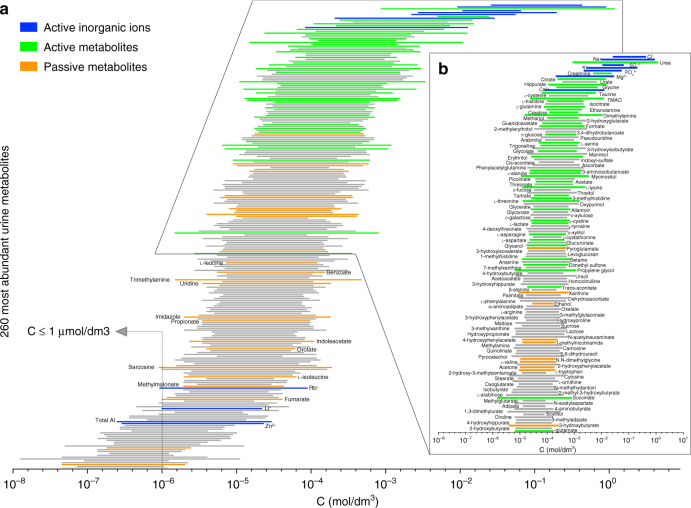
Most abundant urine metabolites and their concentration ranges in physiological conditions. Concentration ranges of the 260 most abundant metabolites in urine as determined by liquid chromatography coupled with mass spectrometry (LC-MS), NMR spectroscopy, gas chromatography with mass spectrometry (GC-MS), inductively coupled plasma mass spectrometry (ICP-MS), and high-performance liquid chromatography (HPLC)^1^. **a** Metabolites are sorted according to their mean absolute concentration values in urine. In the literature, urine metabolite concentrations are reported relative to creatinine^1^. Consequently, the absolute values were calculated by multiplying the reported minimum and maximum relative concentration of each metabolite by the minimum and maximum of creatinine, respectively. In this way, the reported ranges extend somewhat more with respect to the physiological ranges. Green bars and blue bars indicate the active metabolites and the inorganic ions (respectively) used to prepare the mother solutions of the artificial urine samples. Orange bars indicate the passive metabolites that were only spiked in the mother solutions. **b** Enlargement of the first 136 urine metabolites, with mean concentration value >30 μM. Among these metabolites, we have selected 47 (green and blue bars) as a part of the active metabolites, taking into account not only their high abundance, but also their high occurrence in urine^1^. Four additional inorganic ions (rubidium, lithium, aluminum, and zinc) were included among the active metabolites because they affect the metabolite signal chemical shifts even if they are in low abundance

**Fig. 2:**
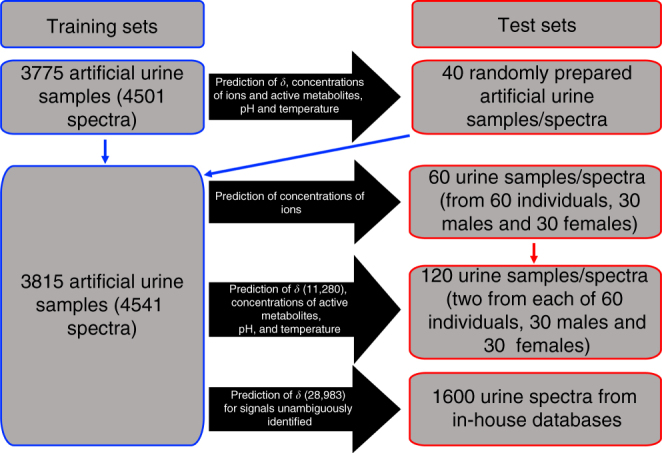
Schematic summary of artificial and real urine samples and spectra used for training and test of the predictor. Initially, 3775 artificial urine samples were prepared and their spectra recorded at 300 K. For a subset of 726 samples, selected among those exhibiting high diversity of metabolite concentrations, spectra were also recorded at 302.7 K. These samples/spectra were used to build the predictor (Supplementary Data 1; Supplementary Note 1). The predictor was tested on an independent set of 40 spectra of randomly prepared artificial urine samples. The training of the predictor was repeated by including the 40 additional samples/spectra. An independent set of 120 samples of real urine from 60 individuals was collected and their spectra used as the test set for the predictor (Supplementary Data 3). For 60 of these samples, the concentrations of 11 inorganic ions was independently measured by analytical/clinical methods and used to test the prediction of ion concentrations. Finally, another independent set of 1600 urine spectra^17–19^ was used to test the prediction of 28,983 *δ* values (Supplementary Data 4)

The metabolites that are most abundant in urine, and their normal concentration ranges, are shown in Fig. 1, ordered according to their average concentration according to literature data^1^. The strategy to construct the predictor is summarized in Fig. 2. Based on the data in Fig. 1, we prepared a large number of artificial urine samples, each containing 40 metabolites, selected among the most abundant, plus 11 inorganic ions and albumin (Supplementary Table 1; Supplementary Data 1). In each mixture, the concentrations of the 52 components were different, although all within the normal concentration range. The 40 metabolites were selected because, being usually in high concentrations, they were more likely to be active, i.e., capable of significantly modulate the *δ* values of the mixture components (Supplementary Table 2). Some of the inorganic ions were not among the most abundant metabolites but, from chemical considerations, they were still expected to interact significantly with many metabolites. Each mixture was complemented with 24 less abundant, passive metabolites, as further reporters of the effects of the active ones. Furthermore, a limited pH variability was introduced, within the small pH range allowed by the relatively strong buffer prescribed by the standard operating procedures (SOPs) for urine NMR^14^ (Methods). In total, 3775 artificial urine samples were prepared (Supplementary Data 1). NMR spectra were recorded for all these samples (Methods) at 300 K. Additional spectra were recorded for 726 of these samples at 302.7 K, for a total of 4501 spectra (Supplementary Data 1).

**Fig. 3:**
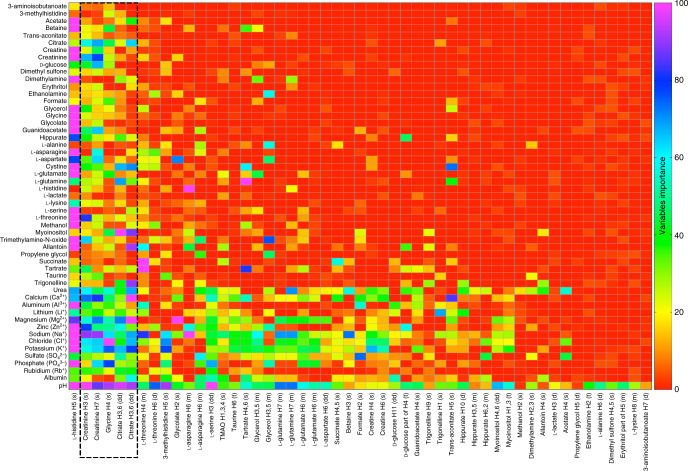
Importance of metabolite concentrations in determining chemical shifts and vice versa. The importance of the concentrations of the metabolites listed on the *y*-axis in determining the chemical shifts of the metabolites listed on the *x*-axis is shown by the colored boxes (red lowest, violet highest). The same color codes describe the importance of the knowledge of the chemical shifts of the metabolites listed on the *x*-axis in predicting the concentrations of the metabolites listed on the *y*-axis. ANOVA decomposition (Methods) of the models predicting concentrations and pH yielded the most important variables for the construction of the models themselves, as well as their relative importance for the best fitting accuracy. For instance, as depicted, chemical shifts values of 3-aminoisobutyrate H7 protons (last column) were needed for predicting the concentration of very few metabolites, and their significance was always very low (< 2), except for predicting pH (≈9). Conversely, the chemical shift of the l-histidine H5 proton (first column) is needed for the prediction of almost all metabolite concentrations as well as of pH, and appears always as a very important (20–100) variable. The 10 most frequently employed ^1^H NMR signals for model construction are from the l-histidine H5, creatinine H3 and H7, glycine, citrate H3,6, l-threonine H4 and H6, 3-methylhistidine H5, and glycolate H2 protons. The chemical shift ranges of these signals are large (Supplementary Table 1), and it appears that their variations are able to reflect the majority of the metabolite concentration (and pH) changes in the artificial urine mixtures. These relationships could reasonably be translated into chemical interactions. For instance, the imidazole signals of l-histidine and, similarly, of 3-methylhistidine are very sensitive to any pH and/or ionic change, justifying why they are almost always important variables for predicting pH and the concentration of ions. Five spin systems (dashed box) from three metabolites—two doublets of doublets of citrate, two singlets of creatinine and one singlet of glycine—are easily identifiable, very sensitive to the concentrations of the mixture components, and differently sensitive to the concentrations of different components, ensuring the broadest coverage of the concentration space. These spin systems are thus chosen as navigator signals, as they guide the search of the point in the concentration space that corresponds to that particular mixture

**Fig. 4:**
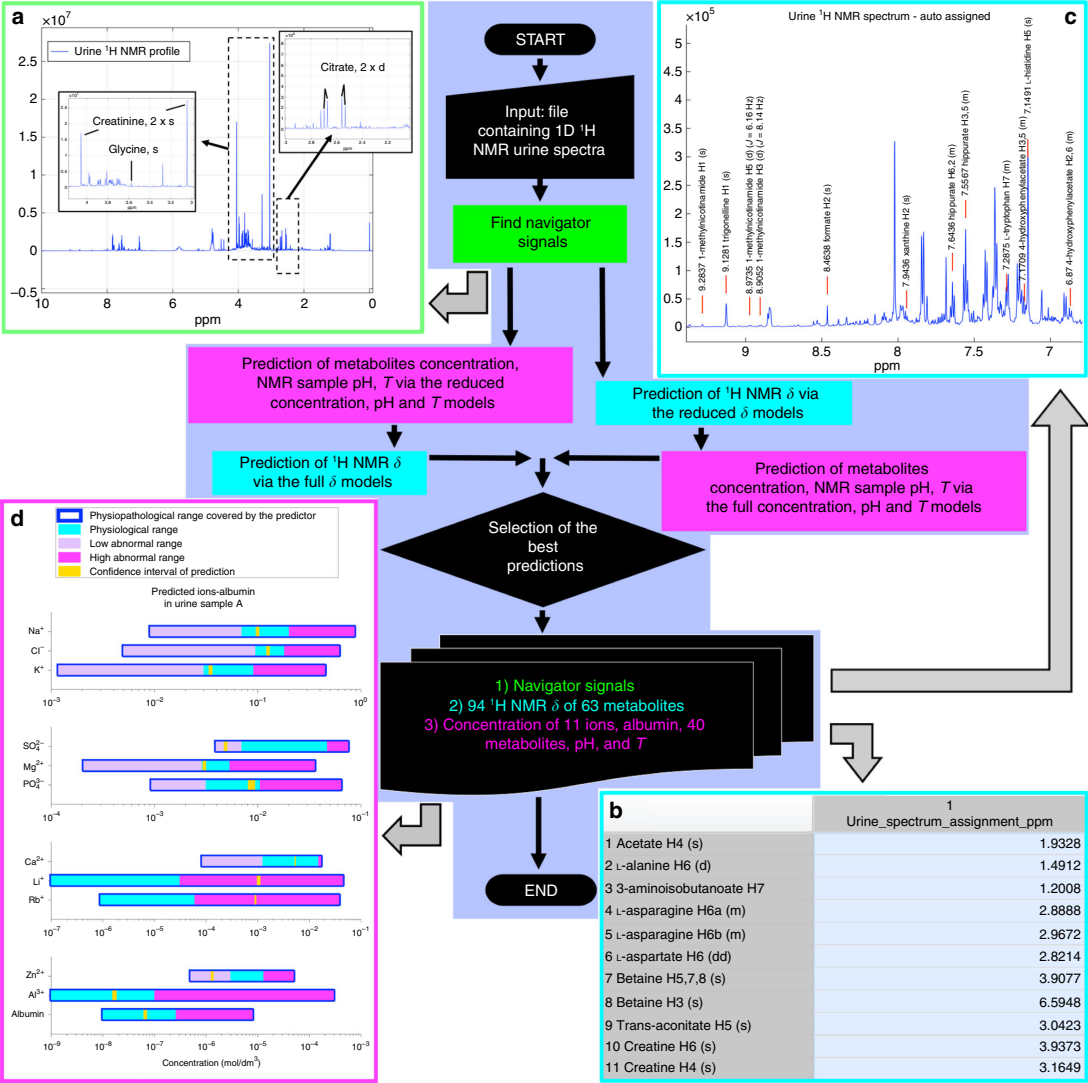
Flowchart of the shift and concentration prediction. The flowchart of the algorithm is highlighted in purple. The algorithm starts **a** upon the decision of the user about the automated or manual identification of the five navigator signals: 2 doublets of doublets (dd) of citrate, 2 singlets (s) of creatinine, and 1 singlet (s) of glycine. For citrate and creatinine specific criteria are used (e.g., the J-coupling constants of the doublets as well as the peak intensity ratios), applied to all peaks detected in a scanned spectral area where each spin system is expected to resonate (Supplementary Table 1). Since no constraints can be applied for the detection of the singlet of glycine, an extra prediction model is employed that predicts the chemical shift (*δ*) of the glycine singlet based upon the *δ* of creatinine and citrate. The prediction ability of this model was tested in 1600 different urine samples and resulted in no mis-assignments of the glycine NMR signals^17–19^. Using as input the five navigator signals, the algorithm initially employs the reduced models (based on the five navigator signals) to estimate the metabolites/ions concentrations, pH, *T*, and then predict the *δ* values for the other metabolites. Afterwards, the algorithm firstly predicts the *δ* values, and then concentrations, pH, and *T* are estimated from the former. The two routes result in two predicted *δ* values for each signal (i.e., *δ*_reduced_ and *δ*_full_), from which, according to specific selection criteria, the best results are chosen (more details in the Methods section). **b** The output of the algorithm consists of the 94 ^1^H NMR signals of 63 metabolites and **c** of a figure for each urine NMR spectrum, where the predicted *δ* of each signal along with its multiplicity is reported. **d** Moreover, one extra figure is exported comparing the predicted concentrations of 11 ions and albumin with the normal clinical range for male/female adults (based upon Mayo Clinic, www.MedlinePlus.gov and the reference guides of the laboratories that performed the clinical analyses in the present study). Two extra files contain the predicted concentrations of 11 ions and albumin, pH, and *T* values, and the estimated concentration values of 40 metabolites, respectively

From the known concentrations of ions and metabolites, and from the observed *δ* values of metabolites, the relationship between the concentration matrix and the shift matrix was constructed, as explained in the Methods section and in the Supplementary Note 1. From this relationship, given the concentration values for a given sample, the chemical shifts of the metabolite signals could be accurately predicted and, vice versa, given the pattern of chemical shifts, the concentrations could be accurately predicted. In other words, one can go from a point in the concentration space to the corresponding point in the *δ* space, and vice versa. However, since in a real sample both concentrations and shifts are unknown, it is necessary to start up the process by either providing a few concentration values or a few *δ* values, provided that the known concentrations are those of the most active metabolites or the known *δ* values are those of the most passive metabolites, i.e., of the metabolites whose *δ* values are most affected by concentration variations (Supplementary Fig. 1). This piece of information is provided by the relationship between the two matrices, and is illustrated in Fig. 3. In particular, five signals from three metabolites (citrate, creatinine, and glycine, see Supplementary Note 1 and Methods) show the desired characteristic of being very sensitive to concentration variations and at the same time easily identifiable in urine. These five signals were termed navigator signals. From the *δ* values of the navigator signals, the closest point in the *δ* space and in the concentration space can be located, and the search of the optimal relationship can start. The flowchart of the predictor is shown in Fig. 4.

The predictor was tested by independently preparing 40 artificial urine samples containing the active metabolites, ions, and albumin but with all concentrations—and pH values in the range 6.4–7.6—chosen randomly (Fig. 2). NMR spectra of these 40 samples were acquired at temperature values chosen randomly between 300 and 302.7 K. Fifty *δ* values of the active metabolites present in these 40 samples were accurately predicted (Supplementary Data 2; Supplementary Fig. 2). As explained in the Supplementary Notes 1, 2 and in the Methods, the *δ* values of all signals could be predicted from the navigators, and then the concentrations of the ions and most active metabolites originating each specific pattern of *δ* values could also be predicted (Supplementary Tables 4, 5; Supplementary Figs. 6–11). On these 40 test samples, the concentrations of the 11 inorganic ions and of several active metabolites, as well as the values of pH and temperature, could also be predicted (Supplementary Fig. 3). In particular, the prediction accuracy of the ion concentrations was very high. The spectral data set was then supplemented by the addition of the spectra of the 40 test samples, to achieve 4541 spectra (Fig. 2). A final predictor was constructed from these 4541 spectra, which turned out to be very similar to the original one. By the way it is constructed, the predictor should be able to predict the *δ* values of 94 signals from 63 metabolites. The predictor was then tested on real urine samples, as described below.

### Testing the predictor on real urine spectra—prediction of chemical shifts

The predictor was tested on an independent test set (Fig. 2) consisting of 120 real urine samples (from 60 individuals (30 males and 30 females), 2 samples each, taken on different days, see Methods section). All samples were subjected to standard preclinical treatment^14^ before the acquisition of their 600 MHz^1^H NMR spectra following the Bruker IVDr method SOPs^14–16^. As described in Supplementary Note 1, the five navigator signals from citrate, creatinine, and glycine were easily identified in all 120 spectra. All the remaining 89 signals from the other 60 metabolites were manually identified in all spectra and, whenever ambiguous, their *δ* values were checked by spiking.

As mentioned above, Fig. 4 summarizes the flowchart of the *δ* predictor. First the five navigator signals are identified, either automatically or manually. Then, *δ* predictions are performed by two routes, one going back to the concentration matrix and the other exploiting directly the internal relationships within the shift matrix (Fig. 4). By either route, all concentration values, pH, and temperature are recovered. Details of the calculations at each step of the two routes are provided in the Methods section.

**Fig. 5:**
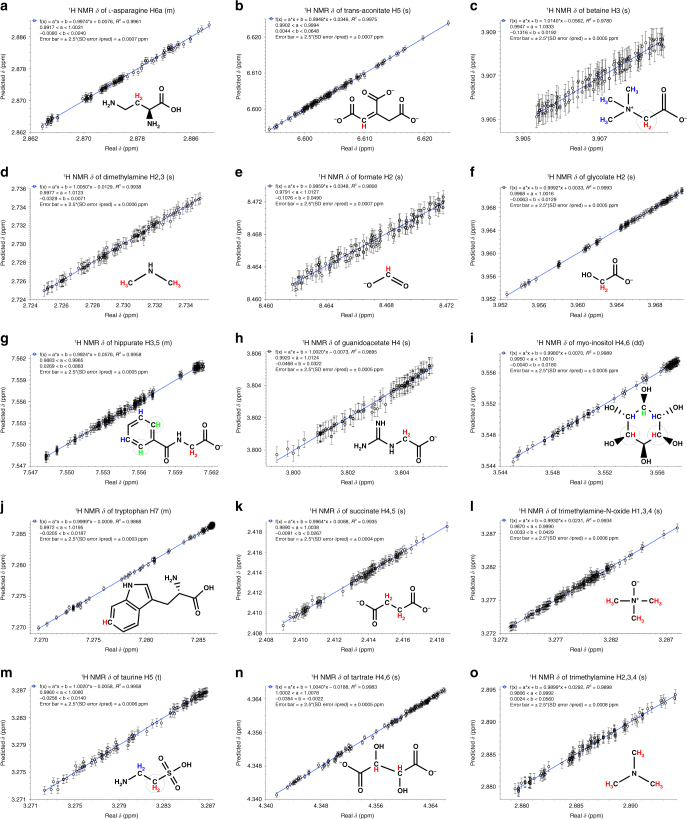
Prediction of chemical shifts (*δ*) for (**a**-**o**) 15 spin systems of 15 selected metabolites in 120 urine NMR spectra. Colored fonts and dashed circles indicate the proton spin system for which *δ* is predicted. The prediction errors are within 1.5 linewidths for all spin systems, indicating the high accuracy of all predictions tested in real urine samples. The predicted chemical shifts of additional 74 spin systems of 50 metabolites are reported in Supplementary Fig. 4

Following this procedure, the *δ* values of 94 signals of all 63 metabolites (including the metabolites containing the five navigator signals) were predicted for 120 urine samples. All the *δ* values could be predicted accurately in all spectra (Fig. 5; Supplementary Fig. 4; Supplementary Data 3). This amounts to a total of 11,280 chemical shifts that were measured and that were all accurately predicted. All linear regressions show *R* values of 98.8 or better. Typically, predictions were accurate to within 1.5 linewidths of the signals in question (Fig. 5; Supplementary Fig. 4). These results should be compared with the typical spreading in *δ* for metabolite signals in urine samples (Supplementary Fig. 1a–j; Supplementary Table 1; Supplementary Data 3): the experimental uncertainty of the *δ* prediction for each signal is in general only a few percent of the shift range where that signal can be expected to fall in urine.

Overall, out of 94 signals, only 7 were predicted with an accuracy worse than 10% of the total shift range. For all metabolites, there was at least one signal which could be predicted with an accuracy of less than two linewidths, and in at least 30 cases the accuracy was within half to one linewidth. The signals of xanthine, histidine, imidazole of the imidazole ring of 3-methylhistidine, and of the aliphatic protons of histamine constitute exceptions, as their predicted shifts are within four linewidths; however, their *δ* range is extremely large (Supplementary Fig. 4ha, pa, sc, tc, da), so their relative accuracy is also excellent.

### Testing the predictor on real urine spectra—prediction of concentrations

**Fig. 6:**
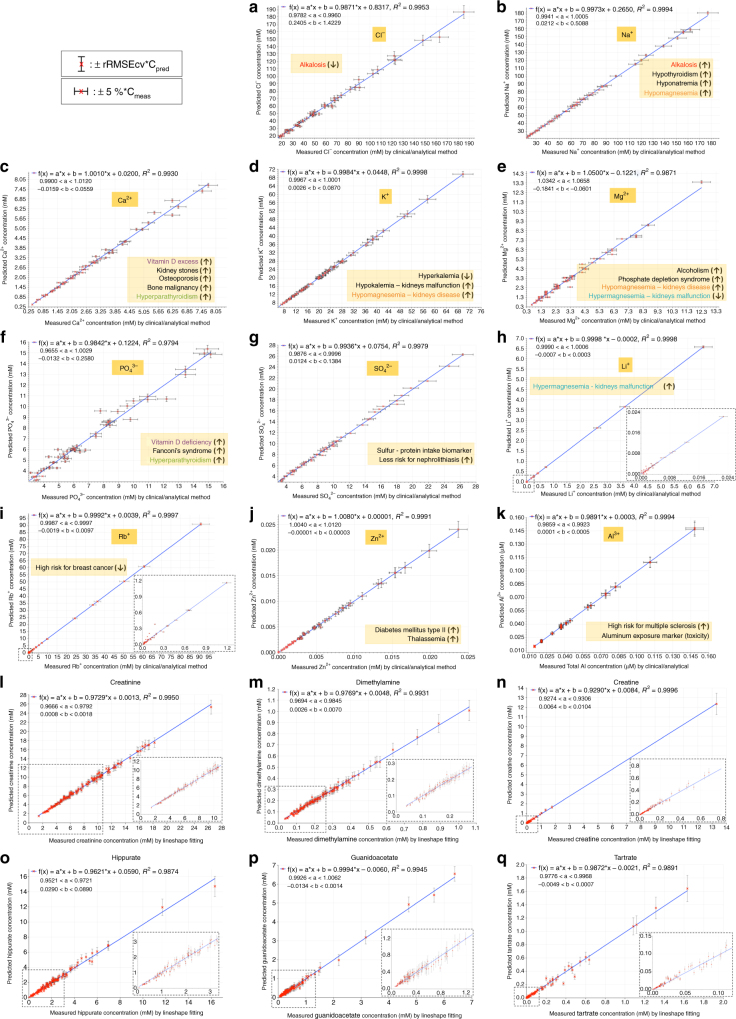
Predicted vs. measured concentrations of inorganic ions and some metabolites in real urine samples. Plots of the predicted vs. measured concentrations of 11 inorganic ions in 60 real urine samples, and of 6 among the most active metabolites in 120 real urine samples. The panels show: **a** chloride (Cl^−^), **b** sodium (Na^+^), **c** calcium (Ca^2+^), **d** potassium (K^+^), **e** magnesium (Mg^2+^), **f** phosphate (PO_4_^3−^), **g** sulfate (SO_4_^2−^), **h** lithium (Li^+^), **i** rubidium (Rb^+^), **j** zinc (Zn^2+^), **k** aluminum (Al^3+^), **l** creatinine, **m** dimethylamine, **n** creatine, **o** hippurate, **p** guanidoacetate, and **q** tartrate. Additional panels for another set of six metabolites are reported in Supplementary Fig. 5. The concentrations of the inorganic ions were determined in clinical analysis and analytical chemistry laboratories, and the metabolite concentrations were estimated by NMR lineshape fitting analyses (Methods). Horizontal error bars for ions indicate confidence intervals according to the estimated errors of the analytical technique used (Methods), and the vertical ones both for ions and active metabolites are based upon the accuracy of each predictive model, extracted from the relative root mean square error (rRMSE) values. In turn, the rRMSE values were calculated by the predictive efficiency of the models in the 40 randomly prepared artificial urine mixtures (Supplementary Fig. 3) and the 4501 artificial urine spectra. For the cases of **h** Li^+^ and **i** Rb^+^, 10 and 6 urine samples, respectively, lie outside the normal concentration range in urine, as highlighted by an additional panel. It can be seen that the models are able to predict also these outliers with high accuracy. In addition, the diseases involving alterations of each of the reported ions are reported in orange panels, with the black arrows in the parentheses showing the concentration trend of the ion for each disease

For one sample from each donor (60 samples in total), clinical/chemical analyses (Methods) were also performed to quantitate the 11 ions, invisible by NMR, selected among the active species (Fig. 2). The performance of the predictor for these NMR invisible metabolites is shown in Fig. 6a–k. It can be immediately appreciated that the predicting ability is strikingly good, and for several of these ions is excellent, as judged from the estimated uncertainties (vertical error bars), which compare very well with the uncertainties (horizontal error bars) of the clinical/chemical analyses performed by several officially adopted methods (Methods). Not all of the ions are predicted with the same precision. In fact, there is a relationship between the effect of the variable concentration of an ion on the chemical shifts of the metabolites, and the ability of back-predicting the concentration of that ion from the *δ* (Supplementary Figs. 1k–s, 2, 3). If varying the concentration of an ion had zero impact on the *δ* values of the metabolites, the concentration of that ion would be totally undetermined.

The above considerations further suggest that, besides establishing a relationship between the *δ* values and the concentration values, internal relationships within the *δ* values themselves could be established, and also explain why the latter approach is also viable (Fig. 4).

For all 120 samples, the predicted concentrations of 12 of the most active metabolites were also checked by signal lineshape fitting, and found in good agreement (Fig. 6l–q; Supplementary Fig. 5). As discussed above for the inorganic ions, only the concentrations of those metabolites that turned out to be strongly active could be predicted with good accuracy. However, this is not a drawback, as the concentrations of metabolites can be accurately measured by lineshape analysis of one or more of their signals. The bottleneck for automation of urine metabolomics is the safe and automatic identification of signals, not the quantitation of the metabolites they belong to.

### Testing the predictor on real urine spectra—prediction of chemical shifts on preexisting data sets

Finally, the predictor was tested on a large urine data set (1600 spectra, Fig. 2) available from three previous studies performed by us^17–19^. In this case the original samples were not available, so we only predicted the chemical shifts, and validated all those for which the assignment was unambiguous. In total, 28,983 signals in these data sets were unambiguously assigned, and the chemical shifts of all of them could be accurately predicted (Supplementary Table 3; Supplementary Data 4). Together with the shift predictions reported in Supplementary Data 3, more than 40,000 *δ* values were successfully predicted by our predictor.

### Potential clinical implications

Our predictor provides in a few seconds the estimated concentrations of 11 ions with clinical analysis accuracy. Therefore, any pathology that impacts on the mineral imbalance in urine^20^ could be immediately detectable with minimal costs, once the requirements for clinical adoption will be satisfied^21^. The 11 ions included so far in our predictor are relevant biomarkers for several disorders of mineral metabolism. As described in Fig. 6c, f, altered concentrations of Ca^2+^ and PO_4_^3−^ can be related to bone malignancy or damage, as well as vitamin D absorption abnormalities^20^. Instead of the four different analytical methods needed for the determination of Mg^2+^, K^+^, Na^+^, and Cl^−^ (Fig. 6a, b, d, e) to evaluate kidney function or detect the presence of hypothyroidism–hyperparathyroidism^20,22,23^, our predictor can automatically quantitate these ions. Besides the abundant ions, our predictor is also able to quantify some trace metal ions within clinical accuracy: Li^+^ (biomarker of kidney malfunction), Zn^2+^ (biomarker for diabetes mellitus^24^ and thalassemia^25^), Rb^+^ (biomarker for breast cancer risk assessment^26^), and Al^3+^ (biomarker for aluminum poisoning and multiple sclerosis risk assessment^27^). The one-shot quantitation of 11 inorganic ions is a significant feature of our predictor, prospectively allowing for the establishment of ion panels, rather than single ions, as routine biomarkers for different pathologies.

**Fig. 7:**
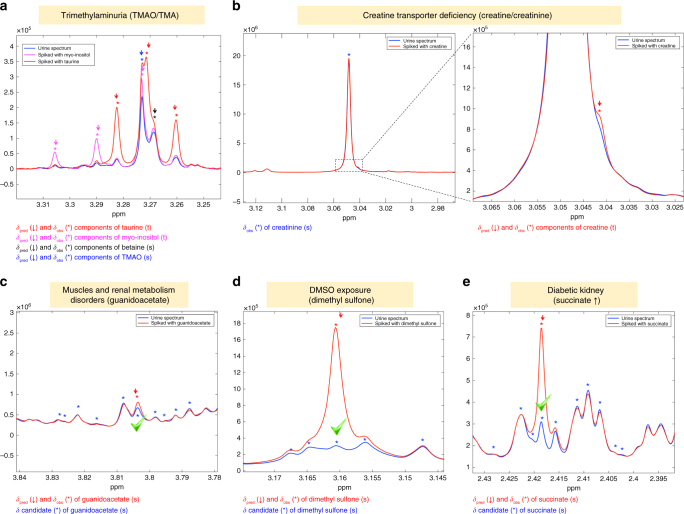
Examples of difficult metabolite assignments—and therefore quantitation—in urine. **a** TMAO and TMA are biomarkers of trimethylaminuria; however, accurate determination of TMAO concentration is challenging since many metabolite signals (*δ*_obs_) are in the same spectral region and should be properly subtracted. To do so, the position of each of them must be known accurately, and it is indeed successfully predicted (*δ*_pred_) by our predictor, leading to accurate quantitation of TMAO. **b** The creatine/creatinine concentration ratio is an important clinical parameter, but this ratio is difficult to determine due to the severe overlap between the creatine and creatinine signals. Again, it is shown that the *δ* value of creatine (although its signal is largely hidden under the strong creatinine peak) is accurately predicted. In crowded spectral areas, several unique signals of metabolites are present, making their assignment very risky. Nevertheless, our predictor always provides accurate chemical shifts predictions as shown for **c** guanidoacetate, **d** dimethyl sulfone, and **e** succinate, in perfect agreement with the observed values, validated by spiking experiments for each metabolite

The high accuracy of our predictor in predicting chemical shifts is especially valuable for metabolites whose accurate quantification is important for clinical diagnosis and that contain only one NMR signal (i.e., singlet), leading to their safe quantitation even in the absence of clues like j-splittings or intensity matching between different signals of the same molecule. For instance, to diagnose trimethylaminuria, the accurate ratio of trimethylamine (TMA) to trimethylamine N-oxide (TMAO) is required^28^. However, the only TMAO signal (one singlet at 3.27 ppm) resonates in a very complex/crowded NMR region, usually overlapped with the signals of betaine (singlet), taurine (triplet), and myo-inositol (triplet) (Fig. 7a). The identification of the hidden signals beneath TMAO as shown in Fig. 7a leads to the total deconvolution of this spectral area and to the accurate determination of TMAO concentration. A similar problem is encountered for the determination of the creatine/creatinine ratio (Fig. 7b), which is an important parameter for the X-linked inherited creatine transporter deficiency^29^. In this case, the creatine signal usually lies, in whole or in part, beneath the one of creatinine (Fig. 7b), so integration of both signals cannot be accurate, unless the signal position of creatine is known so as to make deconvolution feasible.

As already stated, the most challenging part of the assignment/quantitation of metabolites is related to those metabolites that exhibit only one singlet. The clinical value of our predictor is also demonstrated for some of these metabolites in Fig. 7c–e. For instance, the singlet of guanidoacetate, which is an important indicator for muscle and renal metabolism disorders^30,31^, is in a very crowded spectral area and is very difficult to assign, while our predictor leads to its immediate and safe identification. Another two examples are the assignment of dimethyl sulfone (important biomarker for the evaluation of exposure to DMSO^32^) and of succinate, whose high concentration in urine could indicate a diabetic kidney^33^.

## Discussion

Metabolomics of body fluids, both by MS and NMR, could make a giant step forward if signal identification and quantitation could be fully automatized. MS is more sensitive and can potentially detect above a thousand endogenous metabolite signals^34,35^, while NMR can only reach two/300^1^. However, while quantitation in MS is not straightforward, metabolite NMR signals, once identified, can be immediately translated into quantities, which makes NMR the technique of choice for front line high-throughput metabolomics screening^4^. NMR signal identification is not a severe problem for fluids that are subjected to homeostasis, like blood or cerebrospinal fluid, but is a major drawback for urine, where metabolites experience a larger variability in concentrations and therefore their signals experience a large variability in chemical shifts from one sample to another. Here, we have shown that the complex chemical matrix that constitutes urine dictates the chemical shifts of all its components in a complex but predictable way.

The first implementation of the predictor described here concerns accurate prediction of the chemical shifts of 63 metabolites, and accurate quantitation of 11 inorganic ions. Extension to more metabolites is not expected to present particular difficulties. If the present scheme is followed, about 500 spectra of artificial mixtures would need to be recorded for each group of six additional metabolites, i.e., slightly above 80 spectra per metabolite. Increasing the number of metabolites from 63 up to about 250 would thus require recording ca. 10–20,000 spectra of appropriate artificial mixtures. However, from the experience gained in this work, predictions of *δ* of additional metabolites should become increasingly faster and more accurate for two reasons: the first is that, having largely chosen the active metabolites, as well as the first groups of passive ones, with the criterion of decreasing abundance, the possibility that additional less abundant metabolites may be affecting the *δ* of the previous ones will be more and more unlikely: the second is that, with increasing number of real urine spectra, we expect that the prediction of *δ* from artificial mixtures can be finely adjusted, so that also the prediction of the shifts of the active metabolites selected originally should become more accurate. In other words, it is likely that the system will learn from experience. If so, this feature can be also automated and implemented inside our algorithm. We therefore believe that this predictor can significantly contribute to increasing both throughput and accuracy of metabolomics studies on large cohorts.

The beta-version of the predictor is available for testing at the following url: http://150.217.146.252:8080/ using a PIN code.

## Methods

### Reagents

All the chemical compounds/metabolites used for this study were purchased from Sigma-Aldrich. Before the preparation of the artificial urine mixtures, a ^1^H NMR 1D spectrum was acquired for each metabolite, to check its purity.

### Experimental procedure of urine samples collection

One hundred-twenty urine samples (1st in the morning) were collected from 60 volunteers (age range: 19–65 years old) in two random collection periods. Immediately after collection, the urine samples were used for clinical/chemical analysis (concerning the ions quantification) and their ^1^H-NMR spectra were acquired. Afterwards they were stored at −80 °C. No medical record was requested, although some of the volunteers were under medication at the time of collection. A written consent was obtained from each volunteer before providing the urine samples.

### Experimental procedure of urine samples preparation (SOPs)

According to the established protocols (SOPs)^14,15^, which were followed in previous studies^14,18,19^, 630 μl of either real or artificial urine samples were mixed with 70 μl of the standard urine D_2_O buffer (1.5 M KH_2_PO_4_ dissolved in 99.9% D_2_O, pH 7.4, 2 mM sodium azide and 0,1% 3-(trimethyl^−^silyl)propionic acid-d4 (TSP)). Finally, 600 μl out of the 700 μl was transferred into the NMR tube for spectra acquisition. According to established SOPs, the final NMR sample thus consisted of 90% urine and 10% buffer.

### ^1^H NMR spectra acquisition and processing details

Solution ^1^H NMR spectra of artificial and real urine samples were acquired using a Bruker IVDr 600 MHz spectrometer (Bruker BioSpin) operating at 600.13 MHz proton Larmor frequency and equipped with a 5 mm PATXI H/C/N with ^2^H-decoupling probe including a *z*-axis gradient coil, an automatic tuning-matching (ATM) and an automatic refrigerated sample changer (SampleJet). Temperature was regulated to 300 ± 0.1 K with a BTO 2000 thermocouple (or up to 302.7 ± 0.1 K). For each sample, a one-dimensional (1D) NMR spectrum was acquired with water peak suppression using the standard NOESY-1D presat pulse sequence^36^ (noesygppr1d; Bruker BioSpin), acquiring 32 scans (or 128 scans for the less concentrated mixtures), 65,536 data points, a spectral width of 12,019 Hz, an acquisition time of 2.7 s, a relaxation delay of 4 s, and a mixing time of 0.01 s.

Free induction decays were multiplied by an exponential function equivalent to 0.3 Hz line-broadening before applying Fourier transform. All transformed spectra were automatically corrected for phase and baseline distortions, and referenced to the TSP singlet at 0 ppm. All spectra had TSP signal linewidth, Δ*v*_1/2_ < 1 Hz.

### Construction of the chemical shift matrix

At least one ^1^H NMR signal of each active and passive metabolite was assigned in the 4501 spectra (whenever each passive metabolite was present), and their chemical shift values were recorded till at least the 5th decimal of ppm in each case. After the assignment, a multidimensional shift matrix was created (for extra details see Supplementary Table 2 and Supplementary Note 1).

### Construction of chemical shift prediction models

Under fast exchange conditions, the observed chemical shift ($\delta _0$) value of a spin system in a molecule is given by the weighted average of the chemical shifts of that spin system in each transient binary complex that the corresponding molecule can form in the mixture. The $\delta _0$ values are directly correlated to the concentration of the interacting compounds. Moreover, pH and *T* changes also cause chemical shift variations: consequently, each ^1^H-NMR $\delta _0$ value from any compound that contains ^1^H nuclei could be described by the following function:

1 $$\delta _0 = {\mathrm{f}}\left( {x_1, \ldots ,x_n} \right)$$

where the *x* variables are the concentrations of each possible interacting compound, the pH and temperature. In order to construct Eq. (1), the chemical shift matrices (see above) were employed and the multivariate adaptive regression (linear and cubic) splines models^37^ (MARS models) were applied to fit our data. By this approach, we obtained one predictive model for each chemical shift. All models exhibited very high cross-validated values (*R* ^2^_CV_) and predicting ability, along with very low root mean square errors (RMSE).

In summary, Eq. (1) for each studied ^1^H spin system took the following form^37^:

2 $$\delta _0 = c_0 + \mathop {\sum}\limits_{m = 1}^M {c_mB_m\left( x \right)} $$

where $c_0$ is the calculated constant value of the derived regression model, *M* is the number of linear or cubic spline basis function that are exploited for the production of the best fitting model, $c_m$ is the coefficient of the *m*th linear or cubic spline basis function, and $B_m\left( x \right)$ is the linear or cubic spline basis function. The calculated *R* ^2^_CV_ and RMSE values for the 94 models of the studied spin systems were > 0.98 and < 1e−04 (for l-histidine, imidazole, 3-methylhistidine, xanthine, and histamine < 5e−04, due to the high variability of their proton chemical shifts), respectively.

### Prediction of concentrations from chemical shifts

By reversing the matrices of each chemical shift from the active metabolites, a functional relationship among each concentration of the 52 active metabolites, pH plus *T* and the ^1^H NMR chemical shifts of the active metabolites is accomplished and the following equation is derived:

3 $$C,\,{\mathrm{pH}},\,T = c_0 + \mathop {\sum}\limits_{m = 1}^M {c_mB_m\left( x \right),} $$

where *C* is the concentration of each active metabolite, pH is the measured pH of each sample (after the addition of the buffer), *T* is the temperature at which each NMR experiment was performed and *x* are the ^1^H NMR chemical shifts. The other terms of Eq. (3) have the same meaning as in Eq. (2).

The calculated *R* ^2^_CV_ values for the 52 concentrations, pH, and *T* models were always > 0.90. In particular, the predictive models of the 11 ions and of the other most abundant active metabolites (e.g., creatinine, urea, hippurate, citrate, glycine, and trimethylamine-N-oxide, see Fig. 1a) exhibited very high predicting accuracy with *R* ^2^_CV_ > 0.97 (several examples in 40 randomly prepared artificial urine samples are shown in Supplementary Fig. 3).

### Determination of the navigator signals

By performing an ANOVA decomposition^37,38^ of each metabolite concentration and pH model, all weighted-important variables (i.e., ^1^H NMR chemical shifts) for the construction of each model can be determined, along with their relative statistical importance. According to Fig. 3, the chemical shifts of l-histidine H5 proton (singlet), citrate H3,6 protons (2 doublets of doublets), creatinine H3,6 protons (2 singlets), glycine H_a_ protons (singlet), l-threonine H4,6 protons (1 doublet and 1 multiplet), 3-methlyhistidine H5 proton (singlet), glycolate H2 protons (singlet), and asparagine H6 protons (2 multiplets) appear to be highly important variables for the construction of almost all concentrations and pH models. Among them, creatinine, citrate, and glycine are the most abundant (Fig. 1b), and their signals are easily identified in a normal urine ^1^H NMR profile. The rest of the metabolites signals are less easily detected, as they are frequently overlapped by other signals and/or the variability of their concentrations does not allow for their facile identification (Fig. 1b; Supplementary Table 1). Therefore, the proton signals of creatinine, glycine, and citrate (five signals in total) were employed as navigator signals.

### Chemical shifts prediction from the navigator signals

Employing as variables $\left( x \right)$ the five navigator signals and Eqs. (2), (3), new models were constructed for the direct prediction of the chemical shifts and concentration—pH values. The new models were always able to sufficiently—and often very accurately—predict the chemical shifts of the 94 spin systems from the 63 metabolites, as well as the concentrations of the 52 active metabolites, pH, and *T*. The calculated *R* ^2^_CV_ and RMSE values for the 94 models of the studied spin systems were >0.98 and < 1.5e–04, respectively (Supplementary Fig. 2c, f, i, l, o, r). The calculated *R* ^2^_CV_ values for the concentrations and pH models were > 0.80. In particular, the models of the 11 ions, of the other most abundant active metabolites (e.g., creatinine, urea, hippurate, citrate, glycine, and trimethylamine-N-oxide, see Fig. 1a), of pH and temperature, exhibited very high predicting accuracy with *R* ^2^_CV_ > 0.96 (Supplementary Fig. 3aa, ca, ea, ga, ia, ka, ma, oa, qa, sa, ua, wa, ya, ab, cb, eb, gb, ib).

### Clinical/chemical methods for the quantification of urinary ions

For the quantification of the ions in urine, several methods were used in order to estimate the average concentration values, namely, ion chromatography (IC), atomic absorption spectroscopy (AAS), electrochemical, and photometrical approaches. For all methods, initially the ions concentration in several artificial urine samples was measured, and it was compared with the theoretical value. In all cases, the quantification error was lying between 5 and 10% of the theoretical value.

In detail, for the IC determination of both cations and inorganic anions a three IC-system (Dionex) was employed^39^ (more details in ref. ^39^). The concentration of total aluminum and zinc ions was also determined by AAS (instrument: SpectrAA‐300 Zeeman, Agilent Technologies, Santa Clara, CA, USA). Chloride, potassium, and sodium ions were also measured by ion-selective electrode method (potentiometric method) using a Cobas c‐501, Roche Diagnostics, Basel, Switzerland. Calcium cations in urine were also determined by a photometric method (complexometric approach), exploiting their reaction with 5‐nitro,5′‐methyl‐BAPTA (NM-BAPTA) (instrument: Cobas c-501, Roche Diagnostics, Basel, Switzerland). The measurements took place at 376/340 nm wavelength. The same method (complexometric approach), with the same instrument, was followed to determine the concentration of phosphate anions, employing the reaction of ammonium molybdate with phosphate: {(NH_4_)_3_[PO_4_(MoO_3_)_12_]}. The measurements took place at 700/340 nm wavelength. Magnesium was determined by photometry (instrument: Cobas c‐501), through the Xylidyl blue I reagent. The measurements took place at 505/600 nm wavelength.

### Illustration of the algorithm workflow

The predictor was developed in Matlab (Mathworks programming environment). Four different kind of models, (i.e., two based on the five navigator signals (reduced models) and two on the 51 ^1^H NMR signals chemical shifts and the concentrations of the active metabolites (full models)) were tested for the best (i.e., with the lowest error) predictions. To accomplish this, 40 random artificial urine mixtures, all 4501 artificial urine training spectral data, 120 real urine samples, and a large urine data set (1600 spectra) available from two previous studies were employed (Fig. 2). After establishing a fully automated approach to accurately assign the five navigator signals in any artificial test urine spectrum (40 randomly prepared artificial urine samples), the algorithm was successfully tested in 120 plus 1600 real urine samples spectra. Thus, we employed the chemical shifts of the navigator signals as the input file of the reduced models (Fig. 4). If the predictor is unable to assign accurately one or more of the five navigator signals in a spectrum, the analysis cannot proceed. In this case, the user has the option of submitting manually the assignment of the five navigator signals. The prior knowledge of these five signals provides two output files; one with the 52 active metabolites concentrations, pH, and *T* values, and another with the 94 chemical shifts (*δ*_reduced_). The predicted *δ*_reduced_ values in the artificial urine samples exhibited an error_max_ of ±0.0002 ppm (Supplementary Fig. 2c, f, l, o, r; Supplementary Data 2), except for histidine and 3-methylhistidine:±0.0007 ppm (Supplementary Fig. 2i; Supplementary Data 2). Subsequently, the output files were used as an input for the full models, where the predictions of all chemical shifts (*δ*_full_) (Supplementary Data 2) had an error_max_ of ±0.0002 ppm (Supplementary Fig. 2d, g, m, p, s) and for histidine ±0.0005 ppm (Supplementary Fig. 2j).

When both chemical shift predictions (i.e., from the reduced and full models) fall one within the estimated uncertainty of the other, the best result is taken as their weighted average, with weights inversely proportional to the estimated uncertainty of each prediction. The uncertainty is based upon the predicting performance of the models in the 40 randomly prepared artificial mixtures and upon the relative rRMSE values of the fitted models from the 4501 artificial urine data.

So, if:

4 $$(\delta _{{\mathrm{full}}} - {\mathrm{error}}_{{\mathrm{full}}}) < (\delta _{{\mathrm{reduced}}} \pm {\mathrm{error}}_{{\mathrm{reduced}}}) < (\delta _{{\mathrm{full}}} + {\mathrm{error}}_{{\mathrm{full}}}),$$

then the weighted average *δ* is taken. The weights were estimated according to the comparison of the average rRMSE values of the *δ*_reduced_ and *δ*_full_ models from the training data (i.e., the 4501 spectra of the artificial mixtures).

If two predictions differ by more than their uncertainty, then the *δ*_reduced_ values are taken, based again upon the fact that the average rRMSE of the *δ*_reduced_ models was the lowest. Following this approach, the lowest error was observed for the independent 120 and 1600 real urine test sets plus for the total 4541 artificial urine training set (now including the 40 randomly prepared artificial urine samples).

The metabolites and ions concentration, pH, *T* models with the lowest rRMSE were employed for the best concentrations and pH, *T* values predictions. The ions concentration models were tested in the 40 independent, randomly prepared artificial urine mixtures (Supplementary Fig. 3) and 60 real urine samples (that are reported in Fig. 6) as well as in the 4501 artificial urine training data.

### Code availability

The computer code is under E.U. patent application. The predictor is available at http://150.217.146.252:8080/ and it will also be available as part of Bruker Biospin Quant-UR platform.

### Data availability

All concentration and chemical shift data are available in the Supplementary Tables 1–5 and Supplementary Data 1–4. All data is available from the authors upon reasonable request.

## Electronic supplementary material

Supplementary Information

Description of Additional Supplementary Files

Supplementary Data 1

Supplementary Data 2

Supplementary Data 3

Supplementary Data 4
