# Supplementary material for: Deconvoluting interrelationships between concentrations and chemical shifts in urine provides a powerful analysis tool
Source: Nat Commun. 2017 Nov 21;8:1662. doi: 10.1038/s41467-017-01587-0 (PMC5698486; doi:10.1038/s41467-017-01587-0)
Supplement: Supplementary file 1 — Supplementary Information [file 41467_2017_1587_MOESM1_ESM.pdf]

Supplementary Table 1. Concentration ranges of metabolites, ions and albumin in the 3775 artificial urine mixtures, along with 94 ^{1}H NMR chemical shifts (δ) ranges of the 63 metabolites (including the spiked), observed in the corresponding NMR spectra. The δ value range of each assigned ^{1}H NMR spin system is reported with the same colour code as in the molecular structure of each metabolite. The NMR signal multiplicity is denoted as (s) for singlet, (d) for doublet, (t) for triplet, (dd) for doublet of doublets, (q) for quartet and (m) for multiplet.

Metabolite	Concentration range (min – max) (mol/dm^3 x 10^{-3})	Molecular structure	chemical shift range (min – max) observed for each spin system highlighted in the structure (ppm)
3-aminoisobutanoate	0.0000 – 3.5000	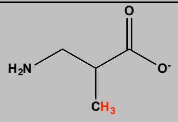	1.1950 – 1.2006 (d)
3-methylhistidine	0.0130 – 1.4936	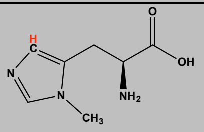	7.0440 – 7.2950 (s)
acetate	0.1330 – 2.9870	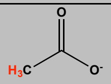	1.9230 – 1.9337 (s)
betaine	0.0141 – 0.6164	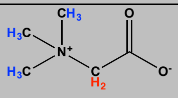	3.2663 – 3.2726 (s)<br>3.9033 – 3.9123 (s)
trans-aconitate	0.0185 – 2.3728	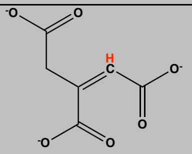	6.5823 – 6.6240 (s)
citrate	0.0000 – 15.0869	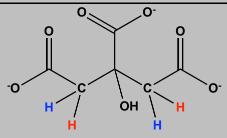	2.5252 – 2.5907 (d)<br>2.6417 – 2.7201 (d)
creatine	0.0002 – 14.1900	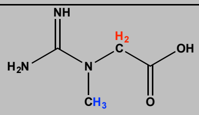	3.0395 – 3.0462 (s)<br>3.9332 – 3.9432 (s)
creatinine	0.0000 – 24.9774	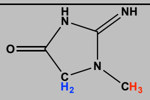	3.0406 – 3.0583 (s)<br>4.0521 – 4.0785 (s)
D-glucose	0.0004 – 0.9596	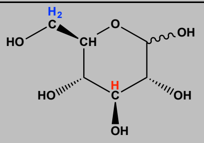	3.4984 – 3.5160 (t)<br>3.9050 – 3.9120 (dd)
dimethyl sulfone	0.0062 – 1.2337	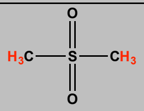	3.1581 – 3.1678 (s)

**dimethylamine**	**0.0015 – 1.4888**	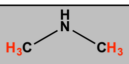	**2.7207 – 2.7348 (s)**
**erythritol**	**0.0335 – 1.6086**	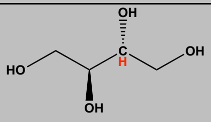	**3.6869 – 3.7028 (m)**
**ethanolamine**	**0.1205 – 1.4137**	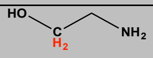	**3.8317 – 3.8417 (m)**
**formate**	**0.0322 – 3.0323**	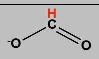	**8.4590 – 8.4732 (s)**
**glycerol**	**0.0195 – 0.4845**	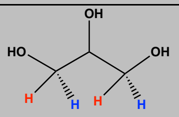	**3.5677 – 3.5745 (m)**<br>**3.6601 – 3.6707 (m)**
**glycine**	**0.0000 – 7.4929**	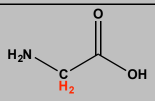	**3.5668 – 3.5823 (s)**
**glycolate**	**0.0197 – 3.0504**	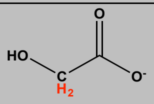	**3.9496 – 3.9725 (s)**
**guanidoacetate**	**0.0010 – 8.4335**	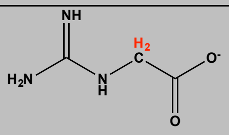	**3.7994 – 3.8067 (s)**
**hippurate**	**0.0005 – 15.5339**	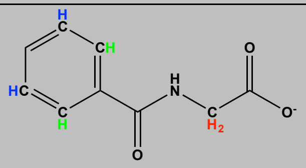	**3.9693 – 3.9762 (d)**<br>**7.5485 – 7.5642 (m)**<br>**7.6359 – 7.6517 (m)**
**L-alanine**	**0.0330 – 1.0839**	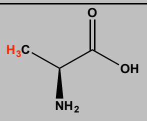	**1.4831 – 1.4923 (d)**
**L-asparagine**	**0.0220 – 0.4446**	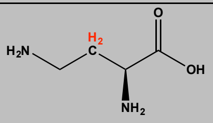	**2.8638 – 2.8980 (dd)**<br>**2.9506 – 2.9667 (dd)**
**L-aspartate**	**0.0058 – 0.6815**	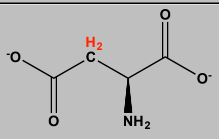	**2.8100 – 2.8216 (dd)**
**cystine (L-cysteine oxidation)**	**0.1142 – 3.3602**	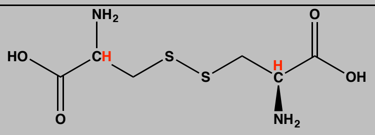	**4.0925 – 4.1436 (dd)**
**L-glutamate**	**0.0000 – 0.4596**	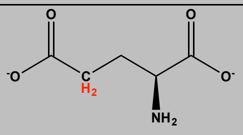	**2.3366 – 2.3495 (m)**<br>**2.3678 – 2.3810 (m)**

**L-glutamine**	**0.0804 – 1.9457**	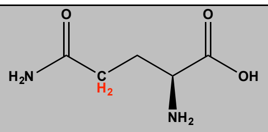	**2.4386 – 2.4477 (m)**<br>**2.4796 – 2.4879 (m)**
**L-histidine**	**0.0801 – 2.2479**	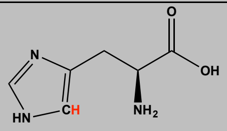	**7.0794 – 7.2693 (d)**
**L-lactate**	**0.0003 – 0.9493**	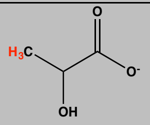	**1.3329 – 1.3432 (s)**
**L-lysine**	**0.0180 – 1.2814**	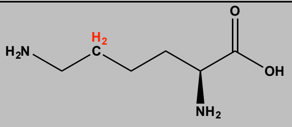	**1.7320 – 1.7388 (m)**
**L-serine**	**0.0504 – 1.4338**	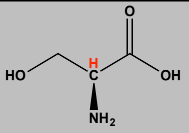	**3.8505 – 3.8735 (m)**
**L-threonine**	**0.0310 – 0.6294**	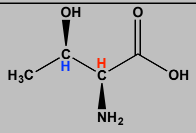	**3.5898 – 3.6228 (d)**<br>**4.2563 – 4.2853 (m)**
**methanol**	**0.0462 – 2.9224**	H_3C—OH	**3.3650 – 3.3710 (s)**
**myoinositol**	**0.0319 – 0.9017**	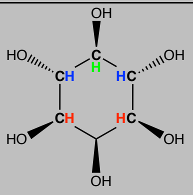	**3.5434 – 3.5576 (dd)**<br>**3.6283 – 3.6391 (t)**<br>**3.2880 – 3.3070 (t)**
**trimethylamine-N-oxide**	**0.0203 – 12.7135**	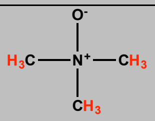	**3.2692 – 3.2875 (s)**
**allantoin**	**0.0152 – 4.5739**	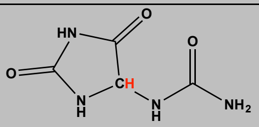	**5.3900 – 5.4050 (s)**
**propylene glycol**	**0.0070 – 1.1165**	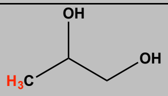	**1.1452 – 1.1500 (d)**
**succinate**	**0.0010 – 0.8317**	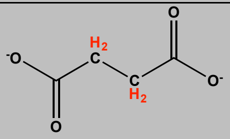	**2.4071 – 2.4228 (s)**
**tartrate**	**0.0007 – 1.6085**	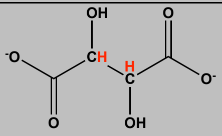	**4.3379 – 4.3662 (s)**

taurine	0.0004 – 6.2694	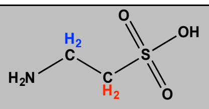	3.4220 – 3.4428 (t)<br>3.2665 – 3.2870 (t)
trigonelline	0.0023 – 2.7301	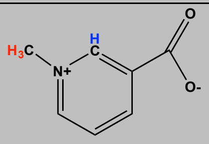	4.4413 – 4.4520 (s)<br>9.1200 – 9.1326 (s)
urea	0.8094 – 1223.9000	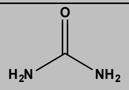	very broad ^{1}H NMR signal
Spiked metabolites			
fumarate		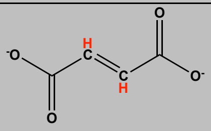	6.5224 – 6.5336 (s)
N,N-dimethylglycine		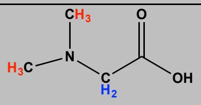	2.9280 – 2.9385 (s)<br>3.7236 – 3.7354 (s)
acetone		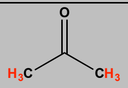	2.2362 – 2.2434 (s)
3-hydroxybutyrate		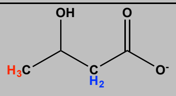	1.2030 – 1.2090 (s)<br>2.3100 – 2.3195 (m)
ethanol		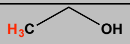	1.1872 – 1.1925 (t)
sarcosine		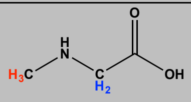	2.7431 – 2.7533 (s)<br>3.6147 – 3.6267 (s)
L-isoleucine		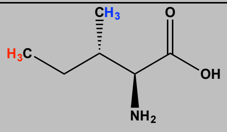	0.9410 – 0.9444 (t)<br>1.0120 – 1.0170 (d)
benzoate		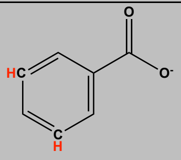	7.4860 – 7.4962 (m)
L-tryptophan		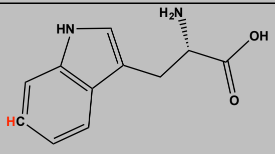	7.2698 – 7.2946 (m)
L-valine		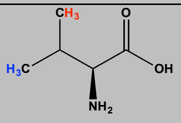	0.9921 – 0.9994 (d)<br>1.0435 – 1.0512 (d)

**4-hydroxyphenylacetate**		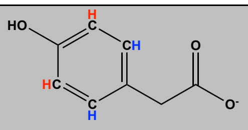	**6.8631 – 6.8763 (m)**<br>**7.1634 – 7.1730 (m)**
**L-leucine**		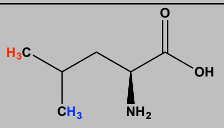	**0.9573 – 0.9621 (d)**<br>**0.9689 – 0.9727 (d)**
**1-methylnicotinamide**		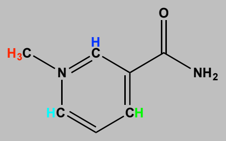	**4.4800 – 4.4920 (s)**<br>**8.8920 – 8.9130 (d)**<br>**8.9687 – 8.9810 (d)**<br>**9.2690 – 9.2907 (s)**
**orotate**		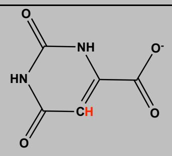	**6.1829 – 6.2000 (s)**
**methylmalonate**		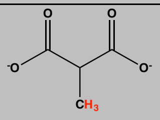	**1.2381 – 1.2584 (d)**
**pyrogutamate**		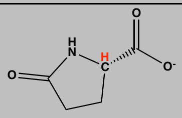	**4.1801 – 4.1891 (dd)**
**1,3-dimethylurea**		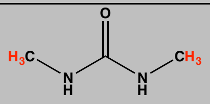	**2.6829 – 2.6917 (s)**
**xanthine**		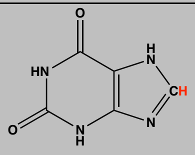	**7.8653 – 7.9755 (s)**
**uridine**		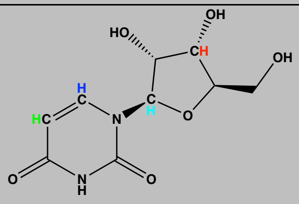	**4.2360 – 4.2426 (t)**<br>**5.9055 – 5.9198 (d)**<br>**5.9174 – 5.9273 (d)**<br>**7.8648 – 7.8760 (d)**
**propionate**		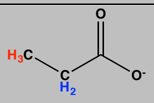	**1.0564 – 1.0643 (t)**<br>**2.1855 – 2.1952 (q)**
**trimethylamine**		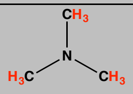	**2.8762 – 2.8991 (s)**
**indoleacetate**		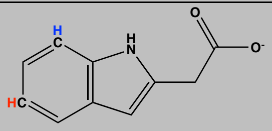	**7.2417 – 7.2628 (m)**<br>**7.5004 – 7.5301 (d)**

histamine		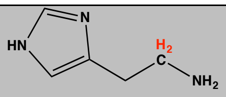	3.2935 – 3.3360 (t)
imidazole		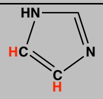	7.2783 – 7.4435 (s)
Ions - albumin			
Calcium (Ca^{2+})	0.0839 – 16.9881		No ^{1}H NMR signals
Aluminum (Al^{3+})	≈ 0.0000 – 0.2982		
Lithium (Li^{+})	≈ 0.0000 – 45.0800		
Magnesium (Mg^{2+})	0.2061 – 35.8229		
Zinc (Zn^{2+})	0.0005 – 0.0500		
Sodium (Na^{+})	9.0987 – 868.8675		
Chloride (Cl^{+})	5.0000 – 618.5036		
Potassium (K^{+})	1.1734 – 450.5641		
Sulfate (SO_4^{2-})	3.9361 – 75.4379		
Phosphate (PO_4^{3-})	0.9329 – 64.4067		
Rubidium (Rb^{+})	0.0009 – 35.9107		
Albumin	0.0000 – 0.0075		

Supplementary Table 2. Chemical shift matrix of L-histidine H5 proton as an example. The full matrix consists of 54 columns and 1775 rows.

Chemical shift (ppm) for L-histidine H5 proton)	Citrate (mM)	Creatinine (mM)	Mg^{2+} (mM)	K^{+} (mM)	SO_4^{2-} (mM)	…	PO_4^{3-} (mM)	pH	No. of mixtures/ cases
**7.0794**	0.24	4.95	0.21	27.35	10.73		0.93	7.45	1
**7.0944**	14.99	24.98	29.72	201.79	67.44		24.41	7.54	2
**7.0965**	0.24	4.95	0.21	177.71	10.73		0.93	7.43	3
**7.0967**	14.99	20.00	29.73	201.79	67.44		24.41	7.53	4
**7.0978**	14.99	24.98	29.73	89.74	67.44		24.41	7.53	5
**7.0990**	3.04	14.96	3.92	53.76	36.02		8.72	7.36	6
**7.1011**	0.24	4.95	0.21	27.35	10.73	…	0.93	7.26	7
**7.1041**	0.24	4.95	0.21	27.35	10.73		0.93	6.68	8
**7.1045**	3.04	14.00	17.79	53.76	36.02		8.72	7.39	9
**7.1106**	0.73	14.96	3.92	53.76	36.02		8.72	7.24	10
**7.1317**	0.24	4.95	0.21	27.35	10.73		0.93	7.00	11
			⋮					⋮	
**7.2693**	14.99	24.98	29.73	201.79	54.20		24.41	6.44	1775

Supplementary Table 3. Test of the predictor on a large urine dataset (1600 spectra) available from previous studies[1–3]. The third column reports the number of spectra, out of 1600, where the predicted chemical shifts are validated by unambiguous assignment.

Metabolite	Molecular structure	Number spectra
**3-aminoisobutanoate**	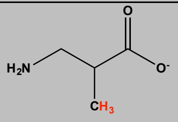	**97**
**3-methylhistidine**	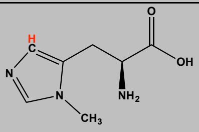	**111**
**acetate**	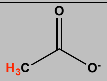	**455**
**betaine**	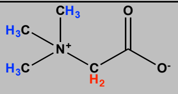	**326**
**trans-aconitate**	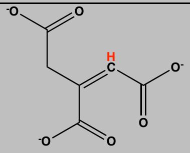	**32**
**citrate**	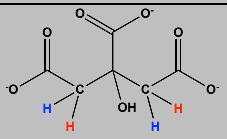	**1600**
**creatine**	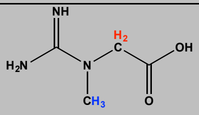	**411**
**creatinine**	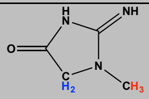	**1600**
**D-glucose**	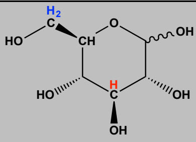	**113**
**dimethyl sulfone**	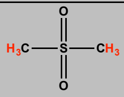	**97**
**dimethylamine**	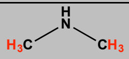	**1407**
**erythritol**	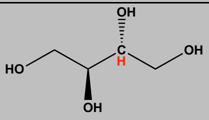	**17**

**ethanolamine**	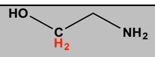	**19**
**formate**	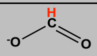	**1357**
**glycerol**	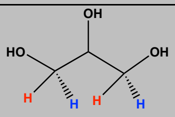	**148**
**glycine**	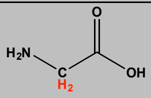	**1600**
**glycolate**	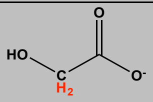	**656**
**guanidoacetate**	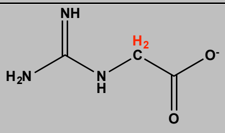	**228**
**hippurate**	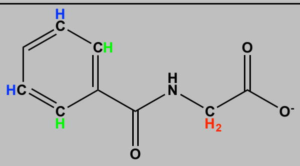	**1216**
**L-alanine**	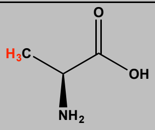	**1356**
**L-histidine**	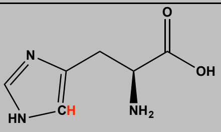	**143**
**L-lactate**	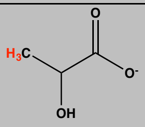	**293**
**L-serine**	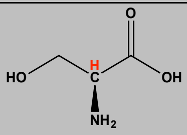	**171**
**L-threonine**	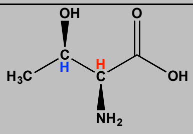	**198**
**methanol**	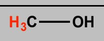	**360**
**trimethylamine-N-oxide**	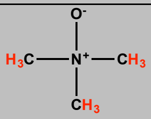	**956**

**allantoin**	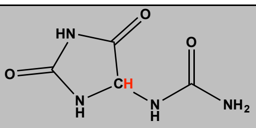	**165**
**succinate**	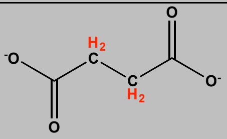	**166**
**tartrate**	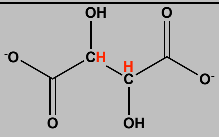	**221**
**taurine**	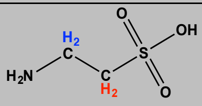	**157**
**trigonelline**	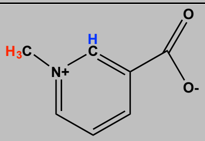	**963**
**acetone**	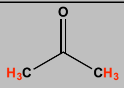	**153**
**L-valine**	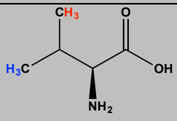	**77**
**4-hydroxyphenylacetate**	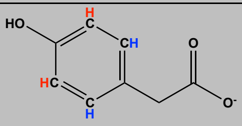	**158**
**1-methylnicotinamide**	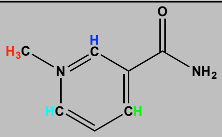	**647**
**L-leucine**	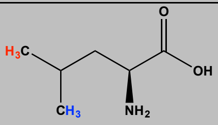	**67**
**L-isoleucine**	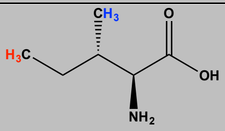	**133**
**myoinositol**	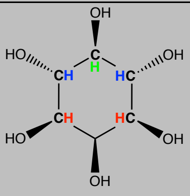	**14**

**L-asparagine**	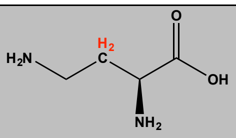	**22**
**L-aspartate**	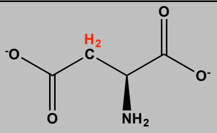	**14**
**L-glutamate**	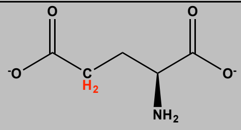	**42**
**L-glutamine**	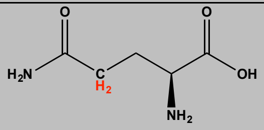	**28**
**ethanol**	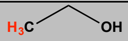	**15**
**fumarate**	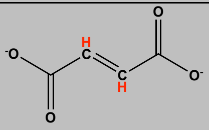	**47**
**propylene glycol**	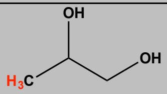	**9**
**3-hydroxybutyrate**	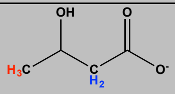	**17**
**L-lysine**	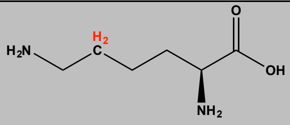	**37**
**orotate**	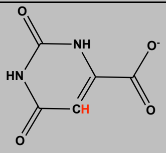	**0**
**benzoate**	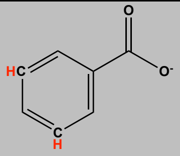	**35**
**L-tryptophan**	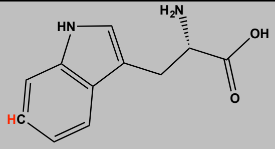	**12**
**sarcosine**	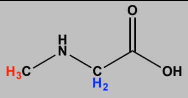	**22**

**methylmalonate**	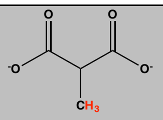	8
**N,N-dimethylglycine**	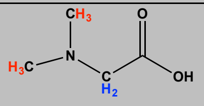	33
**pyrogutamate**	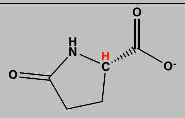	31
**cystine**	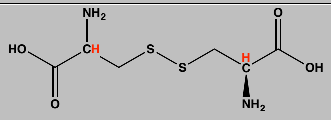	4
**1,3-dimethylurea**	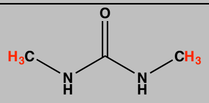	0
**xanthine**	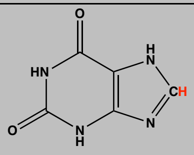	44
**uridine**	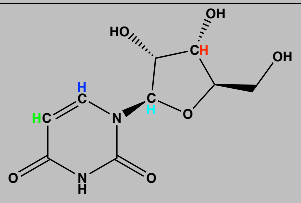	21
**propionate**	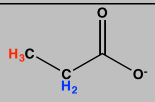	18
**trimethylamine**	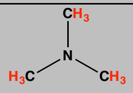	52
**indoleacetate**	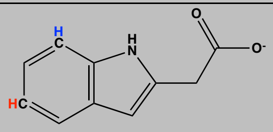	9
**histamine**	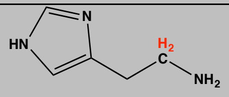	6
**imidazole**	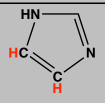	5
	**Total Number of spin systems measured and assigned**	**28983**

Supplementary Table 4. The selected 5 different concentrations of 3 ions (Li^+, Na^+ and K^+) used to build the concentration matrix described in Supplementary Note 2. The counter ion for all cations was Cl^-.

Na^+ (mM)	K^+ (mM)	Li^+ (mM)
10	10	0.2
30.72	25.85	5
94.4	66.83	15.85
290.06	172.78	25.12
890	446.68	40

Supplementary Table 5. One hundred twenty-five artificial mixtures of 12 metabolites (at fixed concentration) containing 3 ions (Li^+, Na^+ and K^+) in the form of chloride salts, at variable concentrations (see Supplementary Note 2).

	**Ions concentration (mM)**			
**Number of mixture**	Na^+	K^+	Li^+	Cl^-
**1**	10	10	0.2	20.2
**2**	10	10	5	25
**3**	10	10	15.85	35.85
**4**	10	10	25.12	45.12
**5**	10	10	40	60
**6**	10	25.85	0.2	36.05
**7**	10	25.85	5	40.85
**8**	10	25.85	15.85	51.7
**9**	10	25.85	25.12	60.97
**10**	10	25.85	40	75.85
**11**	10	66.83	0.2	77.03
**12**	10	66.83	5	81.83
**13**	10	66.83	15.85	92.68
**14**	10	66.83	25.12	101.95
**15**	10	66.83	40	116.83
**16**	10	172.78	0.2	182.98
**17**	10	172.78	5	187.78
**18**	10	172.78	15.85	198.63
**19**	10	172.78	25.12	207.9
**20**	10	172.78	40	222.78
**21**	10	446.68	0.2	456.88
**22**	10	446.68	5	461.68
**23**	10	446.68	15.85	472.53
**24**	10	446.68	25.12	481.8
**25**	10	446.68	40	496.68
**26**	30.72	10	0.2	40.92
**27**	30.72	10	5	45.72
**28**	30.72	10	15.85	56.57
**29**	30.72	10	25.12	65.84
**30**	30.72	10	40	80.72
**31**	30.72	25.85	0.2	56.77
**32**	30.72	25.85	5	61.57
**33**	30.72	25.85	15.85	72.42

**34**	30.72	25.85	25.12	81.69
**35**	30.72	25.85	40	96.57
**36**	30.72	66.83	0.2	97.75
**37**	30.72	66.83	5	102.55
**38**	30.72	66.83	15.85	113.4
**39**	30.72	66.83	25.12	122.67
**40**	30.72	66.83	40	137.55
**41**	30.72	172.78	0.2	203.7
**42**	30.72	172.78	5	208.5
**43**	30.72	172.78	15.85	219.35
**44**	30.72	172.78	25.12	228.62
**45**	30.72	172.78	40	243.5
**46**	30.72	446.68	0.2	477.6
**47**	30.72	446.68	5	482.4
**48**	30.72	446.68	15.85	493.25
**49**	30.72	446.68	25.12	502.52
**50**	30.72	446.68	40	517.4
**51**	94.4	10	0.2	104.6
**52**	94.4	10	5	109.4
**53**	94.4	10	15.85	120.25
**54**	94.4	10	25.12	129.52
**55**	94.4	10	40	144.4
**56**	94.4	25.85	0.2	120.45
**57**	94.4	25.85	5	125.25
**58**	94.4	25.85	15.85	136.1
**59**	94.4	25.85	25.12	145.37
**60**	94.4	25.85	40	160.25
**61**	94.4	66.83	0.2	161.43
**62**	94.4	66.83	5	166.23
**63**	94.4	66.83	15.85	177.08
**64**	94.4	66.83	25.12	186.35
**65**	94.4	66.83	40	201.23
**66**	94.4	172.78	0.2	267.38
**67**	94.4	172.78	5	272.18
**68**	94.4	172.78	15.85	283.03
**69**	94.4	172.78	25.12	292.3
**70**	94.4	172.78	40	307.18
**71**	94.4	446.68	0.2	541.28
**72**	94.4	446.68	5	546.08
**73**	94.4	446.68	15.85	556.93
**74**	94.4	446.68	25.12	566.2
**75**	94.4	446.68	40	581.08
**76**	290.06	10	0.2	300.26
**77**	290.06	10	5	305.06
**78**	290.06	10	15.85	315.91
**79**	290.06	10	25.12	325.18
**80**	290.06	10	40	340.06
**81**	290.06	25.85	0.2	316.11
**82**	290.06	25.85	5	320.91
**83**	290.06	25.85	15.85	331.76

**84**	290.06	25.85	25.12	341.03
**85**	290.06	25.85	40	355.91
**86**	290.06	66.83	0.2	357.09
**87**	290.06	66.83	5	361.89
**88**	290.06	66.83	15.85	372.74
**89**	290.06	66.83	25.12	382.01
**90**	290.06	66.83	40	396.89
**91**	290.06	172.78	0.2	463.04
**92**	290.06	172.78	5	467.84
**93**	290.06	172.78	15.85	478.69
**94**	290.06	172.78	25.12	487.96
**95**	290.06	172.78	40	502.84
**96**	290.06	446.68	0.2	736.94
**97**	290.06	446.68	5	741.74
**98**	290.06	446.68	15.85	752.59
**99**	290.06	446.68	25.12	761.86
**100**	290.06	446.68	40	776.74
**101**	890	10	0.2	900.2
**102**	890	10	5	905
**103**	890	10	15.85	915.85
**104**	890	10	25.12	925.12
**105**	890	10	40	940
**106**	890	25.85	0.2	916.05
**107**	890	25.85	5	920.85
**108**	890	25.85	15.85	931.7
**109**	890	25.85	25.12	940.97
**110**	890	25.85	40	955.85
**111**	890	66.83	0.2	957.03
**112**	890	66.83	5	961.83
**113**	890	66.83	15.85	972.68
**114**	890	66.83	25.12	981.95
**115**	890	66.83	40	996.83
**116**	890	172.78	0.2	1062.98
**117**	890	172.78	5	1067.78
**118**	890	172.78	15.85	1078.63
**119**	890	172.78	25.12	1087.9
**120**	890	172.78	40	1102.78
**121**	890	446.68	0.2	1336.88
**122**	890	446.68	5	1341.68
**123**	890	446.68	15.85	1352.53
**124**	890	446.68	25.12	1361.8
**125**	890	446.68	40	1376.68

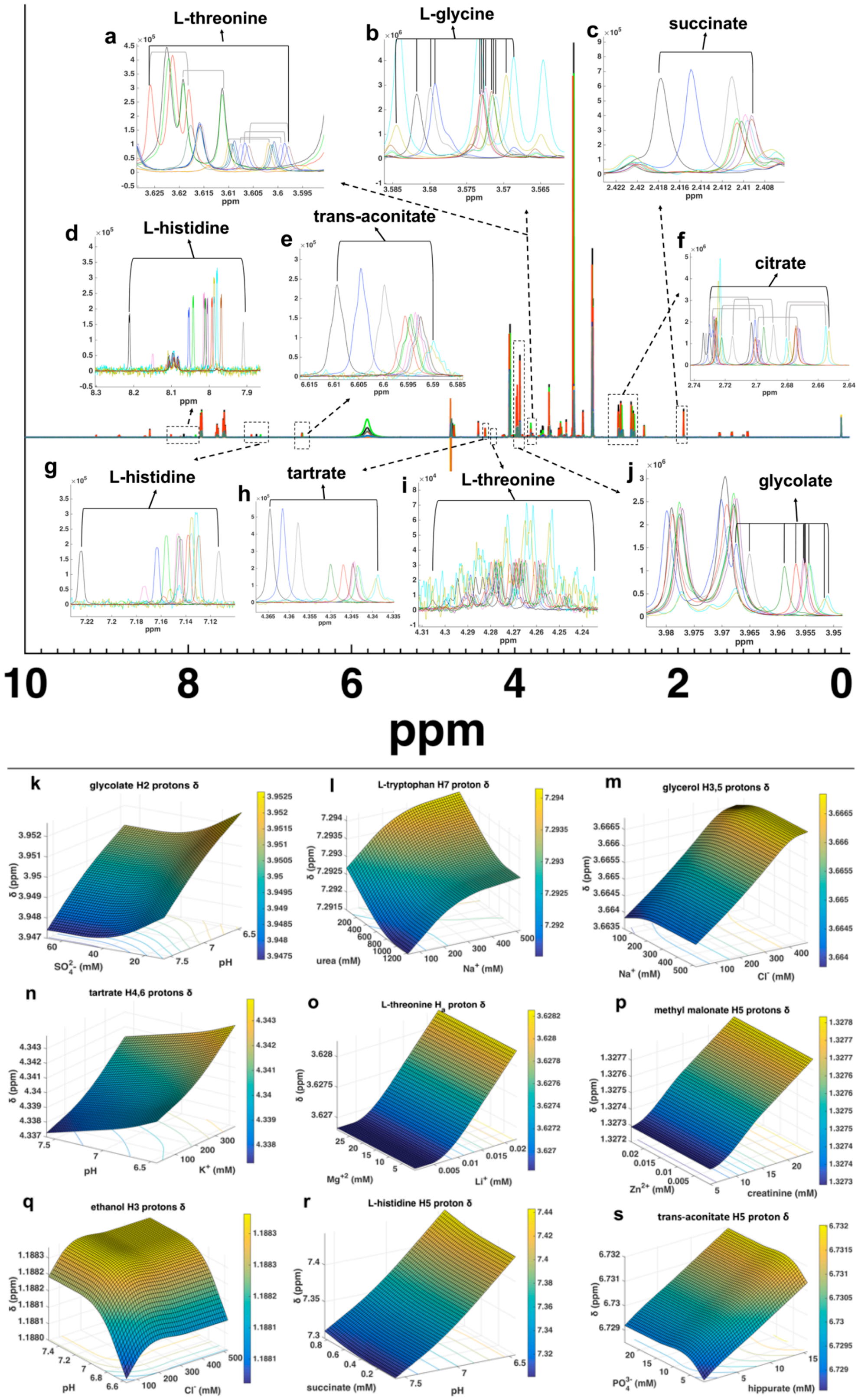

Supplementary Figure 1. Complex relationships among metabolite concentrations and metabolite chemical shifts in artificial urine. (**a-j**) Variability of selected δ values in the artificial mixtures: (**a**) L-threonine Ha protons, (**b**) glycine Ha protons, (**c**) succinate H4,5 protons, (**d**) L-histidine imidazole ring H2 proton, (**e**) trans-aconitate H5 proton, (**f**) citrate H3,6 protons, (**g**) L-histidine H5 proton, (**h**) tartrate H4,6 protons, (**i**) L-threonine H6 protons (**j**) glycolate H2 protons. (**k-s**) Nine panels

demonstrating the different effects of changes of concentration of ions or of other metabolites, or of changes of pH, on the δ values of selected signals of some metabolites. The interpolated δ values are obtained from the constructed statistical predictive models (see Methods): impact of (**k**) sulfate (SO_4^{2-}) and pH on glycolate H2 protons, (**l**) of sodium (Na^+) and urea on tryptophan H7 proton, (**m**) of Na^+ and chloride (Cl^-) on glycerol H3,5 protons, (**n**) of potassium (K^+) and pH on tartrate H4,6 protons, (**o**) of magnesium (Mg^{2+}) and lithium (Li^+) on L-threonine Ha proton, (**p**) of zinc (Zn^{2+}) and creatinine on methylmalonate H5 protons, (**q**) of Cl^- and pH on ethanol H3 protons, (**r**) of succinate and pH on L histidine H5 proton, and (**s**) of phosphate (PO_4^{3-}) and hippurate on trans-aconitate H5 proton.

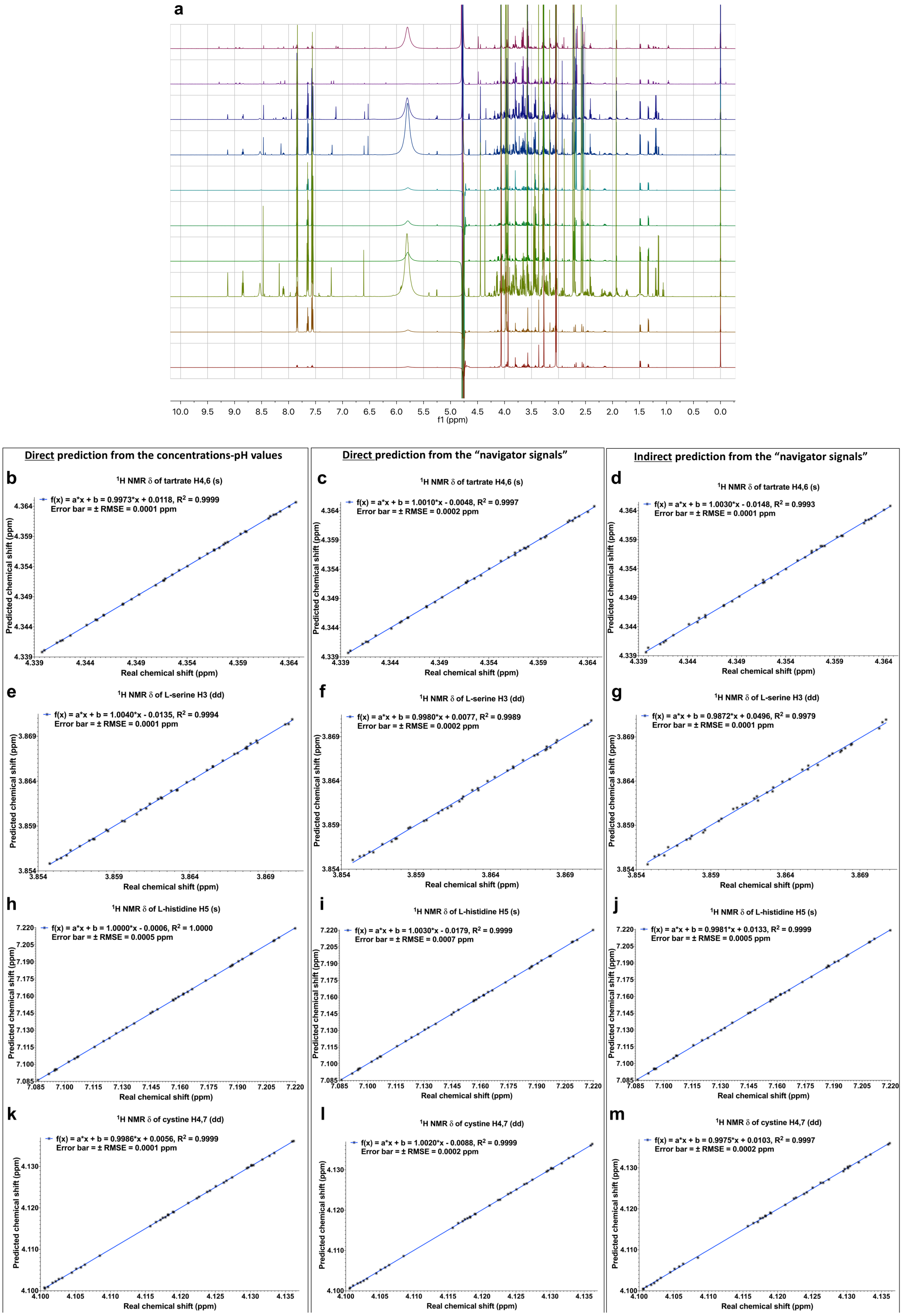
a
f1 (ppm)
10.0 9.5 9.0 8.5 8.0 7.5 7.0 6.5 6.0 5.5 5.0 4.5 4.0 3.5 3.0 2.5 2.0 1.5 1.0 0.5 0.0
Direct prediction from the concentrations-pH values
Direct prediction from the "navigator signals"
Indirect prediction from the "navigator signals"
b
1H NMR δ of tartrate H4,6 (s)
f(x) = a*x + b = 0.9973*x + 0.0118, R2 = 0.9999
Error bar = ± RMSE = 0.0001 ppm
c
1H NMR δ of tartrate H4,6 (s)
f(x) = a*x + b = 1.0010*x - 0.0048, R2 = 0.9997
Error bar = ± RMSE = 0.0002 ppm
d
1H NMR δ of tartrate H4,6 (s)
f(x) = a*x + b = 1.0030*x - 0.0148, R2 = 0.9993
Error bar = ± RMSE = 0.0001 ppm
e
1H NMR δ of L-serine H3 (dd)
f(x) = a*x + b = 1.0040*x - 0.0135, R2 = 0.9994
Error bar = ± RMSE = 0.0001 ppm
f
1H NMR δ of L-serine H3 (dd)
f(x) = a*x + b = 0.9980*x + 0.0077, R2 = 0.9989
Error bar = ± RMSE = 0.0002 ppm
g
1H NMR δ of L-serine H3 (dd)
f(x) = a*x + b = 0.9872*x + 0.0496, R2 = 0.9979
Error bar = ± RMSE = 0.0001 ppm
h
1H NMR δ of L-histidine H5 (s)
f(x) = a*x + b = 1.0000*x - 0.0006, R2 = 1.0000
Error bar = ± RMSE = 0.0005 ppm
i
1H NMR δ of L-histidine H5 (s)
f(x) = a*x + b = 1.0030*x - 0.0179, R2 = 0.9999
Error bar = ± RMSE = 0.0007 ppm
j
1H NMR δ of L-histidine H5 (s)
f(x) = a*x + b = 0.9981*x + 0.0133, R2 = 0.9999
Error bar = ± RMSE = 0.0005 ppm
k
1H NMR δ of cystine H4,7 (dd)
f(x) = a*x + b = 0.9986*x + 0.0056, R2 = 0.9999
Error bar = ± RMSE = 0.0001 ppm
l
1H NMR δ of cystine H4,7 (dd)
f(x) = a*x + b = 1.0020*x - 0.0088, R2 = 0.9999
Error bar = ± RMSE = 0.0002 ppm
m
1H NMR δ of cystine H4,7 (dd)
f(x) = a*x + b = 0.9975*x + 0.0103, R2 = 0.9997
Error bar = ± RMSE = 0.0002 ppm
Predicted chemical shift (ppm)
Real chemical shift (ppm)

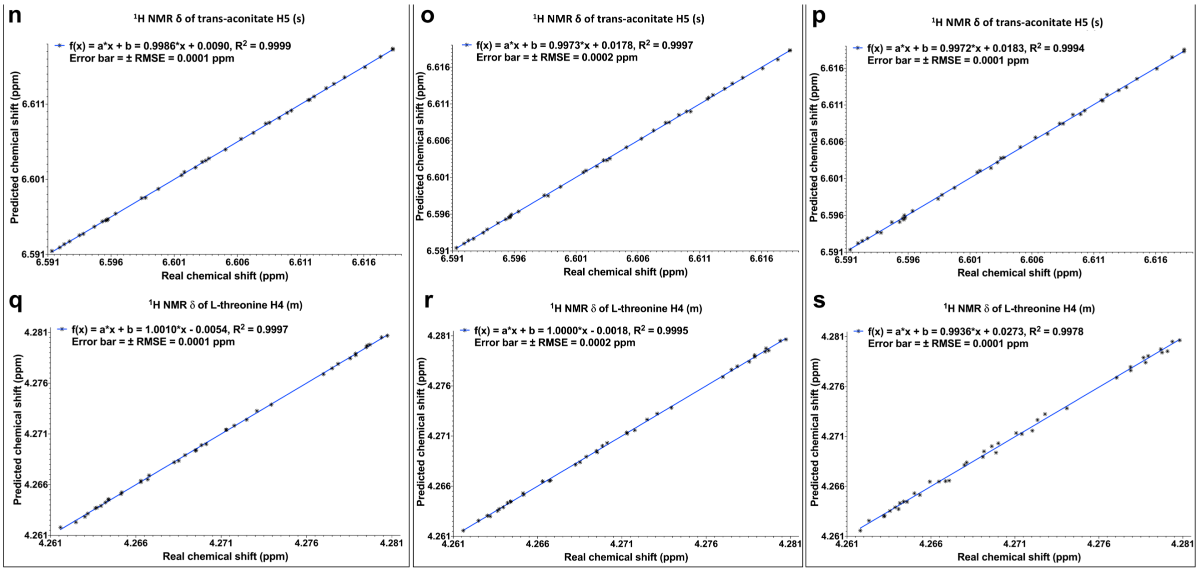

Supplementary Figure 2. Chemical shifts (δ) prediction of 6 selected spins systems of 6 metabolites, tested in 40 randomly prepared artificial urine mixtures. Each mixture consisted of all active metabolites, ions and albumin with concentration values not present in the original artificial urine matrix, but inside the limits of the multidimensional concentration-pH space (Supplementary Table 1). (**a**) The ^{1}H NMR spectra of ten out of the forty random mixtures are shown. Three predicted δ values are obtained for the (**b-d**) tartrate H4,6 protons, (**e-g**) L-serine H3 proton, (**h-j**) L-histidine H5 proton, (**k-m**) cystine H4,7 protons, (**n-p**) trans-aconitate H5 proton and (**q-s**) L-threonine H4 proton, by three different approaches. The first approach (**b,e,h,k,n,q**) was from the metabolites-ions concentration and pH values, the second approach (**c,f,i,l,o,r**) was directly from the navigator signals δ values, and the third one (**d,g,j,m,p,s**) was again from the metabolites-ions concentration and pH values which were previously predicted from the navigator signals δ values. The predicted vs. measured δ values plots (**b-s**) clearly indicate that the δ predictions are very accurate for all 3 approaches. The smallest error (± 0.0002 ppm) was observed for the direct predictions from the metabolites-ions concentration and pH values. The error was slightly higher (± 0.0003 ppm) for the direct predictions from the navigator signals. The indirect predictions from the navigator signals exhibited the highest error (± 0.0004 ppm).

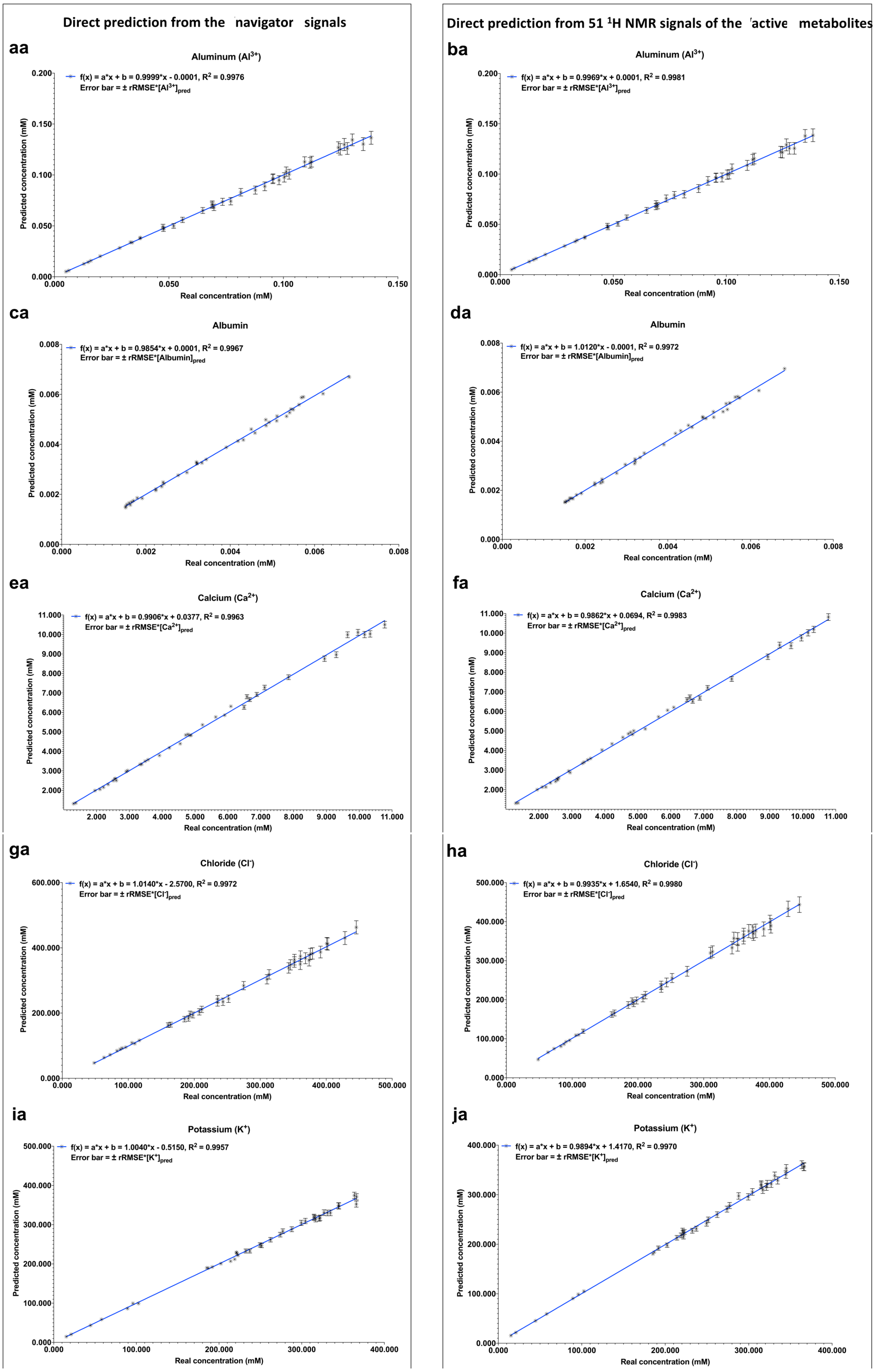
Direct prediction from the 'navigator' signals
Direct prediction from 51 1H NMR signals of the 'active' metabolites
aa
Aluminum (Al3+)
f(x) = a*x + b = 0.9999*x - 0.0001, R2 = 0.9976
Error bar = ± rRMSE*[Al3+]pred
Predicted concentration (mM)
Real concentration (mM)
ba
Aluminum (Al3+)
f(x) = a*x + b = 0.9969*x + 0.0001, R2 = 0.9981
Error bar = ± rRMSE*[Al3+]pred
Predicted concentration (mM)
Real concentration (mM)
ca
Albumin
f(x) = a*x + b = 0.9854*x + 0.0001, R2 = 0.9967
Error bar = ± rRMSE*[Albumin]pred
Predicted concentration (mM)
Real concentration (mM)
da
Albumin
f(x) = a*x + b = 1.0120*x - 0.0001, R2 = 0.9972
Error bar = ± rRMSE*[Albumin]pred
Predicted concentration (mM)
Real concentration (mM)
ea
Calcium (Ca2+)
f(x) = a*x + b = 0.9906*x + 0.0377, R2 = 0.9963
Error bar = ± rRMSE*[Ca2+]pred
Predicted concentration (mM)
Real concentration (mM)
fa
Calcium (Ca2+)
f(x) = a*x + b = 0.9862*x + 0.0694, R2 = 0.9983
Error bar = ± rRMSE*[Ca2+]pred
Predicted concentration (mM)
Real concentration (mM)
ga
Chloride (Cl-)
f(x) = a*x + b = 1.0140*x - 2.5700, R2 = 0.9972
Error bar = ± rRMSE*[Cl-]pred
Predicted concentration (mM)
Real concentration (mM)
ha
Chloride (Cl-)
f(x) = a*x + b = 0.9935*x + 1.6540, R2 = 0.9980
Error bar = ± rRMSE*[Cl-]pred
Predicted concentration (mM)
Real concentration (mM)
ia
Potassium (K+)
f(x) = a*x + b = 1.0040*x - 0.5150, R2 = 0.9957
Error bar = ± rRMSE*[K+]pred
Predicted concentration (mM)
Real concentration (mM)
ja
Potassium (K+)
f(x) = a*x + b = 0.9894*x + 1.4170, R2 = 0.9970
Error bar = ± rRMSE*[K+]pred
Predicted concentration (mM)
Real concentration (mM)

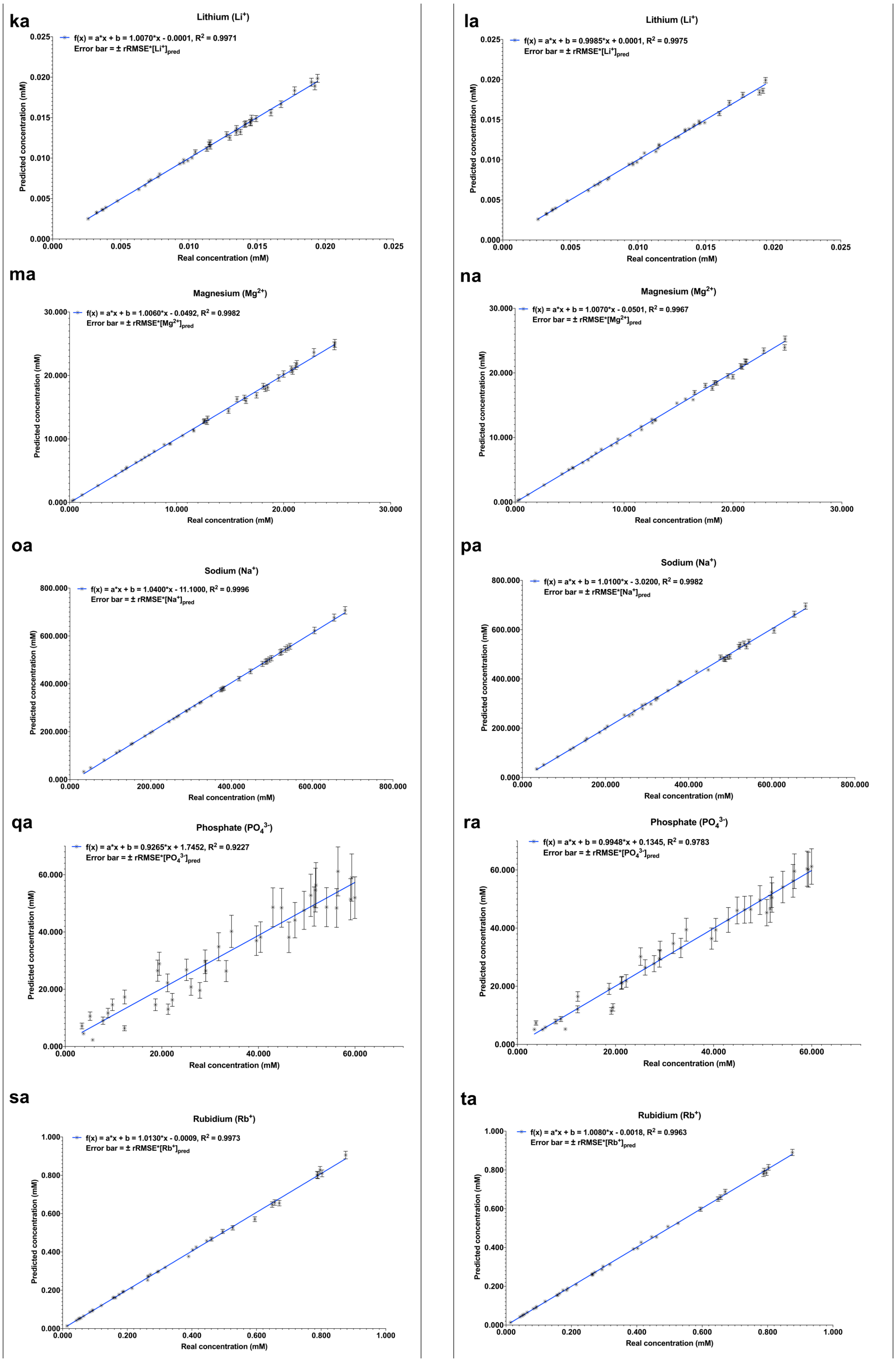
ka
Lithium (Li+)
f(x) = a*x + b = 1.0070*x - 0.0001, R2 = 0.9971
Error bar = ± rRMSE*[Li+]pred
Predicted concentration (mM)
Real concentration (mM)
la
Lithium (Li+)
f(x) = a*x + b = 0.9985*x + 0.0001, R2 = 0.9975
Error bar = ± rRMSE*[Li+]pred
Predicted concentration (mM)
Real concentration (mM)
ma
Magnesium (Mg2+)
f(x) = a*x + b = 1.0060*x - 0.0492, R2 = 0.9982
Error bar = ± rRMSE*[Mg2+]pred
Predicted concentration (mM)
Real concentration (mM)
na
Magnesium (Mg2+)
f(x) = a*x + b = 1.0070*x - 0.0501, R2 = 0.9967
Error bar = ± rRMSE*[Mg2+]pred
Predicted concentration (mM)
Real concentration (mM)
oa
Sodium (Na+)
f(x) = a*x + b = 1.0400*x - 11.1000, R2 = 0.9996
Error bar = ± rRMSE*[Na+]pred
Predicted concentration (mM)
Real concentration (mM)
pa
Sodium (Na+)
f(x) = a*x + b = 1.0100*x - 3.0200, R2 = 0.9982
Error bar = ± rRMSE*[Na+]pred
Predicted concentration (mM)
Real concentration (mM)
qa
Phosphate (PO43-)
f(x) = a*x + b = 0.9265*x + 1.7452, R2 = 0.9227
Error bar = ± rRMSE*[PO43-]pred
Predicted concentration (mM)
Real concentration (mM)
ra
Phosphate (PO43-)
f(x) = a*x + b = 0.9948*x + 0.1345, R2 = 0.9783
Error bar = ± rRMSE*[PO43-]pred
Predicted concentration (mM)
Real concentration (mM)
sa
Rubidium (Rb+)
f(x) = a*x + b = 1.0130*x - 0.0009, R2 = 0.9973
Error bar = ± rRMSE*[Rb+]pred
Predicted concentration (mM)
Real concentration (mM)
ta
Rubidium (Rb+)
f(x) = a*x + b = 1.0080*x - 0.0018, R2 = 0.9963
Error bar = ± rRMSE*[Rb+]pred
Predicted concentration (mM)
Real concentration (mM)

ua

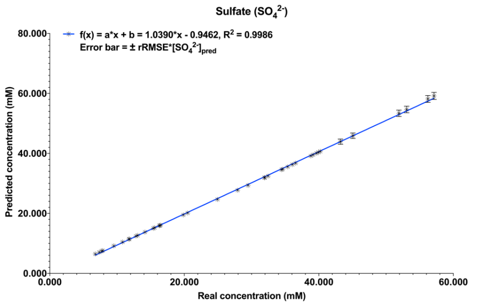

va

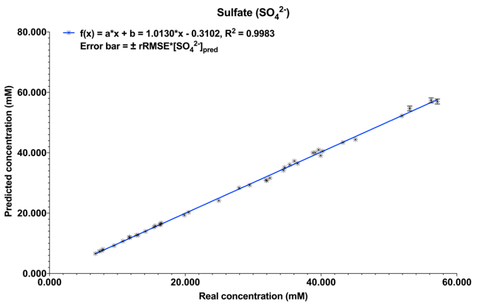

wa

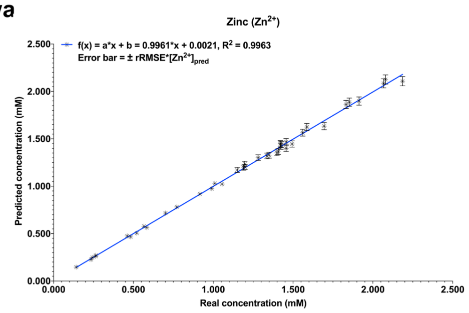

xa

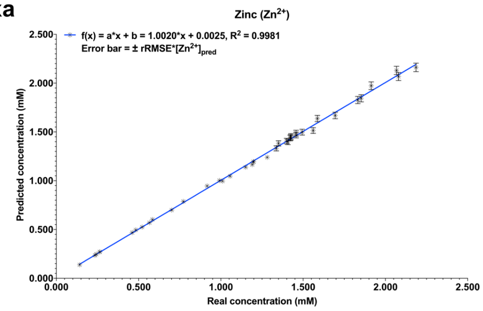

ya

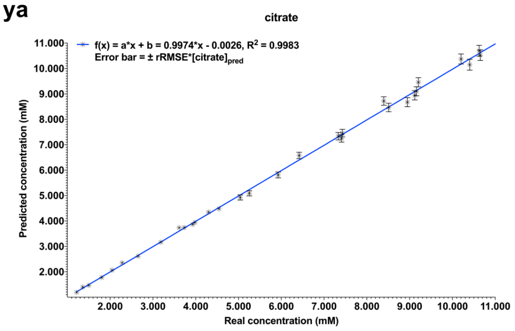

za

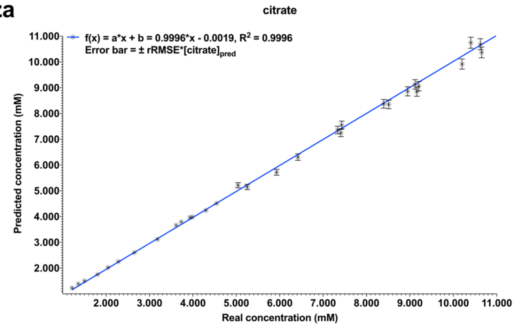

ab

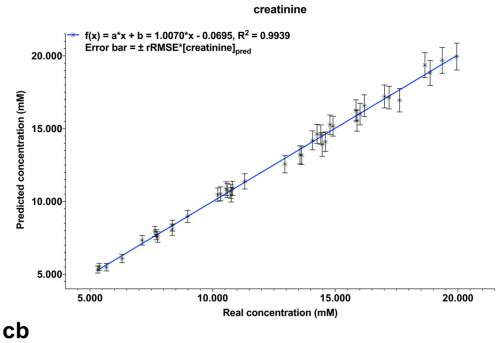

bb

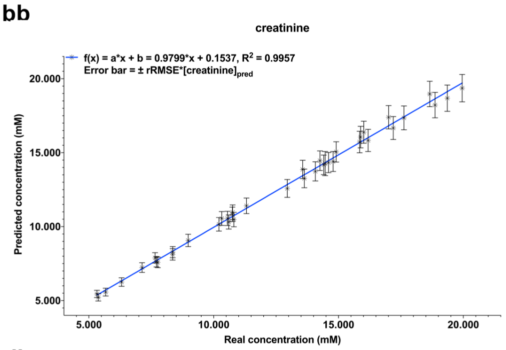

cb

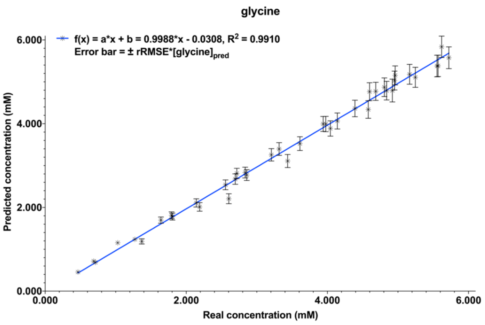

db

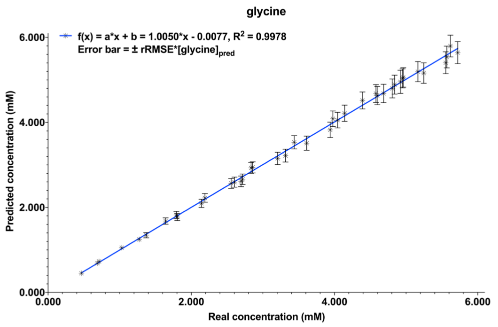

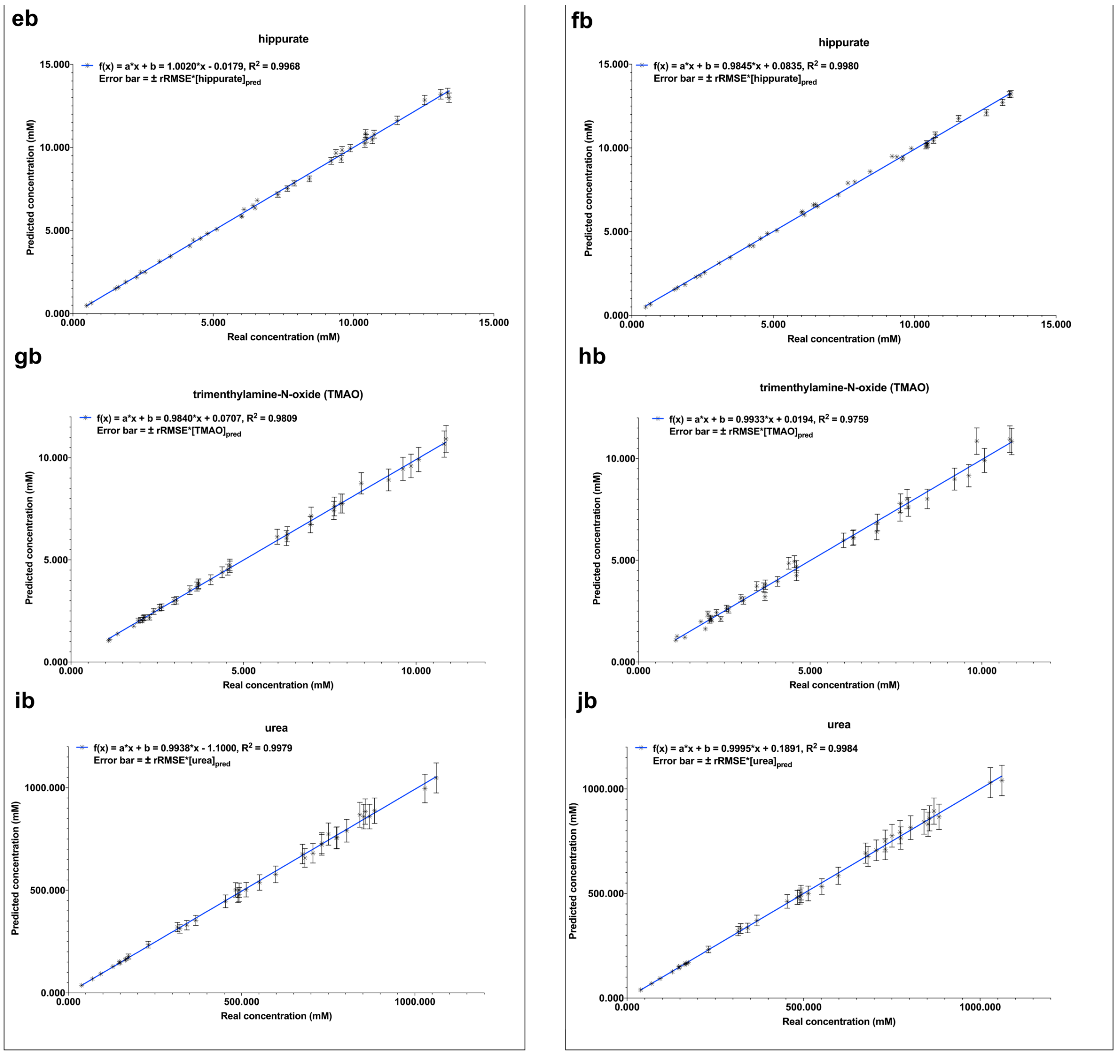

Supplementary Figure 3. Predicted concentration values for 11 ions (**aa-xa**) and 6 of the most active metabolites (**ya-jb**) vs. real concentration values in the test set of 40 randomly prepared artificial urine samples. Each of the 40 randomly prepared artificial urine mixtures consisted of the active metabolites-ions-albumin with concentration values not present in the original artificial urine matrix, but inside the limits of the concentration-pH multidimensional space (Supplementary Table 1). The concentrations are directly predicted via (left column) the chemical shifts values of the navigator signals and (right column) via selected 51 ^{1}H NMR signals of the active metabolites. All concentration values of ions, albumin and metabolites are accurately predicted by both approaches, exhibiting almost the same prediction errors (R^2 values indicate very accurate concentration predictions), whereas phosphate ions are better predicted by the 51 ^{1}H NMR signals chemical shifts. This is attributed to the fact that all samples contain a high concentration of phosphate buffer (see Methods section about the preparation of urine NMR samples procedure) so the relative differences in phosphate concentrations among the samples are small.

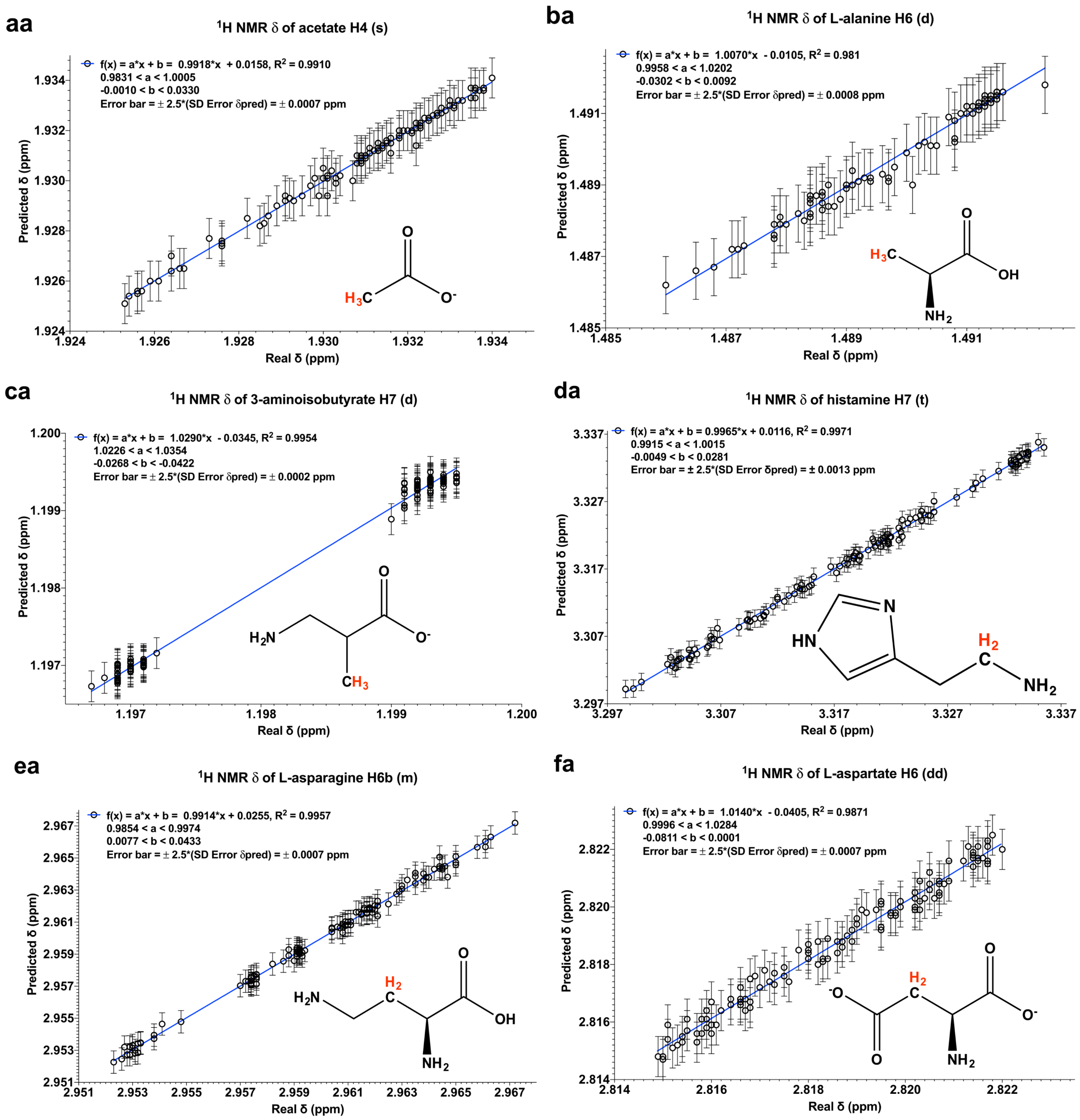
aa
1H NMR δ of acetate H4 (s)
f(x) = a*x + b = 0.9918*x + 0.0158, R2 = 0.9910
0.9831 < a < 1.0005
-0.0010 < b < 0.0330
Error bar = ± 2.5*(SD Error δpred) = ± 0.0007 ppm
Predicted δ (ppm)
Real δ (ppm)
ba
1H NMR δ of L-alanine H6 (d)
f(x) = a*x + b = 1.0070*x - 0.0105, R2 = 0.981
0.9958 < a < 1.0202
-0.0302 < b < 0.0092
Error bar = ± 2.5*(SD Error δpred) = ± 0.0008 ppm
Predicted δ (ppm)
Real δ (ppm)
ca
1H NMR δ of 3-aminoisobutyrate H7 (d)
f(x) = a*x + b = 1.0290*x - 0.0345, R2 = 0.9954
1.0226 < a < 1.0354
-0.0268 < b < -0.0422
Error bar = ± 2.5*(SD Error δpred) = ± 0.0002 ppm
Predicted δ (ppm)
Real δ (ppm)
da
1H NMR δ of histamine H7 (t)
f(x) = a*x + b = 0.9965*x + 0.0116, R2 = 0.9971
0.9915 < a < 1.0015
-0.0049 < b < 0.0281
Error bar = ± 2.5*(SD Error δpred) = ± 0.0013 ppm
Predicted δ (ppm)
Real δ (ppm)
ea
1H NMR δ of L-asparagine H6b (m)
f(x) = a*x + b = 0.9914*x + 0.0255, R2 = 0.9957
0.9854 < a < 0.9974
0.0077 < b < 0.0433
Error bar = ± 2.5*(SD Error δpred) = ± 0.0007 ppm
Predicted δ (ppm)
Real δ (ppm)
fa
1H NMR δ of L-aspartate H6 (dd)
f(x) = a*x + b = 1.0140*x - 0.0405, R2 = 0.9871
0.9996 < a < 1.0284
-0.0811 < b < 0.0001
Error bar = ± 2.5*(SD Error δpred) = ± 0.0007 ppm
Predicted δ (ppm)
Real δ (ppm)

ga

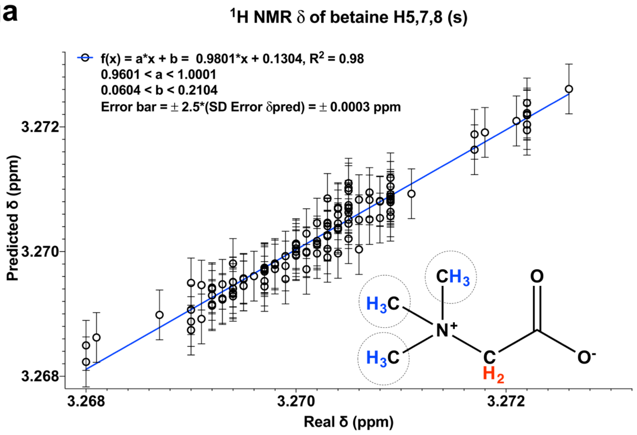

ha

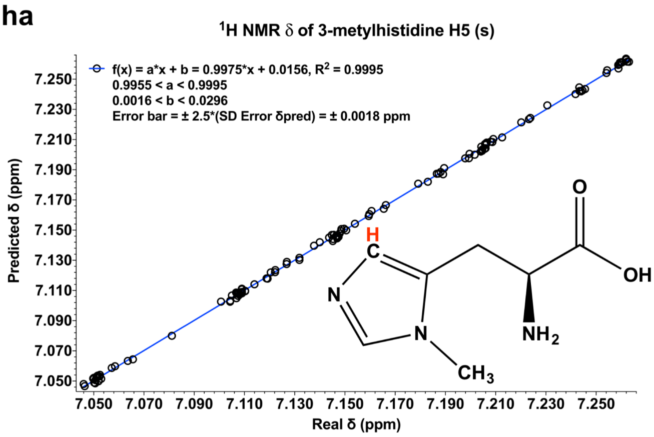

ia

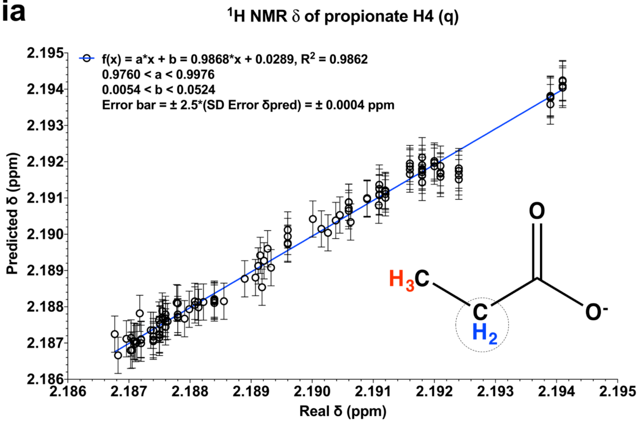

ja

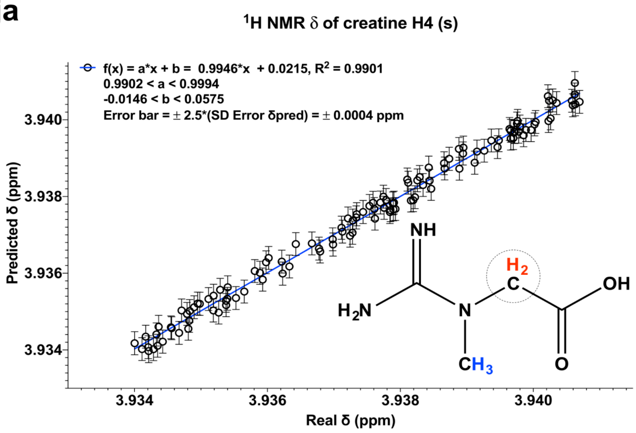

ka

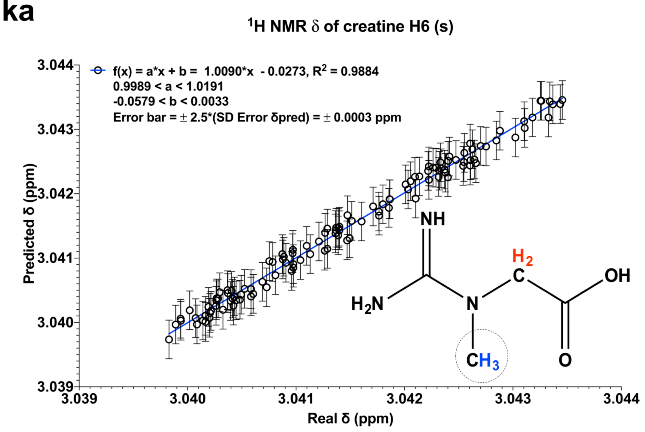

la

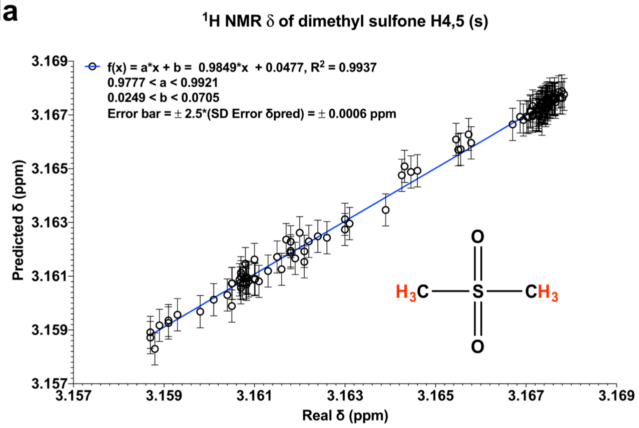

ma

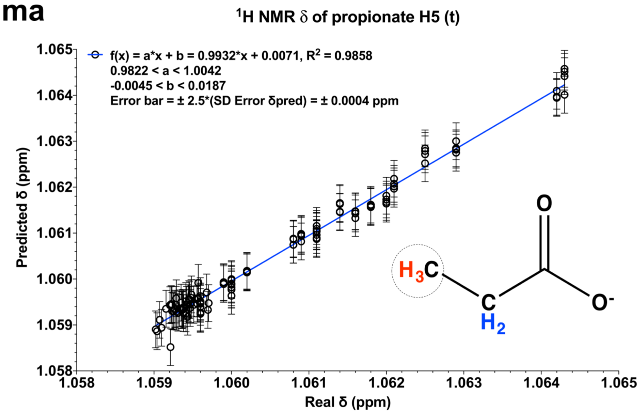

na

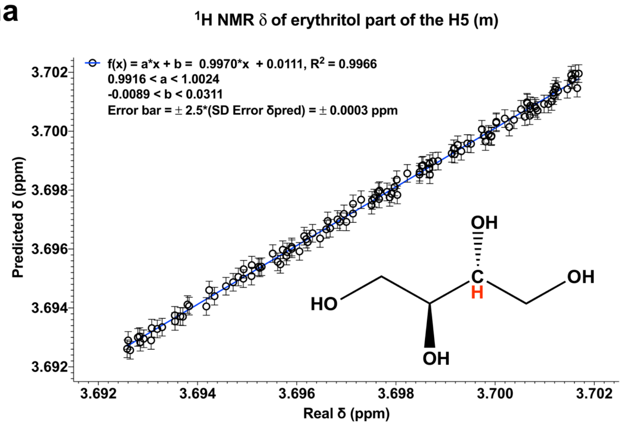

oa

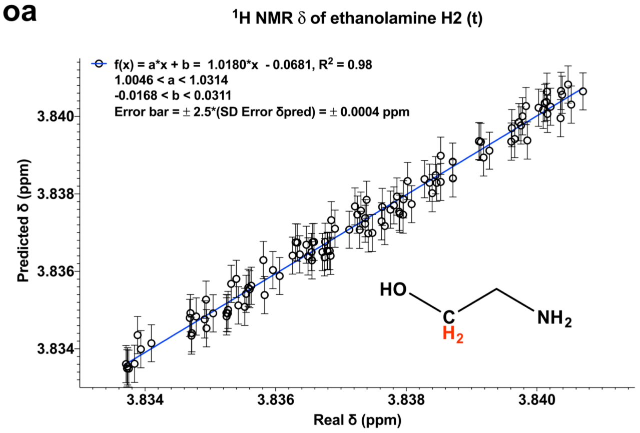

pa

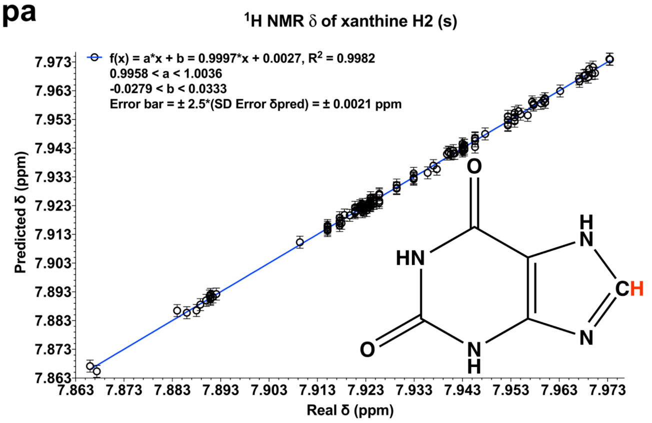

qa

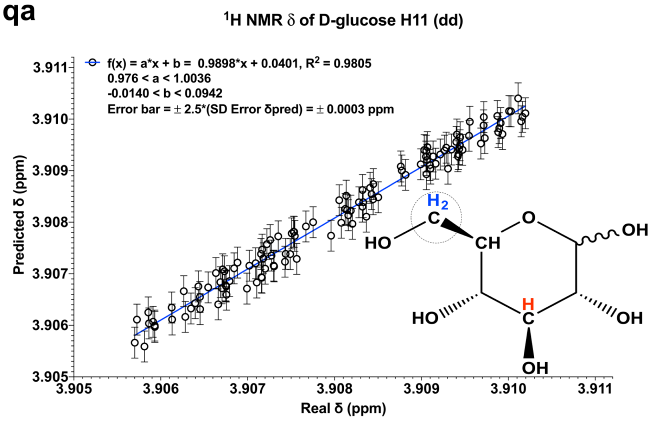

ra

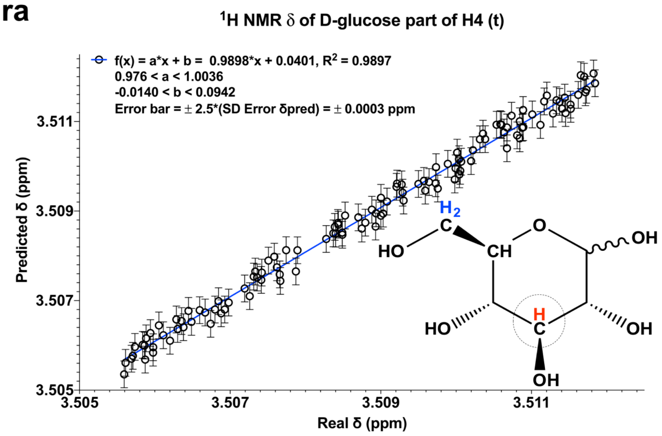

sa

^{1}H NMR δ of L-glutamine H7a (m)

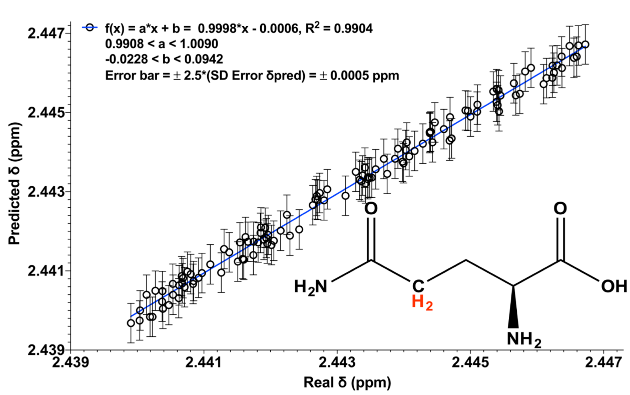

ta

^{1}H NMR δ of L-glutamine H7b (m)

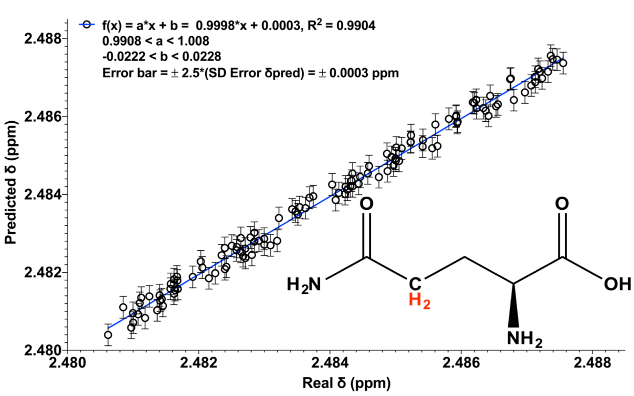

ua

^{1}H NMR δ of L-glutamate H6b (m)

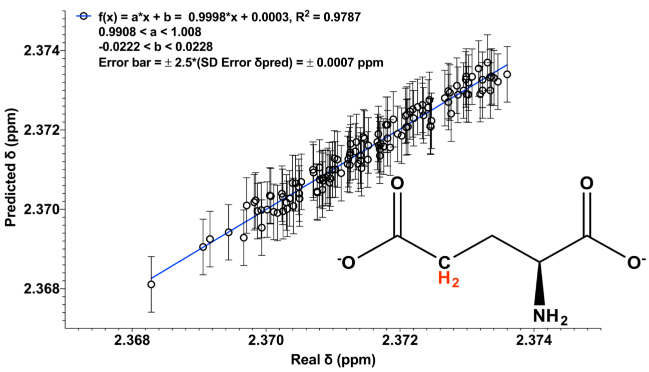

va

^{1}H NMR δ of L-glutamate H6a (m)

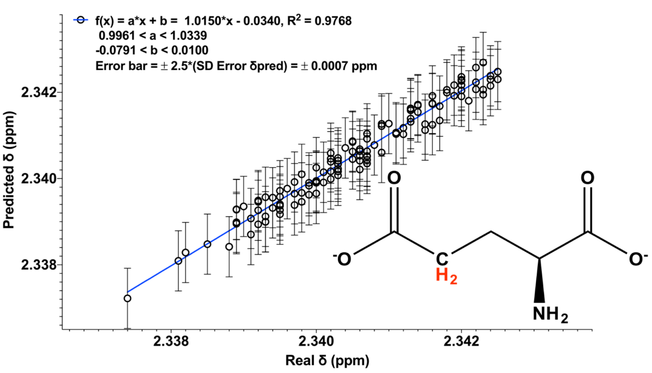

wa

^{1}H NMR δ of glycerol H3,5a (m)

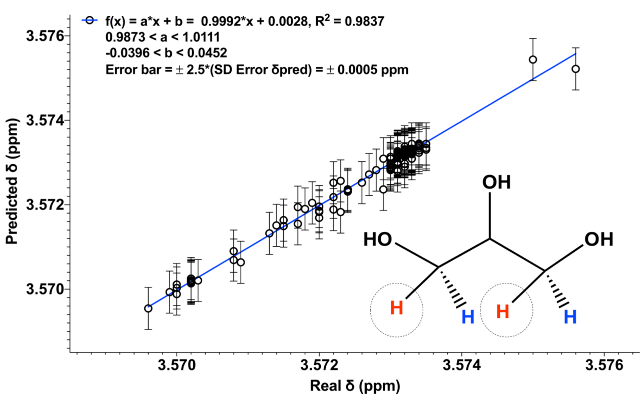

xa

^{1}H NMR δ of glycerol H3,5b (m)

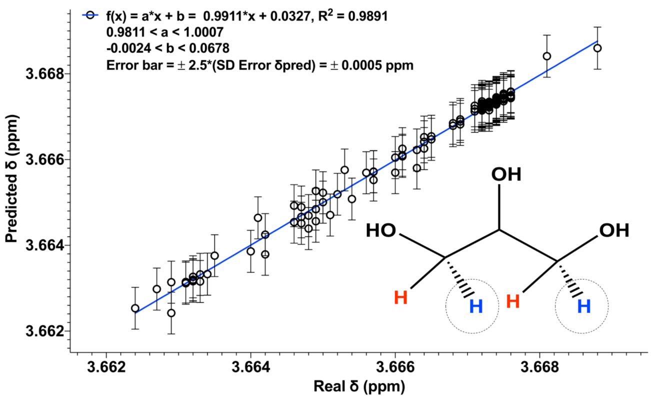

ya

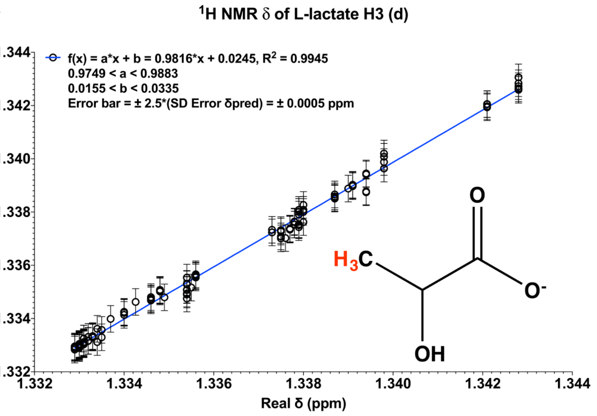

za

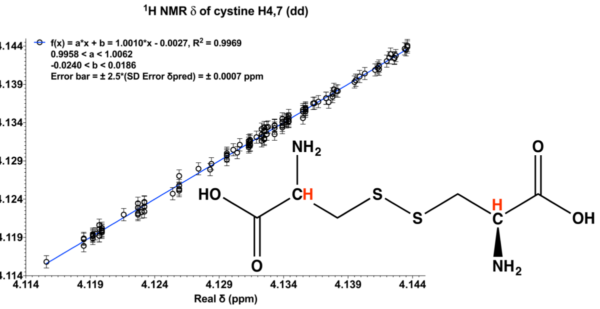

ab

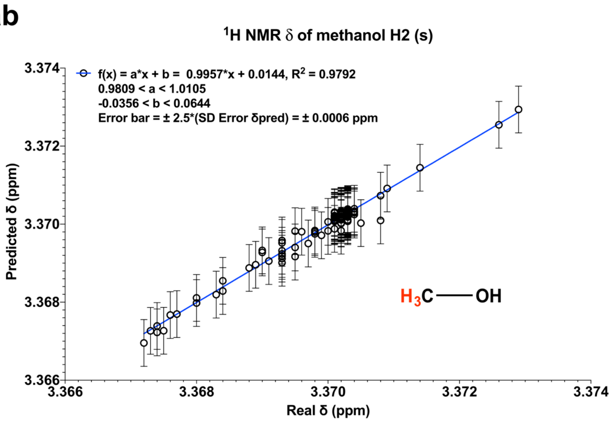

bb

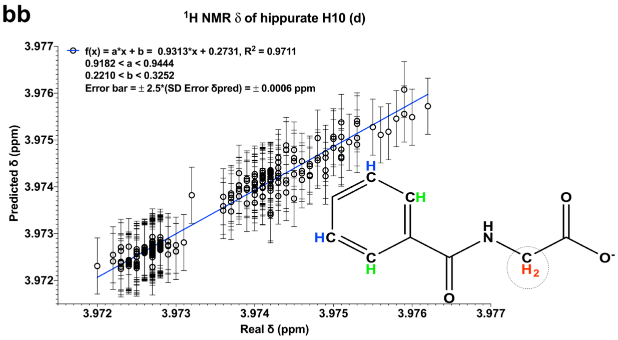

db

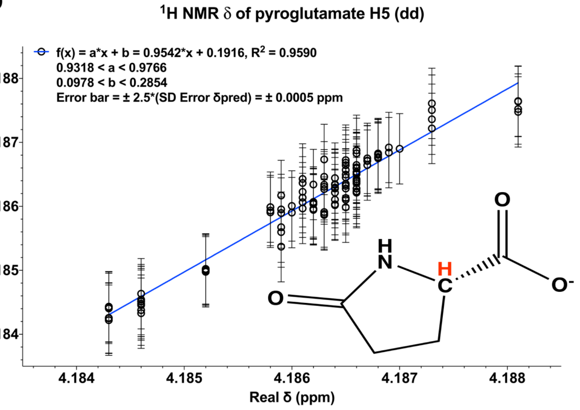

cb

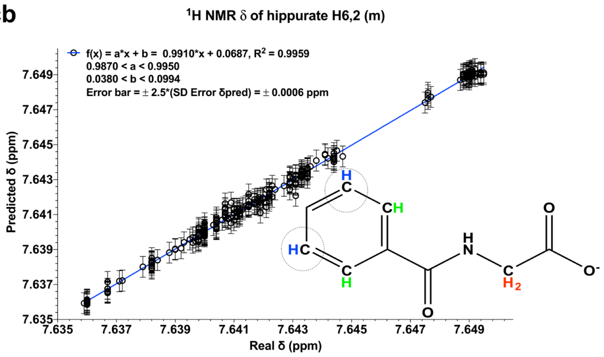

eb

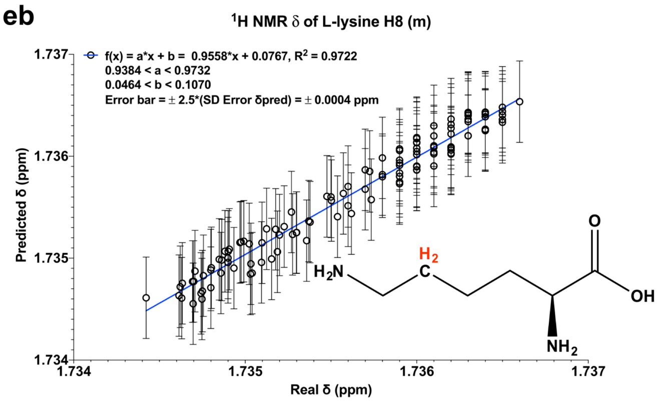

1H NMR δ of L-lysine H8 (m)
f(x) = a*x + b = 0.9558*x + 0.0767, R2 = 0.9722
0.9384 < a < 0.9732
0.0464 < b < 0.1070
Error bar = ± 2.5*(SD Error δpred) = ± 0.0004 ppm
Predicted δ (ppm)
Real δ (ppm)
1.734
1.735
1.736
1.737
H2N
H2
C
O
OH
NH2

fb

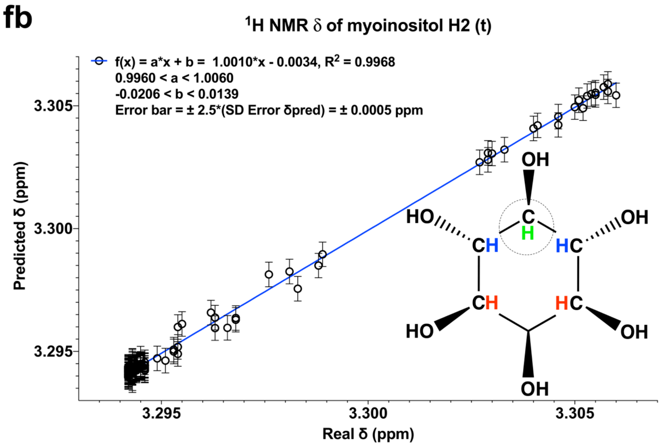

1H NMR δ of myoinositol H2 (t)
f(x) = a*x + b = 1.0010*x - 0.0034, R2 = 0.9968
0.9960 < a < 1.0060
-0.0206 < b < 0.0139
Error bar = ± 2.5*(SD Error δpred) = ± 0.0005 ppm
Predicted δ (ppm)
Real δ (ppm)
3.295
3.300
3.305
OH
HO
OH
C
H
CH
HC
CH
HC
HO
OH
OH

gb

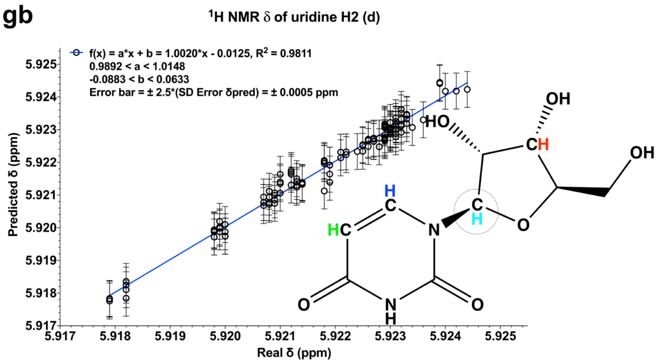

1H NMR δ of uridine H2 (d)
f(x) = a*x + b = 1.0020*x - 0.0125, R2 = 0.9811
0.9892 < a < 1.0148
-0.0883 < b < 0.0633
Error bar = ± 2.5*(SD Error δpred) = ± 0.0005 ppm
Predicted δ (ppm)
Real δ (ppm)
5.917
5.918
5.919
5.920
5.921
5.922
5.923
5.924
5.925
OH
HO
CH
OH
H
C
HC
N
C
H
O
O
N
H
O

hb

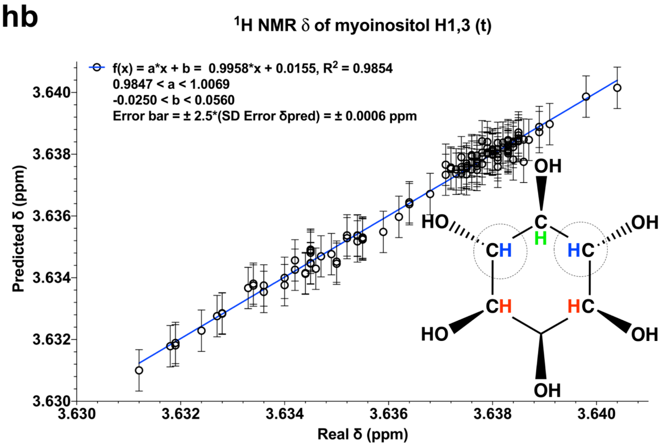

1H NMR δ of myoinositol H1,3 (t)
f(x) = a*x + b = 0.9958*x + 0.0155, R2 = 0.9854
0.9847 < a < 1.0069
-0.0250 < b < 0.0560
Error bar = ± 2.5*(SD Error δpred) = ± 0.0006 ppm
Predicted δ (ppm)
Real δ (ppm)
3.630
3.632
3.634
3.636
3.638
3.640
OH
HO
C
H
OH
CH
HC
CH
HC
HO
OH
OH

ib

^{1}H NMR δ of propylene glycol H5 (d)

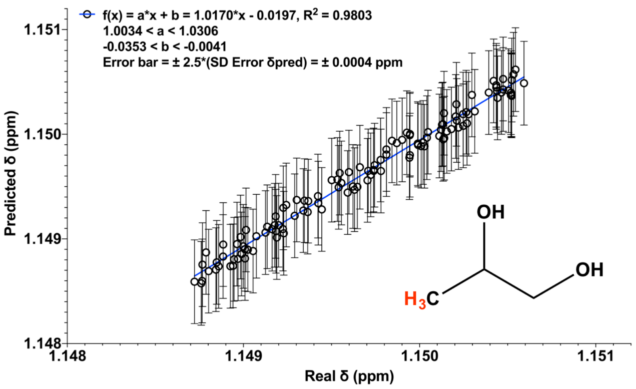

jb

^{1}H NMR δ of 3-hydroxybutyrate H3 (d)

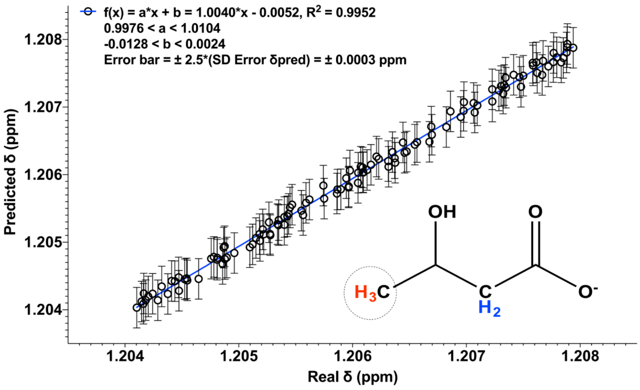

kb

^{1}H NMR δ of 3-hydroxybutyrate H4a (m)

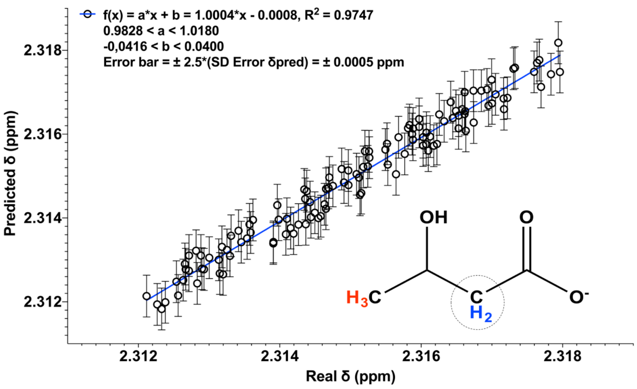

lb

^{1}H NMR δ of acetone H3,4 (s)

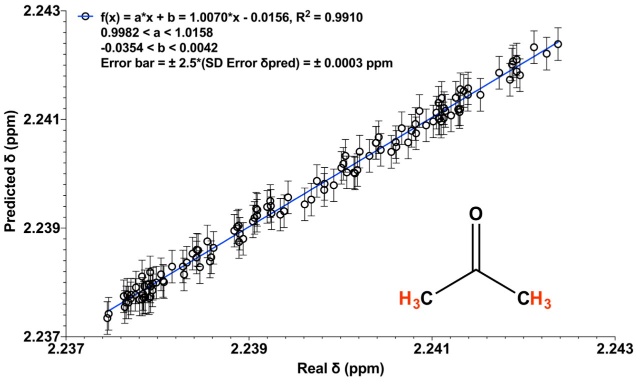

mb

^{1}H NMR δ of N,N-dimethylglycine H6,7 (s)

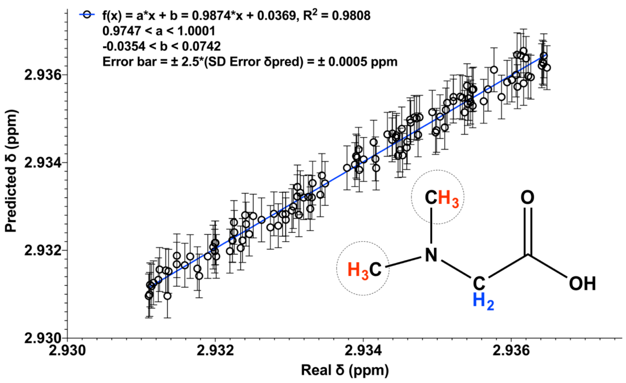

nb

^{1}H NMR δ of N,N-dimethylglycine H4 (s)

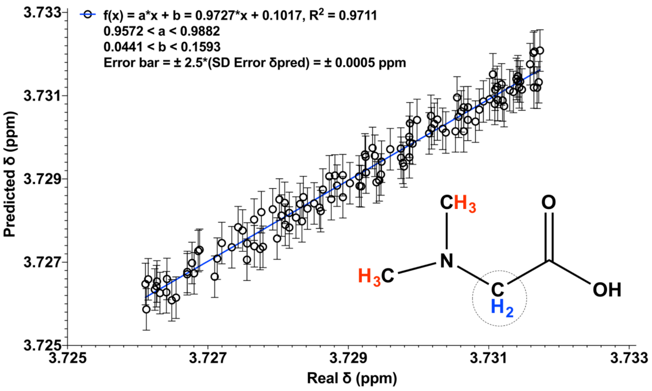

ob

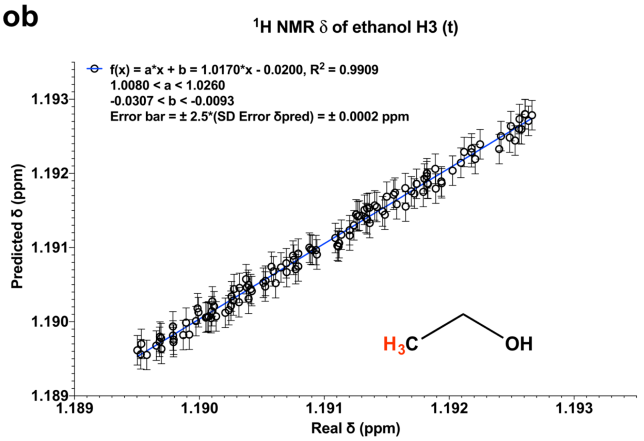

pb

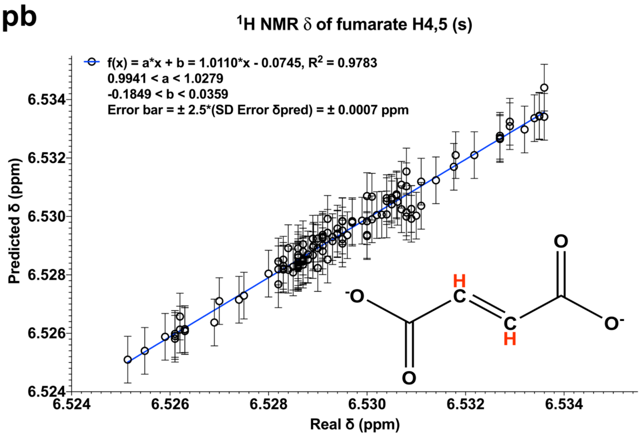

qb

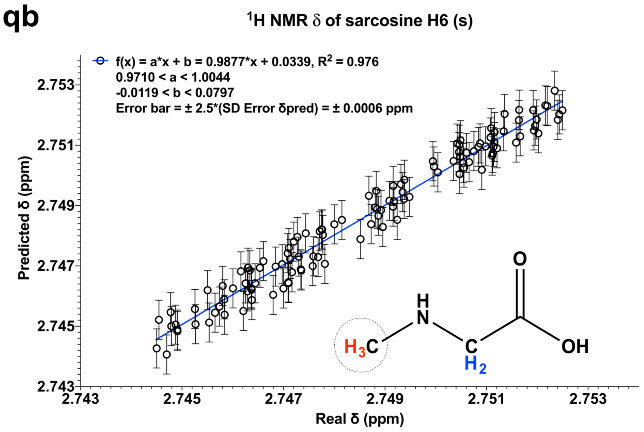

rb

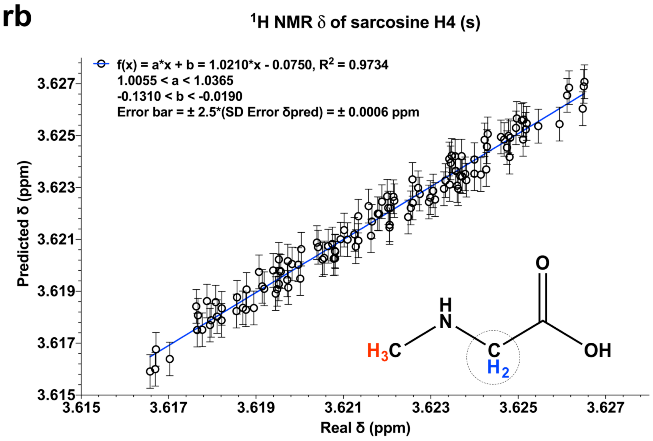

sb

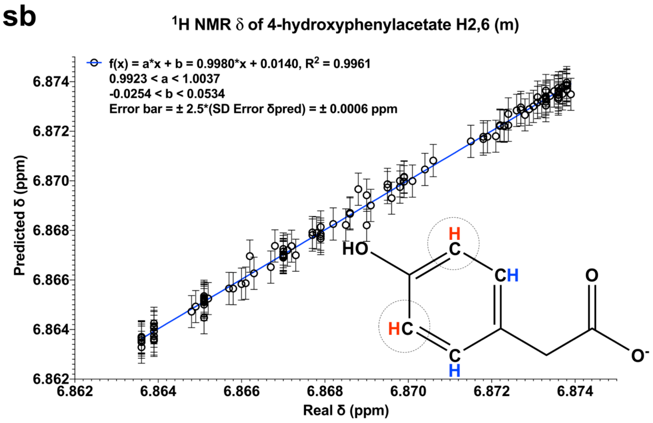

tb

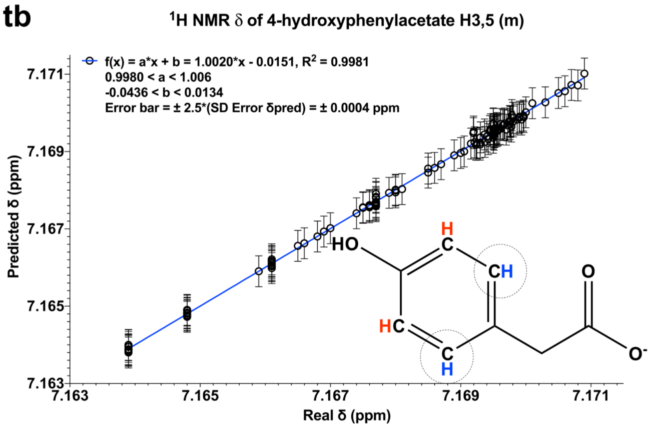

ub

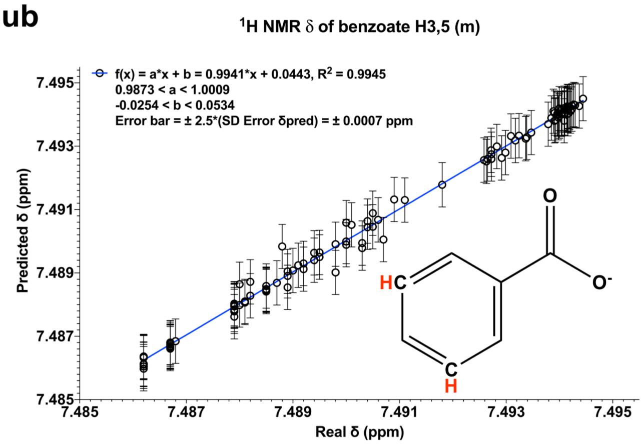

vb

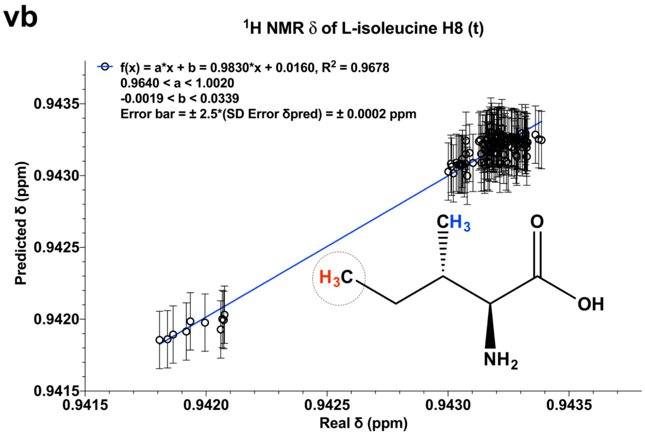

wb

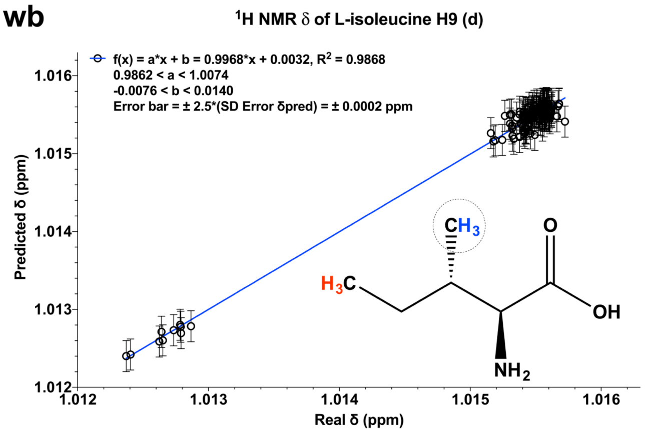

xb

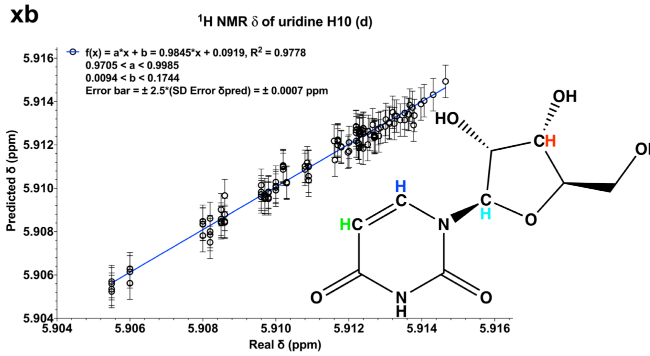

yb

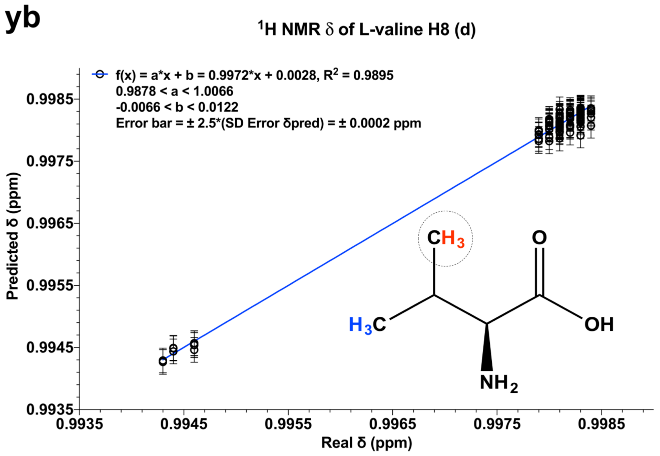

zb

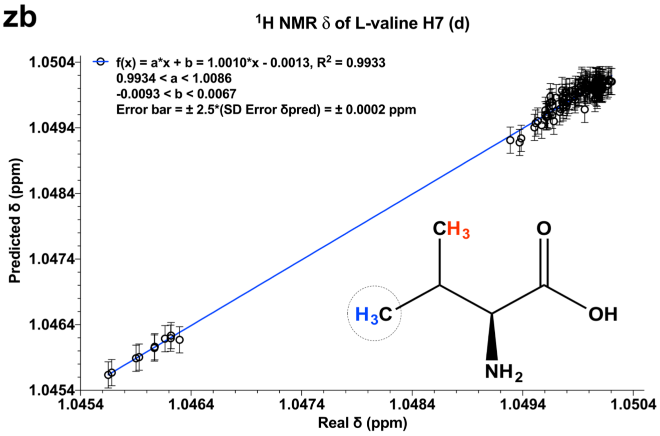

ac

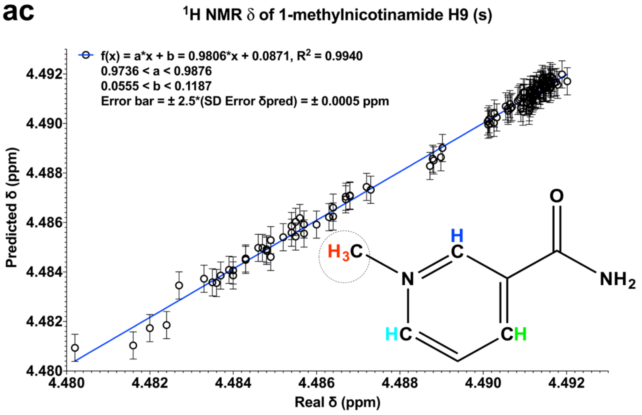

bc

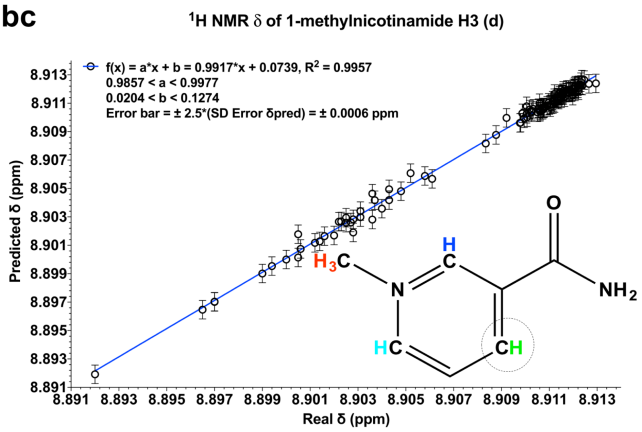

cc

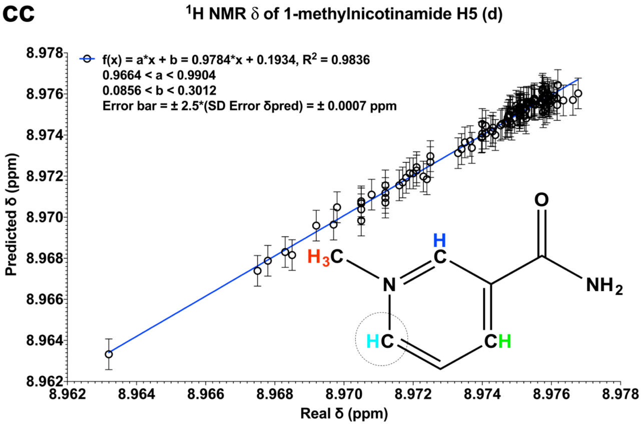

dc

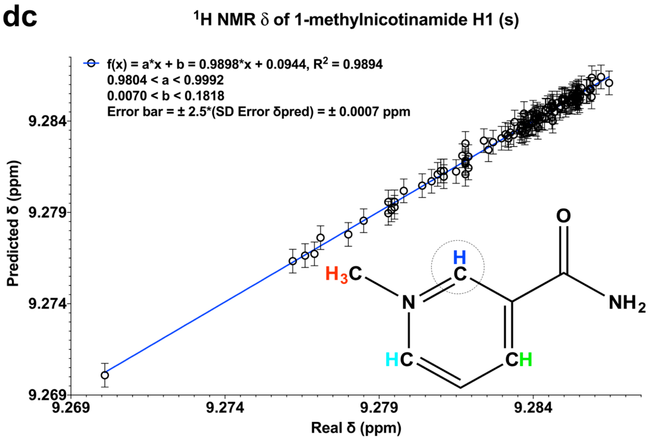

ec

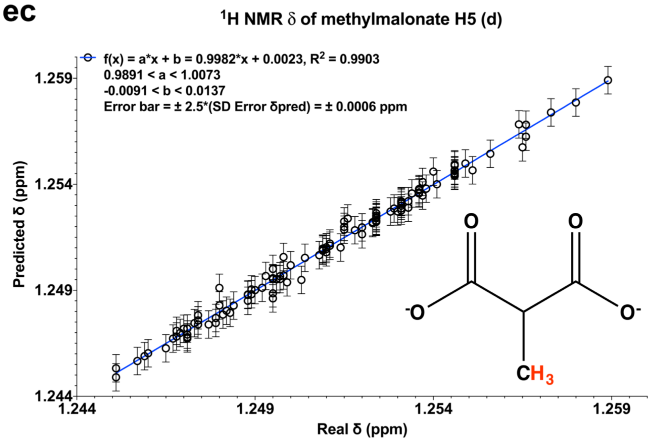

fc

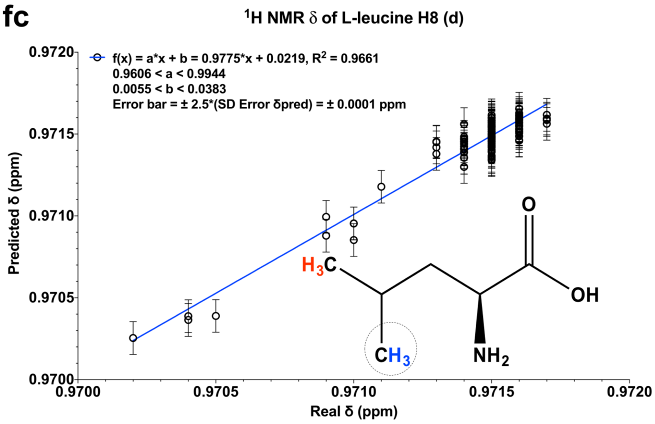

gc

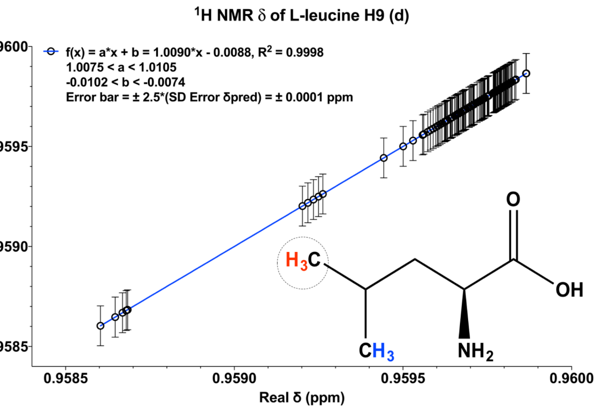

hc

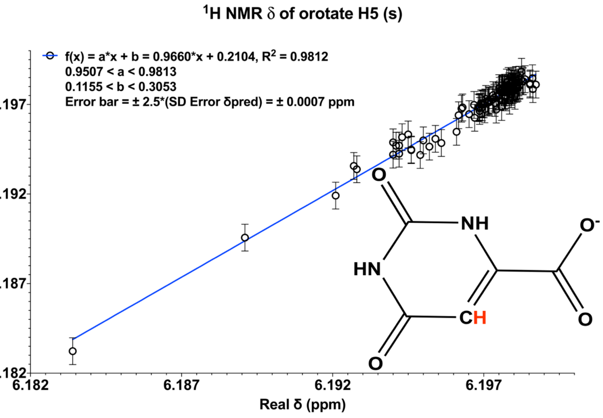

ic

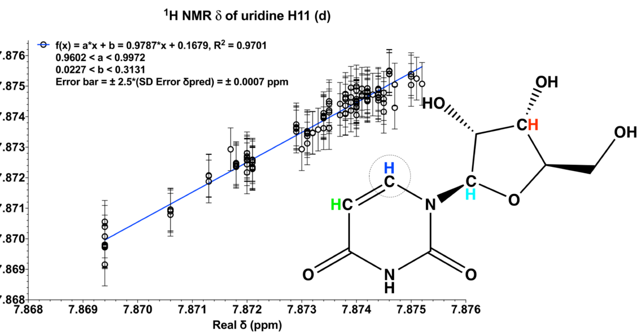

jc

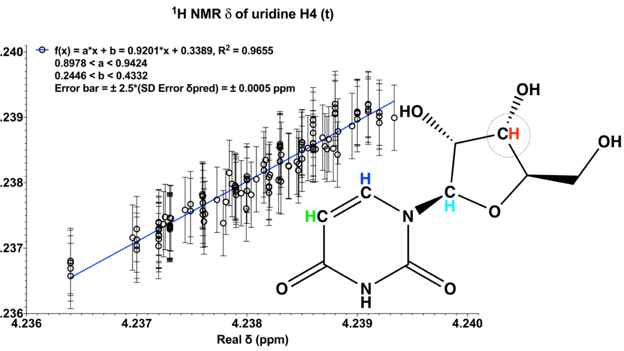

kc

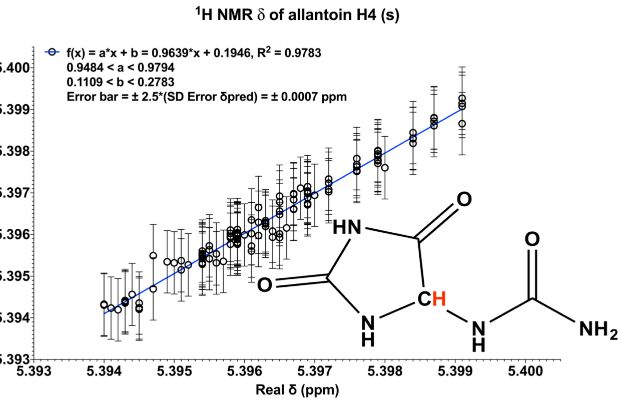

lc

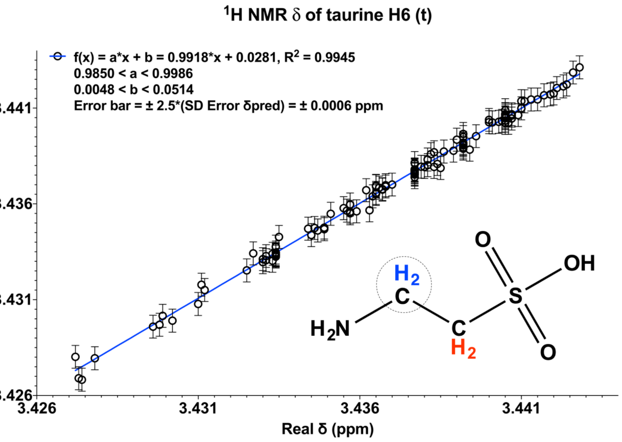

mc

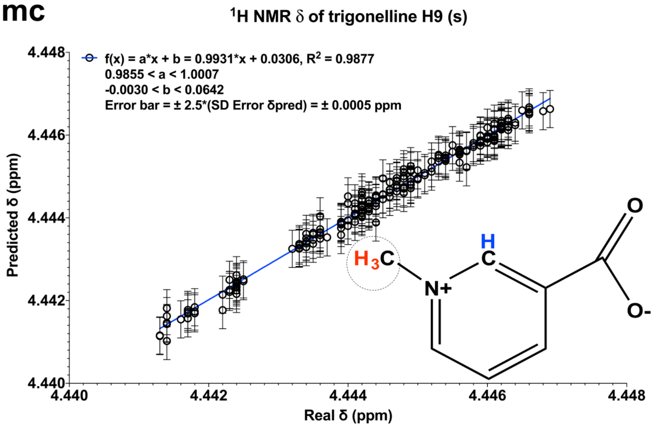

nc

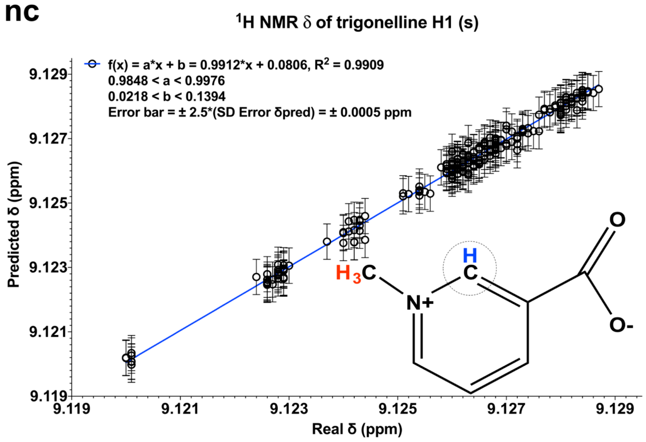

oc

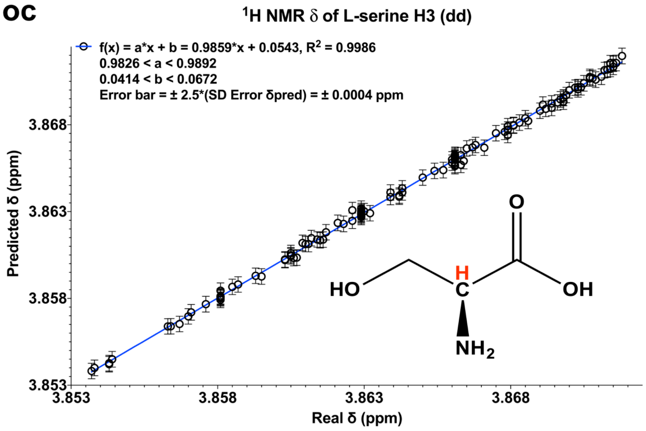

pc

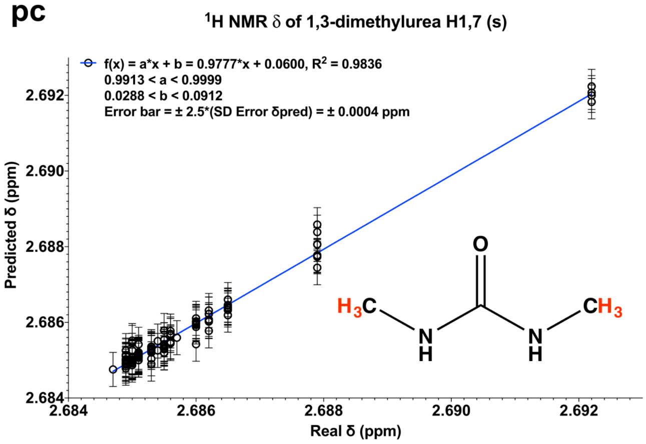

qc

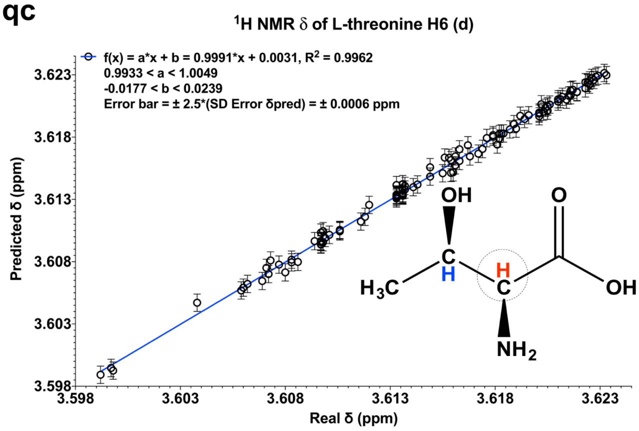

rc

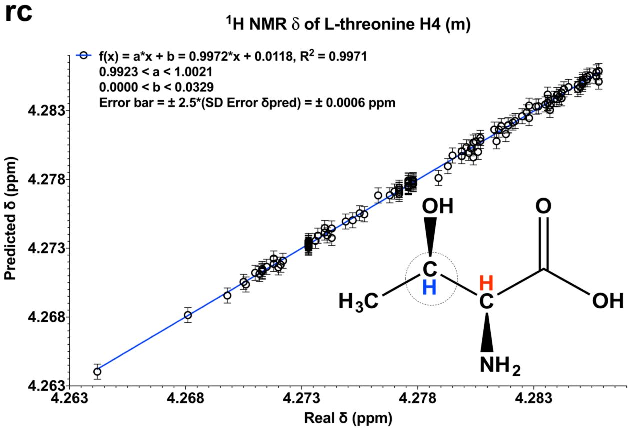

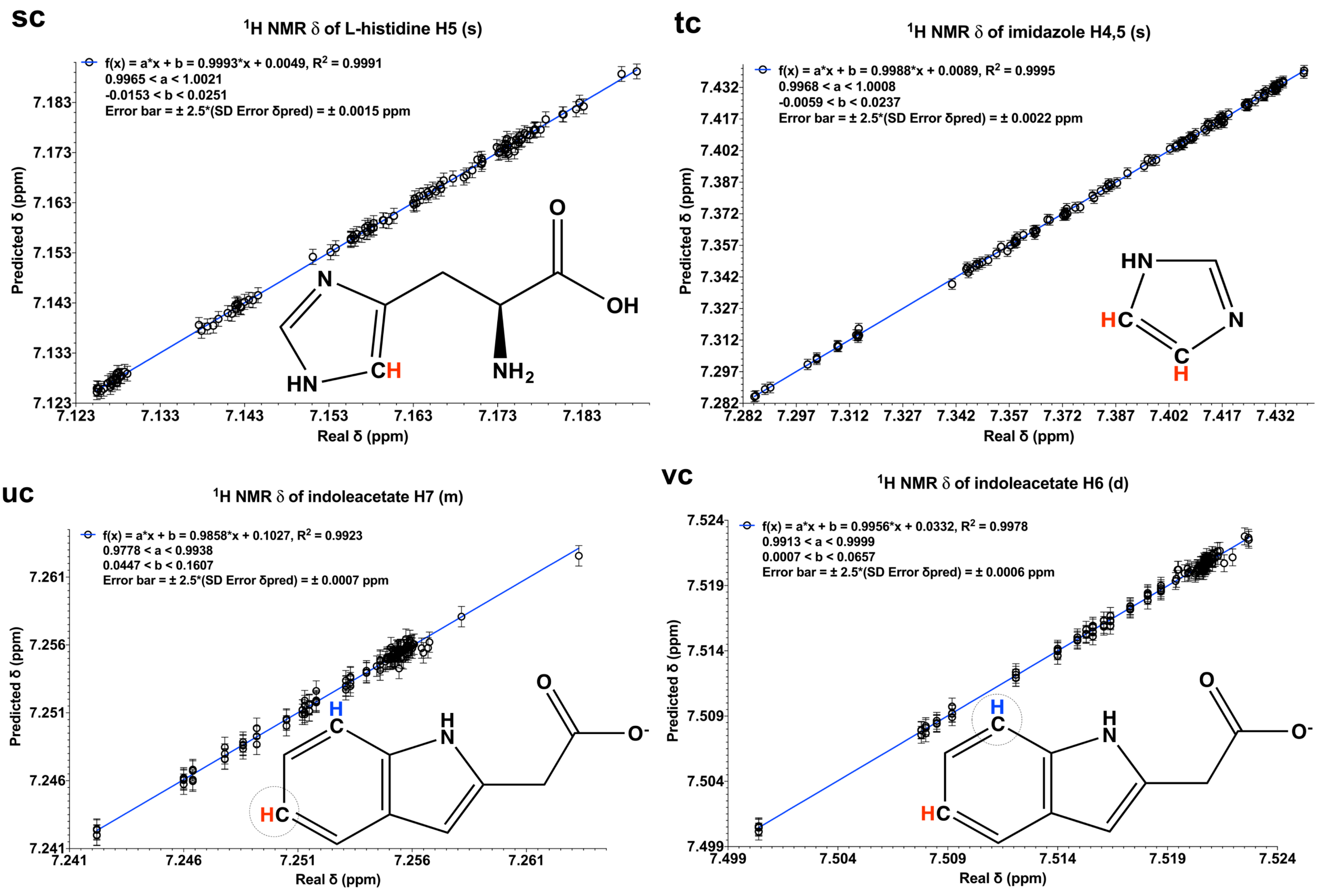

Supplementary Figure 4. Prediction of chemical shifts (δ) for additional 74 spin systems of 50 metabolites in 120 urine NMR spectra. Colored fonts and dashed circles indicate the proton spin system for which δ is predicted. The prediction errors are within 1.5 linewidth for 67 out of 74 spin systems. The cases of (**ha**) 3 methylhistidine, (**pa**) xanthine, (**sc**) histidine, (**tc**) imidazole signals and (**da**) histamine aliphatic signals constitute exceptions, as their predicted shifts are within 3, 4.5, 4.2, 3.6 and 2.6 linewidths, respectively, but their δ range is extremely large (see Supplementary Fig. 4ha,pa,sc,tc,da and Supplementary Table 1), so their relative accuracy is also very high.

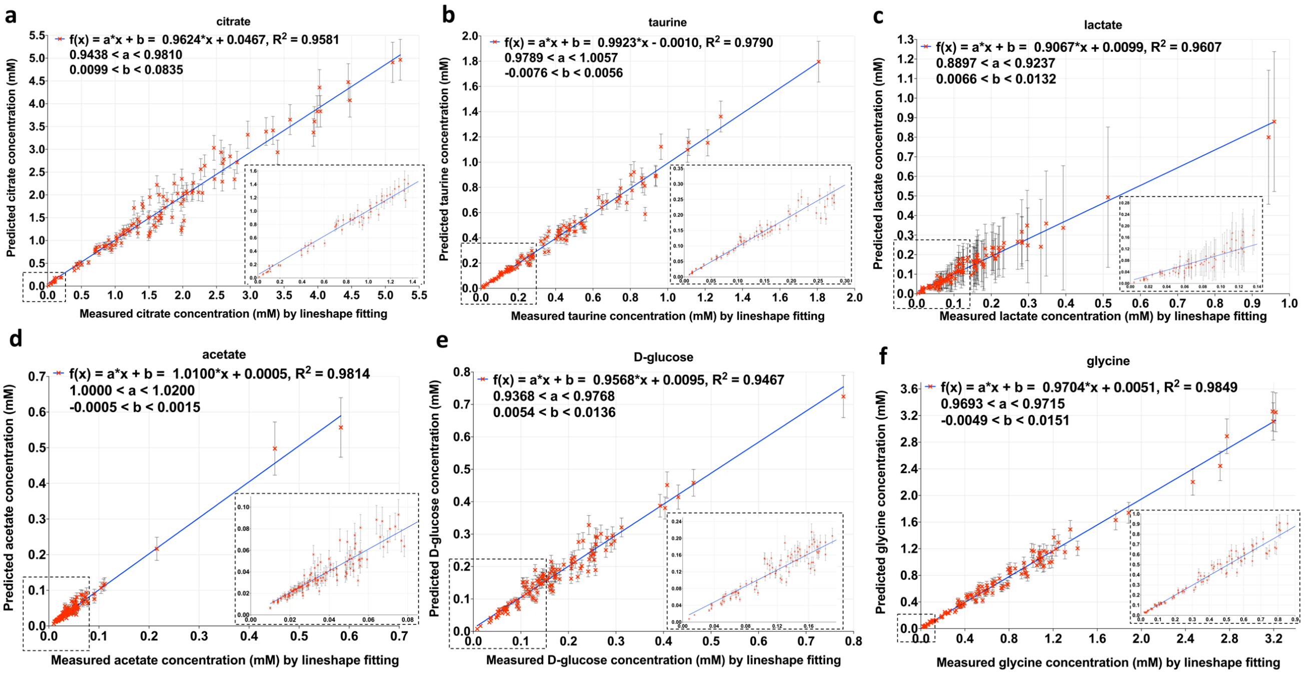

Supplementary Figure 5. Predicted vs. measured concentrations of additional 6 among the most active metabolites in 120 real urine samples. (**a**) citrate, (**b**) taurine, (**c**) lactate, (**d**) acetate, (**e**) D-glucose and (**f**) glycine.

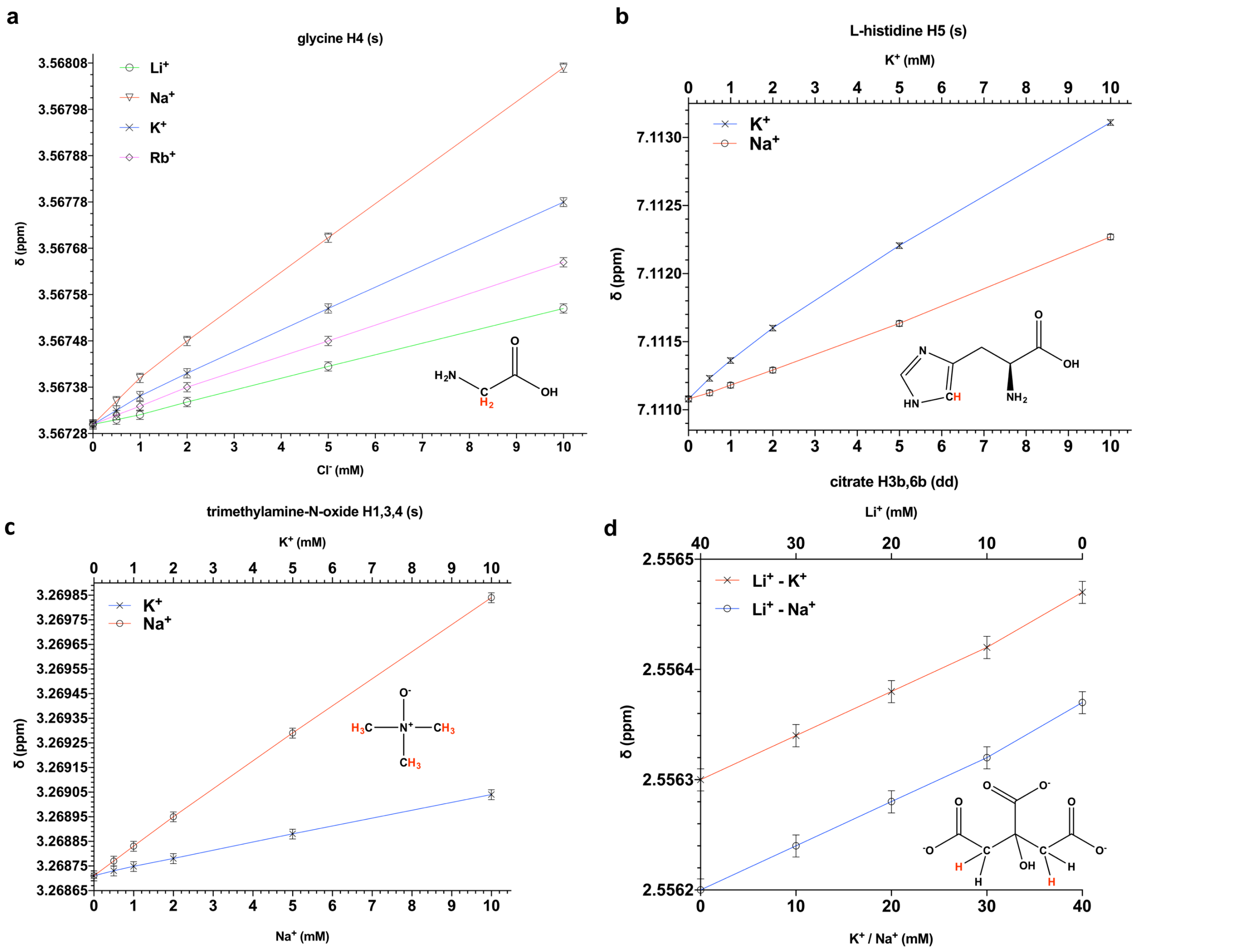

Supplementary Figure 6. Effect of titrating a mixture of metabolites with 4 ions (Li^+, Rb^+, Na^+ and K^+) all in the form of chloride salts. Variability of selected δ values of (**a**) glycine H4 protons as a function of all four ions; (**b**) L-histidine H5 proton, (**c**) trimethylamine-N-oxide H1,2,3 protons, and (**d**) citrate H3b,6b protons as a function of sodium and potassium. In case (**d**) the concentration of chloride is maintained constant by the addition of lithium chloride (see Supplementary Note 2).

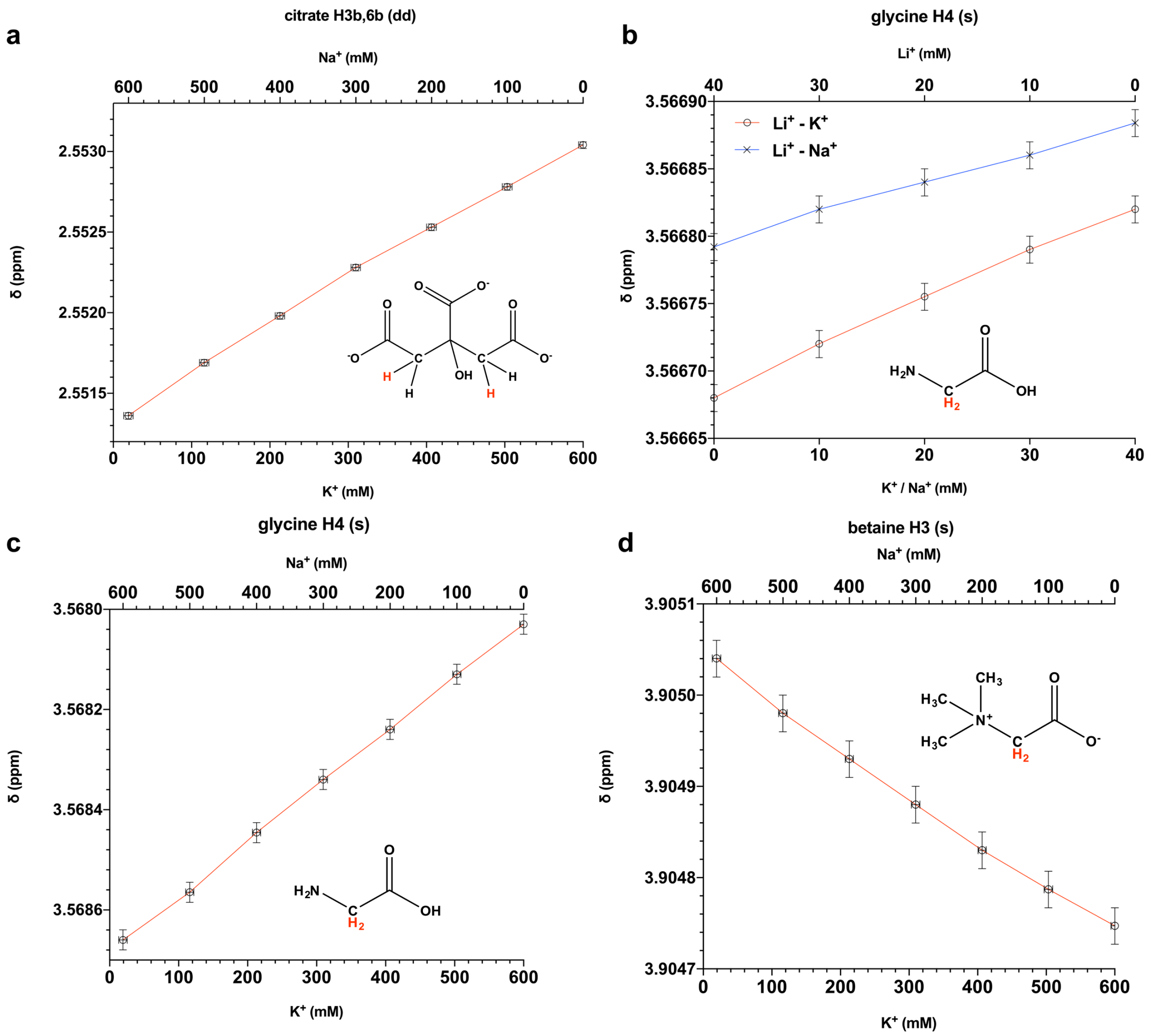

Supplementary Figure 7. Effect of titrating a mixture of metabolites with 3 ions (Li^+, Na^+ and K^+) all in the form of chloride salts, where the concentration of Cl^- is maintained constant. Variability of selected δ values of (**a**) citrate H3b,6b protons, (**b,c**) glycine H4 protons and (**d**) betaine H3 protons (see Supplementary Note 2).

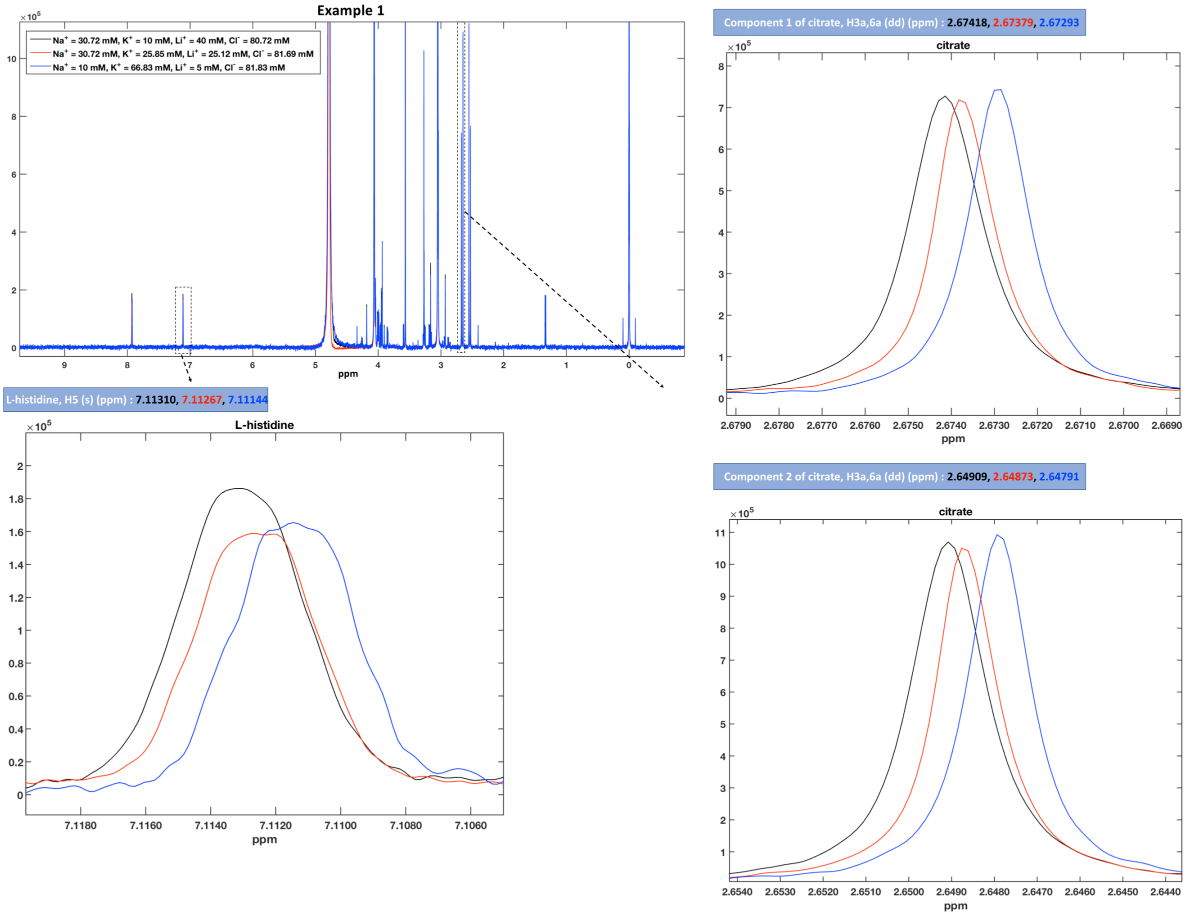

Supplementary Figure 8. Examples of metabolite signal positions in the presence of three ions (Li^+, Na^+ and K^+) all in the form of chloride salts, where the concentration of Cl^- is virtually constant (see Supplementary Note 2).

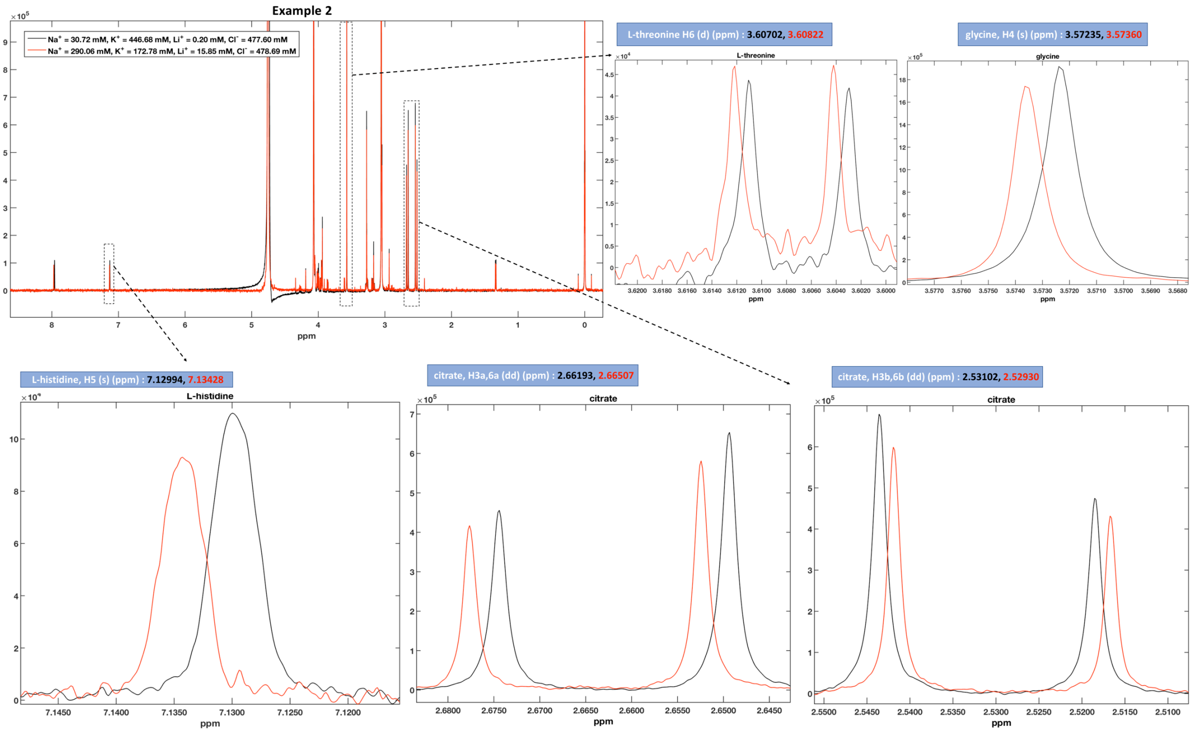

Supplementary Figure 9. Examples of metabolite signals positions in the presence of three ions (Li^+, Na^+ and K^+) all in the form of chloride salts, where the concentration of Cl^- is virtually constant (see Supplementary Note 2).

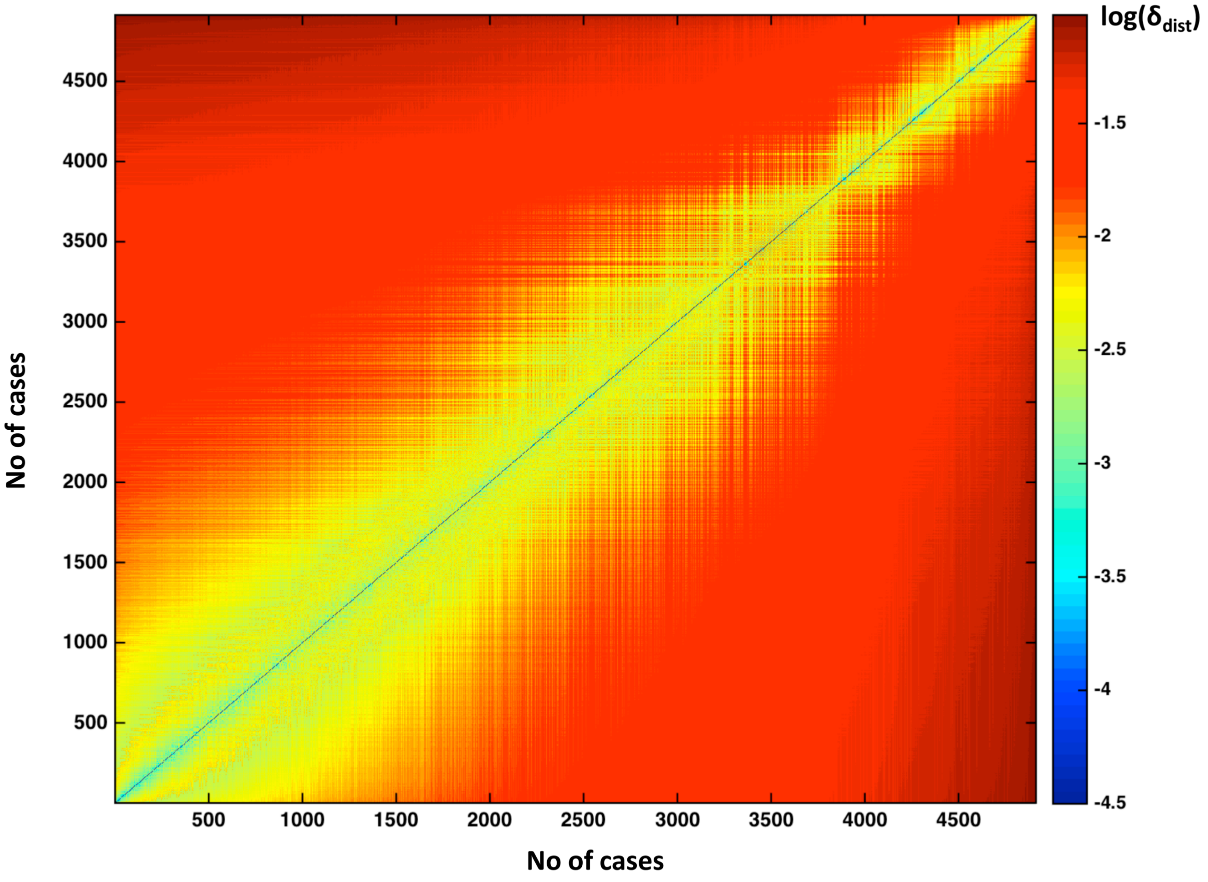

Supplementary Figure 10. Matrix of distances (δ_{dist}) in the 11th dimensional δ space showing that all distances are larger than 0.1 ppb or 0.0001 ppm [i.e log(0.0001) = -4] (see Supplementary Note 2).

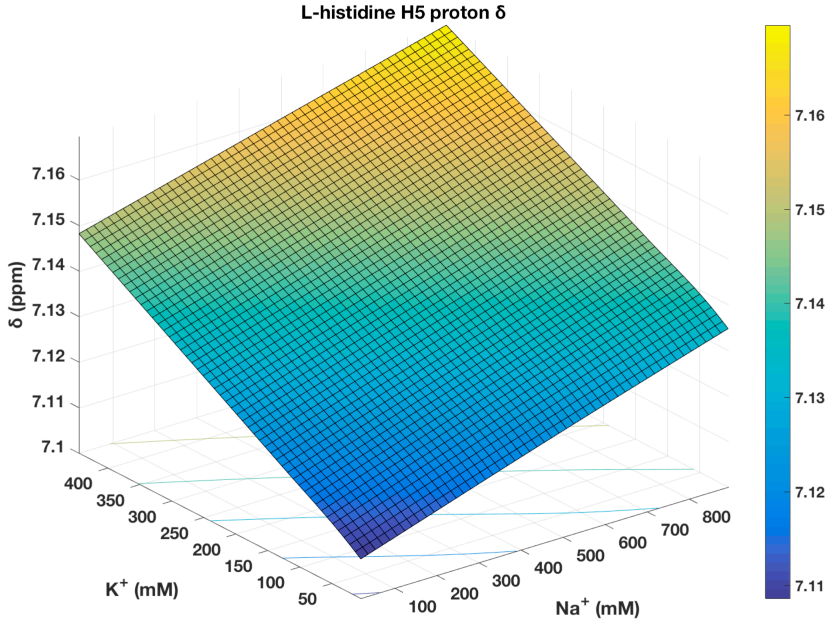

Supplementary Figure 11. Relationship among the concentrations of K^+ and Na^+ and the δ values of L-histidine H5 proton. The interpolated δ values are obtained from the constructed statistical predictive model based upon the 125 spectra, described in the above Supplementary Table 5. From the figure, it is apparent that the chemical shift of a single metabolite, even if highly variable, cannot be used to unambiguously predict the concentrations of two different ions (K^+ and Na^+ in this example) (see Supplementary Note 2).

Supplementary Note 1

Construction of the Predictor

The total number of the acquired artificial urine spectra, namely 4501 spectra (Fig. 2) constituted the spectral matrix. Sample NMR spectra of some of the mixtures are reported in Supplementary Fig. 1a-j. The significant spread of the chemical shifts (δ) of various metabolites in the different mixtures can be immediately appreciated. The minimal and maximal δ values observed for the various spin systems of each metabolite are also reported in Supplementary Table 1. All together, these values provide the boundaries of the multidimensional chemical shift space from which the chemical shift matrix was constructed. In particular, at least one ^{1}H NMR signal of each active and passive metabolite was assigned in the 4501 spectra, and their chemical shift values were recorded till the 5th decimal of ppm in each case. After the assignment, a multidimensional shift matrix was built as summarized in Supplementary Table 2.

Finally, a δ matrix was constructed where each chemical shift of each spin system of each metabolite in each mixture is related to the concentrations of all metabolites and ions etc. in that mixture, i.e. to a specific point in the multidimensional concentration space.

Predicting chemical shifts from concentrations

Based on functional relationships (models) such as those reported in Supplementary Fig. 1k-s and described in detail in the Methods section, the δ values of at least one spin system for each of the selected metabolites were thus related to the complex pattern of metabolite and ion concentrations as well as pH and temperature. Furthermore, the δ matrix could be expanded by interpolating δ values at concentration values not present in the mixture, but still within the covered concentration ranges (Supplementary Fig. 1k-s). In this way, given any pattern of concentrations of the selected metabolites and ions (i.e. for any given point in the multidimensional concentration space), the δ of the spin systems of all examined metabolites become in principle predictable. The sizes of δ changes by changing concentrations of the components (metabolites and ions) and pH can be clearly appreciated (Supplementary Fig. 1k-s). The predictions were always found to be very accurate, as tested by preparing a number of additional mixtures with concentration values not present in the original artificial urine matrix (Supplementary Fig. 2a), and by predicting the δ of their metabolites before actually recording their NMR spectra (Supplementary Fig. 2b,e,h,k,n,q).

Predicting concentrations from chemical shifts

Having established a relationship between any point in the chemical shift space and the corresponding point in the concentration space, the reverse process is also possible, i.e., if the δ of a sufficient number of compounds are known, one can predict the concentrations (and pH and temperature) of the compounds in the mixture. This is a key point of this strategy: if one can easily and safely identify, in any given spectrum, a relatively small number of metabolite signals, among those with the strongest δ dependence on the concentrations of the mixture components, then one could reconstruct the pattern of concentrations of compounds giving rise to that mixture. From this pattern, the δ of all the other signals can be predicted. To achieve this, another set of predictive models were built that, given the δ values of the active metabolites, could reconstruct the concentrations of these metabolites (see Methods). After careful examination of all spectra from the active metabolites mixtures, and the ANOVA decomposition of the models that predict concentrations (see Methods and Fig. 3), it was concluded that five spin systems from three metabolites - two doublets of citrate, two singlets of creatinine and one singlet of glycine – had the desired properties of: being easily identifiable, being very sensitive to the concentrations of the

mixture components, and being differently sensitive to the concentrations of different components, ensuring the broadest coverage of the concentration space. These spin systems are termed navigator signals, as they guide the search of the point in the concentration space that corresponds to that particular mixture. If the point is close enough to the actual one, the prediction of all other δ from that starting point will be accurate.

Predicting chemical shifts from chemical shifts

A large number of tests was performed on the artificial mixtures, and the back-prediction of all δ from the δ values of the navigator signals – through the identification of the corresponding point in the concentration space – always resulted very accurate (see Methods, Supplementary Fig. 2d,g,j,m,p,s). It is worth noting that the prediction of the concentration of the inorganic ions was also highly accurate, despite these ions are invisible in the NMR spectra (Supplementary Fig. 3). This finding by itself would make NMR profiling of urine a powerful one-shot analytical method for as many as 11 different inorganic ions. Furthermore, we noticed that the δ of several metabolites could be predicted directly from the δ of the navigator signals (Supplementary Fig. 2c,f,i,l,o,r). In other words, a multidimensional matrix of δ – δ relationships could be constructed and used for direct δ predictions (see Methods). It turned out that in several cases these direct predictions were even more accurate than those obtained by passing through the concentration matrix. As shown in Fig. 4, for real urine samples both procedures are followed.

Supplementary Note 2

Further explanation / theory behind the prediction of ion concentrations

It is known that inorganic ions in urine span a very broad range of concentrations, and this is believed to be one of the main causes for the high variability of the observed chemical shift values. To illustrate this behaviour, we have performed titrations of a simple mixture of 12 selected metabolites (i.e. betaine, citrate, creatinine, glycine, glycolate, L-asparagine, L-histidine, L-serine, L-threonine, succinate, tartrate and trimethylamine-N-oxide) with Li^+, Na^+, K^+, and Rb^+, all in the form of chloride salts (Supplementary Tables 4-5). As expected, all metabolite signals shift downfield with increasing salt concentration (Supplementary Fig 6). This is due to the fact that all metabolites contain at least one donor atom that is able to weakly coordinate cations, thus causing a slight de-shielding effect. But it can also be appreciated that firstly, not all signals shift by the same amounts, and, secondly, not all ions have the same effect on a given signal. This observation explains why the chemical shift pattern of a complex mixture can be predictive of the concentrations of different cations. Namely, the H4 of glycine is more sensitive to Na^+ than to K^+ (Supplementary Fig. 6a), while H5 of histidine is more sensitive to K^+ than to Na^+ (Supplementary Fig. 6b). This can be understood by using the freshman chemistry qualitative concept of hard and soft[4]: in glycine the donor atom oxygen is hard and prefers the hard Na^+ ion, while in histidine the donor atom is nitrogen, which is softer than oxygen and prefers the less hard K^+ ion.

Of course, increasing salt concentration increases also ionic strength, and Cl^- may also play a role. Therefore, we have also measured the variations of chemical shifts of the artificial metabolite mixture in the simultaneous presence of two different salts (e.g. Na^+ and K^+), whose concentrations were varied in such a way as to keep the concentration of Cl^- constant. From Supplementary Fig. 7-9, the differential effects of the different cations at constant Cl^- concentration can be fully appreciated.

Finally, we created various mixtures of the above mentioned 12 metabolites along with 3 ions (Li^+, Na^+ and K^+) in 5 different concentrations (Supplementary Table 4), roughly covering the physiological range of these ions in urine. In particular, we created a matrix of 5 x 5 x 5 = 125 solutions (Supplementary Table 5), maintaining the metabolites concentration constant while modifying each time the concentration of each ion. It should be noted that for all cations the counter anion was Cl^-. Eventually, 125 NMR spectra were recorded and the chemical shifts of 11 signals from 9 metabolites were measured (till the 5th decimal of ppm) and employed to create a chemical shift (δ) matrix (size of 125 x 11). Accordingly, 11 δ models were created, employing 4 variables: the concentrations of the 4 ions (3 cations and 1 anion) in each mixture. These were used for the interpolation of extra 12 concentration values for each cation (in total 12 + 5 = 17 concentration values for each cation, i.e. 17^3 = 4913 concentrations). Consequently, a new δ matrix was created with size of 4913 x 11, and we found that in all 4913 cases at least one of the 11 chemical shifts values was different (more than 0.05 ppb) even when the concentration of only one ion was changed to the nearest value. To illustrate this, we report in Supplementary Fig. 10 a matrix of distances in the 11th dimensional δ space to show that all distances are larger than 0.1 ppb (10^{-4} ppm).

Several examples of the size of the chemical shift differences for different combinations of these three cations at virtually constant chloride concentration are given in Supplementary Fig. 8-9.

Finally, it should be noted that not even the most responsive signal (i.e. histidine H5 in Fig. 3) is able by itself to be used as a predictor of a pattern of concentrations of ions and other active metabolites. For example, in Supplementary Fig. 11 we show that the chemical shift of histidine H5 is already ambiguous even with respect to predicting the concentration of Na^+ with respect to K^+.

Supplementary References

1. Assfalg, M. *et al.* Evidence of different metabolic phenotypes in humans. *Proc. Natl. Acad. Sci.* **105,** 1420–1424 (2008).

2. Bernini, P. *et al.* Individual Human Phenotypes in Metabolic Space and Time. *J. Proteome Res.* **8,** 4264–4271 (2009).

3. Ghini, V., Saccenti, E., Tenori, L., Assfalg, M. & Luchinat, C. Allostasis and Resilience of the Human Individual Metabolic Phenotype. *J. Proteome Res.* **14,** 2951–2962 (2015).

4. Pearson, R. G. Hard and Soft Acids and Bases. *J. Am. Chem. Soc.* **85,** 3533–3539 (1963).
