# Supplementary material for: Deconvoluting interrelationships between concentrations and chemical shifts in urine provides a powerful analysis tool
Source: Nat Commun. 2017 Nov 21;8:1662. doi: 10.1038/s41467-017-01587-0 (PMC5698486; doi:10.1038/s41467-017-01587-0)
Supplement: Supplementary file 2 — Description of Additional Supplementary Files [file 41467_2017_1587_MOESM2_ESM.pdf]

Description of Additional Supplementary Files

File Name: Supplementary Data 1
Description: Concentration values of all metabolites in all artificial mixtures employed for the study. Each row represents a mixture and reports the concentration of all components included in that mixture, its pH as well as the temperature at which the NMR spectrum was recorded (in total: 4501 artificial urine spectra).

File Name: Supplementary Data 2
Description: Chemical shift values predicted by three different routes (δpred) for the 50 spin systems of 36 "active" metabolites present in 40 randomly prepared artificial urine samples vs. their observed (δobs) values. The first route was from the metabolites-ions concentration and pH values, the second directly from the navigator signals δ values, and the third one from the metabolites-ions concentration and pH values which were previously predicted from the δ values of the navigator signals.

File Name: Supplementary Data 3
Description: Predicted δ values (δpred) for the 94 spin systems in 120 urine samples vs their observed δ values (δobs). The δobs values were checked by spiking each metabolite in each urine sample.

File Name: Supplementary Data 4
Description: Results of testing the predictor on a large urine dataset (1600 spectra) available from previous studies. The table reports only the predicted chemical shifts for which the assignment was unambiguous in each spectrum.
